# The Limpopo Non-Metropolitan Drinking Water Supplier Response to a Diagnostic Tool for Technical Compliance

**DOI:** 10.3390/ijerph14070810

**Published:** 2017-07-19

**Authors:** Avhashoni D. Nefale, Ilunga Kamika, Chikwelu L. Obi, Maggy NB Momba

**Affiliations:** 1 Department of Environmental, Water and Earth Sciences, Water Care Unit, TUT, Private Bag X680, 175 Nelson Mandela Drive, Arcadia Campus, Pretoria 0001, South Africa; NefaleA@dws.gov.za; 2 Department of Environmental Sciences, University of South Africa, UNISA Florida Campus, Christiaan de Wet/Pioneer Dr. P.O. Box X6, Florida 1710, South Africa; alainkamika@gmail.com; 3 University of Fort Hare, Alice 5700, South Africa; c355251@gmail.com

**Keywords:** drinking water, water quality, water management, Limpopo, South Africa, non-metropolitan city

## Abstract

Water services providers should supply water that is fit for human consumption, taking into account multi-barrier approaches and technical aspects such as design aspects, operation monitoring, final water quality compliance monitoring, plant monitoring practices, maintenance, and risk management practices. Against this background, this study focused on applying the diagnostic tool for technical compliance as well as assessing the compliance of water treatment plants with management norms. Six plants in the Vhembe District Municipality were selected; the Vondo, Malamulele, Mutshedzi, and Mutale plants (conventional), and the Dzingahe and Tshedza package plants. During the first assessment, four (Malamulele, Mutshedzi, Mutale and Dzingahe) plants scored between 44% and 49% and achieved Class 3 certification, revealing serious challenges requiring immediate intervention. Two water plants (Vondo and Tshedza, scoring 53% and 63%, respectively) were in the Class 2 category, revealing serious challenges requiring attention and improvement. During the second assessment, all plants scored between 63% and 87% (Class 2 category). The greatest improvement (30%) was noted for the Dzingahe and Tshedza plants, followed by the Malamulele plant, while the Mutale, Vondo, and Mutshedzi plants improved their scores by 20%, 17% and 14%, respectively. After corrective actions and re-measurement, no plant complied. It is recommended that Water Services Providers (WSPs) regularly apply the diagnostic tools and water safety plans as developed in order to comply with applicable standards.

## 1. Introduction

Water is an essential component that is required to sustain the lives of living beings, but when it is contaminated, it leads to public health challenges [1]. Ensuring access to safe drinking water is a determining factor for the health and wellbeing of all consumers [2]. Water is regarded safe for domestic consumption when its quality is acceptable in terms of physical, chemical and microbiological parameters [3]. In order to remove chemical and microbial pollutants, water sources must be subjected to treatment processes prior to human consumption [4]. Multiple barriers consisting of appropriate sanitation facilities, protection of raw water resource quality (starting from the catchment scale to the point of treatment and to the point of use) [5], drinking water treatment processes, and control of contaminants, coupled with the protection of distribution channels and plumbing systems, are necessary to effectively prevent the spread of waterborne diseases [2]. Four generally accepted basic processes for adequate purification of drinking water include: coagulation, flocculation, sedimentation, filtration and disinfection [6,7,8]. If only one barrier has to be considered, disinfection has to occupy the first place, unless evidence exists that chemical contaminants are more harmful than the risk from ingestion of microbial pathogens. Among the disinfection processes, ozonation, ultra violet (UV) radiation, ultrasonic heating, and chlorination (e.g., use of calcium hypochloride, sodium hypochloride and gas chlorine) are commonly used [9].

South Africa is regarded as a water-scarce country, and the consumer tap water quality for drinking in non-metropolitan areas is still questionable. Most of the drinking water treatment works in these areas do not treat water according to required or acceptable world standards [5,10,11]. Continuous contamination of water resources due to inadequately treated wastewater or human activity remains a great challenge for the country [10,12,13,14,15]. Even though the government is committed to improving access to safe water, a pledge that is leading to a significant reduction in the number of people without access to a reliable source of safe drinking water, water shortages still represent a risk to the achievement of this commitment.

Study has shown that populations with low income residing especially in rural areas still rely on untreated surface or underground water supplies [16]. For those who have improved piped systems, supply of drinking water is usually performed through small water treatment plants. In the last decade, investigators have reported that these water supply systems are generally ineffective for producing safe drinking water. This is due to a number of technical and management issues [11,12,17,18]. The technical issues include a lack of basic knowledge of water treatment principles (e.g., determination of the flow rate of water, calculation of coagulant and chorine dosages, determination of chlorine demand of the water, measurement of free chlorine residual concentrations in the final water, and measurement of the turbidity and pH of the water) by the operators of small water treatment plants. Furthermore, a lack of understanding of process selection and design of water treatment plants by the operators, inadequate financing for operation and maintenance, frequent shortages of coagulants and disinfectants, and lack of water quality monitoring and evaluation programmes have been also pointed out as being among the technical problems facing rural drinking water supply systems [8,10,12,19,20].

To assist small water treatment plants in rural areas to comply with the acceptable water quality standard as set by South African National Standard [21], a diagnostic tool was developed in 2009 by Momba and Swartz [22]. This tool is aimed at assisting to establish the reasons for non-compliance and to provide remedial measures that can be used to achieve compliance. The diagnostic tool for technical compliance is used to perform an assessment of technical treatment plant measurements, and includes: (1) design aspects; (2) quality control; (3) process control; and (4) plant monitoring, maintenance aspects and risk management. The main focus point is to measure the chemical and microbiological quality of the final water against the South African National Standard (SANS 241) specifications for drinking water [22].

For the purpose of this study, the diagnostic tool for technical compliance was applied in selected drinking water treatment plants of the Vhembe District Municipality (VDM) situated in the Limpopo Province in order to ascertain their responses to this diagnostic tool and evaluate their compliances with the national standards.

## 2. Materials and Methods

### 2.1. Description of the Study Area

**Figure 1:**
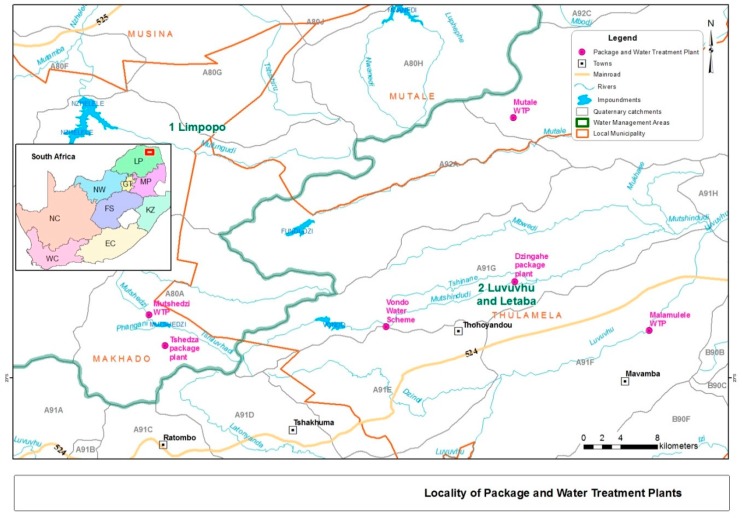
South African map with the locations of water treatment plants.

This study was undertaken at small drinking water treatment plants situated in three of the four local municipalities (Figure 1) of the Vhembe District, namely the Thulamela Local Municipality (Vondo Water Scheme, Malamulele Water Treatment Plant and Dzingahe Package Plant), the Makhado Local Municipality (Mutshedzi Water Treatment Plant and Tshedza Package Plant) and the Mutale Local Municipality (Mutale Regional Water Scheme). The Vondo Water Scheme, which is located in the Phiphidi village along the road from Sibasa to Nzhelele, treats 52 ML/day of water from the Vondo Dam. The Malamulele Water Treatment Plant is located at Malamulele village, approximately 20 km south-east of Thohoyandou town and ±5 km from the Nandoni Dam; the plant treats 16 ML/day. The Dzingahe Package Plant falls under the Thulamela Local Municipality and is found less than 5 km along the road from the Sibasa to Vondwe village and within 50 m of the banks of Mutshindudi River, which is a tributary of the Luvuvhu River. The plant purifies 0.4 ML/day, which is supplied to the Dzingahe village, with a population of about 10,000. The Mutshedzi Water Treatment Plant purifies 13 ML/day of water and is situated in Nzhelele, along the road to the Mauluma (Phadzima) village under the Makhado Local Municipality.

The plant started treating water in 1989. The Mutale Regional Water Scheme is found next to the Tshandama village under the Mutale Local Municipality. The plant pumps the water from the Mutale River; it is pumped electrically and then stored into the raw water dam at the plant. This plant serves ±38 villages in the Mutale area and treats 13.04 ML/day. The Tshedza Package Plant is located at Nzhelele (Tshedza village) under the Makhado Local Municipality along the road from Mandala to the town of Makhado. This plant treats 1.5 ML/day and supplies water to five villages, constituting a population of about 4000 people. All these plants fall under the Vhembe District Municipality, one of the water services authorities in the Limpopo Province. The small water treatment plants are defined in terms of the volume of water treated, at less than 52 ML/day.

### 2.2. Application of the Diagnostic Tool for Technical Assessment

**Figure 2:**
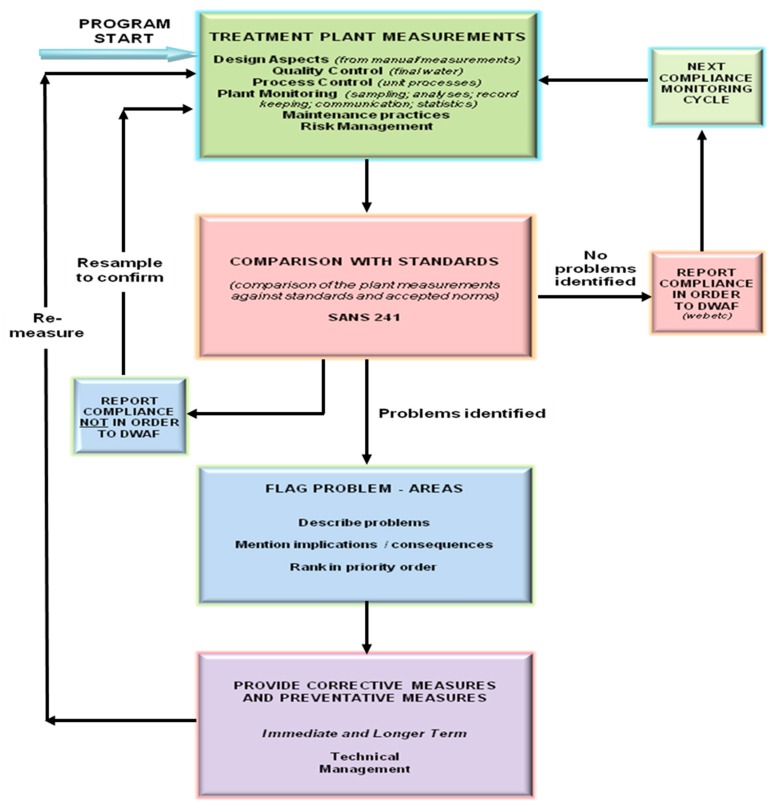
Technical diagnostic tool framework developed by Momba and co-workers (2009) to assess compliance of small water treatment plants. DWAF: Department of Water Affairs and Forestry.

The diagnostic tool developed by Momba and Swartz [22] was used for the technical assessment of the selected drinking water treatment plants, which also included the evaluation of the compliance of the provision of drinking water from the source to the consumers’ tap (point of use). The first assessment was performed between August 2008 and June 2009, while the second assessment was done between November and December 2010. Figure 2 illustrates the framework for the diagnostic tool for technical compliance developed by Momba and Swartz in 2009. The assessment of the selected plants was conducted by taking into consideration this framework and by following the step-wise procedures for technical compliance assessment as recommended by Momba and Swartz [22]. Based on the technical step-by-step diagnostic tool framework, a developed questionnaire [22] focusing on detailed assessment of technical treatment plant measurements was used. The process flow starts from the abstraction point to the treatment plant, including the design aspects of the treatment plant, quality control, process control, plant monitoring, maintenance and practices, risk management, comparison of the results with the acceptable drinking water quality standards, flagging of the areas where problems are identified, and provision of corrective actions. This questionnaire included the following aspects: design aspects, quality control, process control, plant monitoring, maintenance aspects, and risk management. Structured interviews were conducted to the area managers, plant superintendent and process controllers, based on the questionnaire as reflected in Table 1.

The target informants included the process controllers, general workers employed as process controllers, senior plants operators, superintendents and area managers. The research team worked closely in collaboration with the water services authorities and water services providers in the Vhembe District Municipality and the laboratory technicians [23]. During the study period, the flagged problem areas were identified and ranked in priority order. Each of these problem areas then received a score on a 1 to 5 scale according to the scoring system (Table 2) described by Momba and Swartz [22].

For each of the problem areas identified, corrective and preventive actions were taken. Thereafter, re-measurement was done between November and December 2010 by repeating the entire technical compliance assessment, focusing particularly on the problem areas that were identified during the first assessment and also establishing whether compliance had been restored. Prior to the re-measurement, a workshop was organised and all stakeholders from all the six drinking water treatment plants were invited. The main objective of this workshop was to highlight the problems areas identified during the first assessment and to discuss the corrective and preventive measures that should be taken in order to improve the component of the technical issues that impacted positively on the production of safe drinking water.

### 2.3. Drinking Water Quality Monitoring

The testing of drinking water quality was conducted in a monthly basis from August 2008 to December 2010 using standard methods [24] by the research team before and after the diagnostic tool was applied. The compliance of the drinking quality of the final water and drinking water at the point of use was measured against SANS 241 [21] drinking water quality specifications and South African Drinking Water Quality Guidelines for Domestic Use [25] based on the selected physico-chemical parameters (pH, turbidity, temperature and chlorine residuals) and indicator bacteria (*Escherichia coli* and total coliforms). The pH and the temperature were analysed using a pH probe and thermometer (model: Lovibond SensoDirect 200) at 25 °C, respectively, whereas the turbidity meter (model: DR/2000 spectrophotometer) was used to measure the turbidity of the water samples. Chlorine Tablets N Diethyl-1,4 Phenylenediamine (DPD) numbers 1 and 4 were dissolved into 50-mL water sample and a chlorine meter was used to measure residual chlorine (using a model DR/2000 spectrophotometer).

The enumeration of total coliforms and *E. coli* in the water samples was carried out using the Colilert™ 18 Quanti-Tray/2000 system (IDEXX Laboratories (Pty) Ltd., Johannesburg, South Africa) following the manufacturer´s instructions. The Colilert 18 method makes use of a multiple tube technique that gives the most probable number (MPN) of cells. *Escherichia coli* was detected after sealed quanti-trays were incubated at 37 °C for 18–24 h. Trays were examined under UV, wells that fluoresced were recorded, and the MPN of *E. coli* in the sample was inferred from the statistical table provided with the Colilert reagent, whereas a yellow colour indicated the presence of the total coliforms.

### 2.4. Scored and Weighted System for Technical Compliance Assessment

To determine the level of compliance or the problems that result in the compliance of the selected water treatment plants, the systematic evaluation of the overall performance of potable water supplies was rated using the technical compliance rating reflected in Table 3. The following formula was used to determine the total weight score of each plant:

$$Total\;weight\;Score=\frac{Total\;score\;obtained}{Maximum\;possible\;score}\times \left(Weight\right)$$

### 2.5. Statistical Analysis

The data was collected from the six selected drinking water treatment plants between August 2008 and December 2010. Water samples were collected from each water treatment plant from the raw water, filtered water, and point of treatment and at the point of use. Statistical data analysis was conducted using Stata computer software (version: STATA V12, Stata Corp. LP, College Station, TX, USA, 2015). The non-parametric Wilcoxon signed-rank test was performed to compare the data from the first assessment between August 2008 and June 2009 and the second assessment in November and December 2010.

## 3. Results

### 3.1. Assessment of the Design Aspects

#### 3.1.1. Mixing Equipment

The Vondo Water Scheme is a direct filtration plant and had no flocculation channels and no primary settling tank or sedimentation tank. This is a two-way plant, using: (1) a primary lime feeder—lime is added to adjust pH and sometimes pre-chlorination is done during dry season due to high iron and manganese; and (2) a secondary lime feeder—lime is added to adjust pH. Aluminium sulphate is added at the division box as a flocculent. During the study period, it was found that the process controllers were familiar with what they were dosing; they were able to calculate and adjust the dosing rate although they were focusing on visual dosage of chemicals. This resulted in insufficient mixing step in spite of their familiarity with the coagulation and flocculation in terms of mixing equipment.

At Mutshedzi, when the lime feeder is working, lime is added to adjust pH. During the time of visit, the lime feeder was not working. The plant had pre-chlorination due to high iron and manganese and algae. As for the Mutale Regional Water Plant, it operates in two different ways (channels) namely, rapid gravity sand filtration and slow sand filtration. The lime is sometimes added to adjust the pH, however it was noted that during the visit, the lime was not dosed at the pre-stage because the pH meter was not at the required standard. The Malamulele plant does not have a pre-treatment process; there are no iron, manganese or algae problems encountered. In three of the above-mentioned water treatment plants Princo 5306 was added at the mixing chamber as a polyelectrolyte. The Tshedza and Dzingahe Package Plants treat water in a closed system whereby Hydro care coagulant and soda ash were added in the floc column to correct the pH and the mixing inside the chambers was not visible. In all water treatment plants, all chemicals were dosed at the correct dosing points, the dosing rates were assumed and the jar tests were not performed at the plants.

#### 3.1.2. Flocculation Equipment

In the Mutale Regional Water Plant and the Malamulele and Mutshedzi Water Treatment Plants, due to visual dosage of chemicals, the floc formation and the floc growth observed were not good and they tended to break and not settle effectively. From time to time, there was a need for polyelectrolyte to be added to improve the settleability and strength of the floc. The dosing system was in working order and the dosing rate could not be adjusted while the back-up services were in place. The process controllers were able to operate and perform basic maintenance on dosing pumps. As to the Vondo Water Scheme and the Tshedza and Dzingahe Package Plants, the flocs were not observed because the treatment plants were using closed systems.

#### 3.1.3. Sedimentation Equipment

The Vondo Water Scheme has no sedimentation tank, it is a direct filtration plant. There are no flocculation channels and sedimentation tanks. At the Mutale Regional Water Plant and the Malamulele and Mutshedzi Water Treatment Plants, flocs had tendency to settle very well at the sedimentation tanks; there were also disludging valves. However, it was observed during the assessment period that there was an absence of short-circuiting in the sedimentation tank. The retention time was noted to be sufficient and the wind and the sun could not have a negative effect on these plants. It was also noted these plants had no problem with algae growth, the tanks could be straightforwardly cleaned. The sludge draw-off facilities were adequate and could be disposed of in an acceptable manner. At the two package plants of Tshedza and Dzingahe, sedimentation occurs in a tank like cylinder because the process is not visible.

#### 3.1.4. Filtration Equipment

The Vondo Water Scheme uses rapid gravity sand filters and water from the dosing chambers goes straight to this filters. During the assessment period, the filtration process was found to be effective. The operation of valves, the robustness of filter nozzles, and the backwash pump were found to be adequate and the correct filter media were used and protected against corrosion. At Mutale, Mutshedzi water treatment pre-chlorination is required to prevent algae growth, the process controllers add lime or soda as to balance the pH. It was noted that the costs of the civil structures were not known by the process controllers in any of the water treatment plants because all the facilities are old. The filtrate was not measured in any of the water treatment plants. With regard to the length of the filter runs, the process controllers were not able to maintain them under varying loadings, however the backwash was found to be performed on a routine basis and when certain head loss was reached. The quality of filtered water was not monitored in any of the water treatment plants.

#### 3.1.5. Other Important Aspects

Generally, the water treatment works are designed such that there is sufficient protection against extreme temperatures and weather conditions, furthermore all materials were quality checked and tested. In all water treatment works, there was provision of sufficient land that could be used for possible expansions, sufficient management and operations to ensure long-term sustainability. There were also well-established access routes to the drinking water treatment plants.

#### 3.1.6. Scoring of the Design Aspects

The scores and weight attained during the study period for the design aspects of the six selected water treatment works are shown in Tables 9 and 10. Four (4) of the selected water treatment plants (The Malamulele Water Treatment Plant, the Mutshedzi Water Treatment Plant, and the package plants of Tshedza and Dzingahe) scored each 8% out of a possible 10% score, whereas the Vondo Water Scheme and Mutale Regional Water Treatment Plant scored 7% during the first assessment. While an improvement of 1% was observed during the second assessment for the Vondo Water Scheme, the Mutshedzi Water Treatment Plant, the Mutale Regional Water Treatment Plant and the package plants at Tshedza, no changes were recorded at the Malamulele Water Treatment Plant and the package plants in Dzingahe. These plants had a similar score during both assessment periods.

### 3.2. Assessment of the Operations Monitoring Practices

#### 3.2.1. Coagulation and Flocculation

To determine the optimum concentration of coagulant to be added to the raw water, the process controllers used visual chemical dosage because the jar test was not functioning and they were not sure even quantitatively of the dose of chemicals they were adding. However, theoretically, the process controllers gave a good impression that they are able to calculate the required dosages, adjusting and monitoring the dosing rates if the laboratory instruments were available and in good conditions, so in this case the jar test was not performed. They further indicated that they were familiar with the basics of coagulation and flocculation for water clarification. Furthermore, there was basically no programme in place to monitor the floc formation in any of the water treatment plants.

#### 3.2.2. Sedimentation

With regard to observation of the floc blanket, the process controllers do not observe this; nonetheless, the over flow weir was kept clean and the flow was observed to be distributed evenly throughout the channel.

#### 3.2.3. Filtration

The backwash was noted to be performing appropriately on a routine basis, according to the correct procedure and when certain head loss was reached. Nevertheless, the quality of the filtrate and the turbidity breakthrough were not monitored in any of the water treatment plants.

#### 3.2.4. Disinfection

The chlorine was not dosed according to the chlorine demand of the water. Nevertheless, it was dosed at the suggested frequency and the stability of the final water was also determined although it was based on assumptions.

#### 3.2.5. Stabilisation

It was found that none of the process controllers were familiar with the notion of stabilisation and the reasons for stabilization, or how it should be affected and controlled; hence, this was not determined.

#### 3.2.6. Scoring of the Operations Monitoring Practices

The scores obtained for the assessment of the operations of monitoring practices are summarized in Tables 9 and 10. During the first assessment between August 2008 and June 2009, both Malamulele Water Treatment Plant and the Mutale Regional Water Treatment plant scored 8% out of a possible 20% and improved by 3% and 7%, respectively during the second assessment which was conducted between November and December 2010. As for the Mutshedzi Water Treatment plant and the packages plants at Dzingahe, they scored 8% in the first assessment and 11% and 14% respectively in the second assessment, with an improvement of 3% and 6%. The Vondo Water Scheme had a weight score of 13% in first assessment and dropped by 4% in the second assessment. However, the package plants in Tshedza improved by 3% with 11% scores during the first assessment and 14% in the second assessment. It can therefore be confidently stated that the improvements attained during the second assessment was due to the application of a diagnostic tools which served as a guidance applied for plant operation.

### 3.3. Assessment of Plant Monitoring Practices and Scoring Process

The research team monitored the water treatments in order to obtain the practical activities they perform daily in various unit processes. The process controllers were interviewed about the monitoring practices and they provided information regarding the chemical measurements, flow measurements, and interpretation of turbidity, pH and temperature in their case for raw water and final water because they do not measure the filtrate water. The research team also established a monitoring programme during the study period and inspected the conditions of the infrastructures as well as the equipment. The VWS, the Mutale Regional Water Treatment plant, the Mutshedzi Water Treatment Plant and the package plants in Dzingahe scored 8% each out of a possible 10%, while Malamulele Water Treatment Plant scored 23% during the first assessment with the exception of the package plant in Tshedza which had a score of 30% during the first assessment between August 2008 and June 2009 and the second assessment between November and December 2010. It was noted that process controllers were able to ensure the functioning of the laboratory equipment that were in good condition by selling empty chemical containers to community members and purchasing batteries for the running of the laboratory equipment. Furthermore, during the second assessment the VWS and package plants in Dzingahe scored 23% while Mutale Regional Water Treatment Plant and Mutshedzi Water Treatment Plant had a similar score of 23%.

### 3.4. Assessment of the Compliance (Final Water Quality) Monitoring Practice and Scoring Process

In 20% of all drinking water treatment plants assessed, the laboratory equipment such as the pH probe, turbidity meter, thermometer, jar test, and chlorine comparator or chlorine meter were available and in good working condition. Process controllers were able to conduct the jar test, however during the study period this was not performed due lack of experience, relevant knowledge, and skills and training; it is the officials from the District office who conduct the jar tests periodically. Although in 80% of the plants similar equipment could be found in working condition, a lack of funds available to purchase batteries hampered the water quality monitoring. As stated above, none of the selected plants could provide evidence of water quality data during the study period. Consequently, a monitoring programme for water quality was established in terms of physicochemical parameters (such as turbidity, temperature, pH and residual chlorine) and also for indicator coliform bacteria (total coliforms and *Escherichia coli*) during the assessment period.

Table 4 illustrates the turbidity values of water from raw water to the point of use during the study period. No significant health risks associated with the transmission of infectious micro-organisms could be identified for the drinking water produced by the Mutale, Mutshedzi, Tshedza, Vondo and Malamulele Water Treatment Plants, as the target water quality range (TWQR) for the turbidity of the treated water fell within the limits (between 1 and 5 Nephelometric Turbidity Units [NTU]) set for drinking water quality for domestic consumption [25] in final water. High turbidity values (>10 NTU) were observed in the raw water produced by the package plant in Dzingahe and this impacted on the quality of treated water at the point of treatment (7.89 NTU), during the first and the second assessments.

In all the selected water treatment plants, the pH of the drinking water ranged between 6.27 and 7.81 as shown in Table 5, which denoted that the selected plants fell under Class 1 compliance as stipulated in SANS 241 [26] which is also within the target water quality range (6.0–9.0) for drinking water that is used for human consumption as prescribed in DWAF [25]. The temperature ranged between 25.5 °C and 30.5 °C during the first assessment and the second assessment (Table 5).

With regard to residual chlorine dosage, it was found that inadequate residual chlorine was dosed in all the plants. Monitoring practices such as the chlorine demand of the water was not effectively implemented in all plants as process controllers were not able to calculate the relevant dosages. From the observations shown in Table 6, the residual chlorine for final water was found to be ranging between 0.14 and 0.69 mg/L for all the plants in the first assessment. For water to be regarded safe for drinking, the residual chlorine must be less than 0.5 mg/L in final water and water at the distribution channel should have residual chlorine concentrations less than 0.20 mg/L [26]. However, the results obtained during the second assessment revealed that the residual chlorine ranged between 0.10 and 1.50 mg/L at the point of treatment, whereas the results ranged between 0.14 and 0.75 mg/L at the point of use.

Table 7 reveals that during the first assessment and the second assessment, the total coliforms ranged between 485.80 MPN/100 mL and 1011.2 MPN/100 mL for raw water, while for filtered water the total coliforms were found to be between 220.00 and 868.80 MPN/100 mL water. At the point of treatment the number ranged between 133.30 MPN/100 mL and 466.80 MPN/100 mL, and at point of use between 158.00 and 387.00 MPN/100 mL. In general, these maximum counts by far exceeded the recommended limits (100 MPN/100 mL) in terms of the total water quality range [25]. These results suggest poor water treatment or there might be bacterial re-growth in the distribution channels. As to the presence of *Escherichia coli* during the first and the second assessment periods, the counts of this indicator bacteria in the raw water ranged between 145.00 and 880.90 MPN/100 mL, while for the filtered water and treated water at the point of treatment the *E. coli* count ranged between 84.00 MPN/100 mL and 445.00 MPN/100 mL and at the point of use the results it ranged from 77.40 MPN/100 mL to 171.40 MPN/100 mL from all the water treatment plants.

These revealed increased risk of viral and bacterial infections such as gastroenteritis, hepatitis, *Vibrio cholera*, and many more [25] and this was due to the various activities taking place upstream of the abstraction point such as animal defecation, lack of proper ablutions facilities, and dumping of waste illegally that may lead to high water pollution.

Table 8 summarizes the number of samples taken from the selected plants. The water quality monitoring was performed more during the second assessment compared to the first assessment and was done to ascertain whether the plant managers took into consideration the corrective measures that were discussed during the workshop that was held at the end of first assessment and if these could measure the performance of the treatment plants. In spite of this discrepancy, the standard deviation of each set revealed whether or not this had an impact of the mean values used to compare the two study periods. Statistical evidence revealed a significant difference only for the turbidity data (*p* = 0.0203), whereas no significant differences were noted among the residual chlorine, coliform bacterial group, temperature and pH data (*p* > 0.05). Increases in turbidity levels dramatically increased the residual chlorine concentrations in treated water. This resulted in the deterioration of the plants performance.

As can be seen in Table 9 and Table 10, during the first assessment, the Tshedza package plant had a higher score (26%), followed by the Malamulele Drinking Water Plant (23%). A similar score of 15% were assigned to the Vondo Water Scheme, Mutshedzi Water Treatment Plants, the Mutale Regional Water Treatment plant and Dzingahe Package Plant. With the exception of Tshedza Package Plant and Mutale Regional Water Treatment Plant, a great improvement in terms of final drinking water quality compliance was noted for the four remaining plants during the second assessment (Table 9).

### 3.5. Assessment of the Maintenance Practices

#### 3.5.1. Sophistication of Equipment

During the execution of the study, it was revealed that equipment found in the plant laboratories or plant facilities was easily handled by plant operators. It was found that plants operators could undertake general operation and maintenance if they were trained or if they had relevant skills. In-house as well as external training for plant operators were performed in every plant.

#### 3.5.2. Availability of Spare-Parts

While back-up services for emergency cases were in place in all the water treatment plants, there were no spare-parts available or kept on-site during the study execution. However, if there was any broken equipment, it was immediately reported by process controllers to the area managers and the matter was attended to with urgency. Minor maintenance could be done by process controllers on-site while major maintenance was normally outsourced to the professional service providers.

#### 3.5.3. Availability of Back-Up Services

Generally, in every plant back-up services were readily available. These included specifically the gas chlorine and granular chlorine. However, if a major service is required in an emergency state, the District Municipality is well able to react as soon as the service is required, or provisions are made in cases where the service may take long. The community members are not able to perform some aspects of the service themselves as they are not trained to treat water except in cases where there is a burst pipe, where they are able to find ways of blocking such water from spilling and report to the local municipality offices.

#### 3.5.4. Communication Facilities

With regards to communication facilities between the process controllers and the management, it was revealed that the relationship was good and this also extends to the suppliers and the consultants. Any room for improvement was welcomed.

#### 3.5.5. Ability to Perform Own Maintenance

The study showed that the community members were able to perform their own maintenance especially in their households. Lack of funding does not allow the extension of training opportunities to community members. Priority is then given to process controllers for smooth running of the water treatments plants.

#### 3.5.6. Access to Plants

The access to water treatment plants was found to be good; even the delivery trucks are able to enter the premises. The process controllers pointed out that they have no idea of the financial provisions for upgrading such roads.

#### 3.5.7. Availability of Funds for Maintenance

Overall, it was also found that there were no sufficient funds allocated for maintenance. Nevertheless, a provision was made when there were urgent matters to be attended to. Process controllers were not sure if there were alternative sources of funding for maintenance.

#### 3.5.8. Scoring for Maintenance Practices

The scores for maintenance practices revealed that the Malamulele Water Treatment Plant, and Tshedza and Dzingahe Package Plants scored 6%, whereas the Vondo Water Scheme, and Mutshedzi and Mutale Regional Water Treatment Plants obtained scores of 10%, 12% and 10%, respectively. After implementation of tools, the six water treatment plants revealed an improvement for maintenance practices with both the Malamulele Water Treatment and Dzingahe Package Plants scoring up to 14%, Vondo and Mutale scoring up to 17%, and the Tshedza package plant scoring up to 16%. The Mutshedzi Water Treatment Plant was the plant with the poorest improvement as it scored 12% in the first assessment and 13% in the second assessment (Table 9 and Table 10).

### 3.6. Assessment of the Risk Management Practices and Scoring Process

The results shown in Table 9 and Table 10 revealed that there were no documented risk management systems in place during the study execution.

However, in all the plants it was indicated that there was a process in developing a draft water safety plan as this is a requirement by the Department of Water and Sanitation. In the meantime, the process controllers were able to identify the risk and to come up with mitigation measures and report immediately to the senior managers for urgent attention. For these reasons, all the water treatment scored 0% during the first assessment. During the second assessment, the Malamulele Water Treatment Plant, Vondo Water Scheme and Mutshedzi Water Treatment Plants obtained 7%, while the Mutale Water Treatment Plants and the two package plants (Tshedza and Dzingahe) scored 10% and this was due to the draft safely plans that were said to be available.

### 3.7. Comparing the Total Weight Scores during the First and the Second Assessment for Technical Compliance

As can be seen in Table 9 and Table 10, during the first assessment process, the overall scores for technical compliance for the Malamulele Water Treatment Plant, the Vondo Water Scheme, the Mutshedzi Water Treatment Plant, the Mutale Regional Water Treatment Plants and the Dzingahe Package Plant were found to be between 46% and 57%, which depicts Class 3 compliance. This compliance category suggests a need for serious and immediate interventions for these plants. Tshedza Package Plant was found to be the only plant that scored 61% and fell under Class 2 compliance category (50.83%), with serious challenges requiring attention and improvement. Although a great improvement in terms of technical compliance was observed in all plants, the highest improvement 28%) was noted in the Dzingahe Package Plant, followed by the Mutale Regional Water Treatment Plants (26%) and the Tshedza Package Plant (25%); the Malamulele Drinking Water Plant and the Mutshedzi Water Treatment Plant improved by 22%, and Vondo Water Scheme improved by 17% (Figure 2). This significant improvement led to all the plants to fall under Class 2 (72.83%) compliance as set by Water Services Providers (WSPs) and Water Services Authorities (WSAs) during the reassessment process.

### 3.8. Assessment of the Availability of the Laboratory Equipment

During the study period, a rigorous inventory of the laboratory equipment was performed. In 20% of the six drinking water treatment plants assessed, the laboratory equipment such as pH probes, turbidity meters, thermometers, jar tests, chlorine comparators, or chlorine meters were available in good working condition, and process controllers were able to conduct the jar. However, results of the survey revealed that 80% the equipment were available, in working condition but were not in use because of insufficient funding to purchase the batteries (Table 9 and Table 10).

### 3.9. Determination and Raking of Problem Areas in Priority Order

Table S1 depict the causes of the problems facing the selected drinking water treatment plants and their implication/consequences. During the study period, process controllers were interviewed on-site concerning the status of their water treatment plants. A workshop was held in Vhembe District offices and the feedback was provided on the problems areas identified during the first assessment. This resulted in a fruitful discussion with the municipal officials, including superintends and process controllers involved in drinking water treatment and management in the districts.

It was noted that all the drinking water treatment plants had similar problems, which included: (1) inadequate funding for maintenance of laboratory equipment; (2) lack of monitoring practices for the chlorine demand of the water; (3) lack of laboratory facilities for on-site monitoring of microbiological compliance of drinking water; (4) inadequate operations monitoring practice; and (5) lack of assessment of risk management practices (Table S2). In spite of the corrective measures that were taken after the notification of the problem areas to the WSA and the WSP, some of these problems still persisted in the selected water treatment plants during the second assessment. The overall weight scores for technical compliances revealed significant improvement between scores obtained during the first assessment and those obtained during the second assessment after the application of the diagnostic tool for technical compliance. This means that corrective measures were considered especially for water quality monitoring. Table S3 illustrates the ranking of the problem areas in order of priority. The moderate consequences were detected under the design aspects and plant monitoring practices and a major consequence was observed on risk management practices. All these categories were discussed with the municipalities for their immediate intervention.

## 4. Discussion

In order to avoid public health concerns related to waterborne diseases, it has been reported that proven that water for human consumption should be safe, containing no harmful concentrations of chemicals or micro-organisms, and ideally should have a pleasant appearance, taste and odour [25]. However, in some parts of SA, the quality of drinking water is still not trusted, due to poor water treatment practises. This leaves water not meeting local and international drinking water quality standards [11]. It has been revealed that the first step that needs to be taken into consideration is the proper selection of the water treatment systems for sustainable supply of safe drinking water, hence a thorough understanding of the processes and relevant training and knowledge to ensure the understanding of the whole value chain of treatment processes by process controllers [27] are necessary. For this reason, poor drinking water supply is usually attached to a number of technical factors such as design aspects, inability to calculate chlorine dosages, determine flow rate, estimate the free chlorine residual concentrations, measure and take readings of various variables, and repair or to perform basic maintenance of plant equipment, as well as insufficient funding for operation and maintenance and frequent shortages of chemicals such as coagulants and disinfectants.

Results obtained from the six selected drinking water treatment plants revealed that in terms of design aspects, the mixing intensity was not adequate due to lack of proper dosing calculations. Coagulation and flocculation in small treatment systems selected during the study period played an important role in the production of poor quality of the final water. Results of this study corroborate findings by Swartz [18] that revealed poor drinking water quality in small water treatment plants due to lack of the control of the chemical mixing and dosing processes. This author also pointed out incorrect placing of chemical dosing points, insufficient flocculation retention time, and allowances not being made for slower floc formation during winter months resulted in potentially poorer plant performance during the cold periods. Nevertheless, it was found that when relevant measuring instruments were effectively used or operational, the process controllers could be able to dose the chemicals correctly. Consequently, good flocs could be observed where sufficient chemicals were dosed.

The main purpose of the sedimentation process is the removal of readily settleable particles or coagulated impurities. If the correct dosing rates are applied, a floc blanket should be observed and settling velocity rate should also be adequate; there should be no short circuiting in the sedimentation tanks [22]. During the study period, in a number of the selected plants, the filtrate was not measured under normal peak loadings, but the backwash was performed properly and at the right times. Gas chlorine was found to be an effective disinfectant, especially for the package plants where granular chlorine was always in use. This was the determinant factor that resulted in high performance of this type of plants during the study period.

The quality of drinking water plays a major role in maintaining public health. It is generally affected by three main parameters which include: (1) physical/aesthetic; (2) chemical; and (3) microbiological aspects. Of these parameters, pH, turbidity, free chlorine, total and faecal coliforms are very important in the assessment of the quality of drinking water supplied by drinking water treatment plants to their consumers. The World Health Organization and the South African National Standards [21] specifications for drinking water point out that *Escherichia coli* and *faecal coliform counts* must be zero per every 100 mL of water sample tested [26,28]. In general, monitoring of the final water in all the selected plants was not done as required by the drinking water quality standards [26]. However, it has been stated that the water quality data can be used by decision makers as a trigger for immediate short term corrective measures for the improvement of drinking water quality [25]. In this study, lack of a proper water quality monitoring programme was noted in the selected plants, due to unavailability of basic measuring instruments, laboratory equipment, funding, and water treatment chemicals. Furthermore, for monitoring programme to be effective each water treatment plant should monitor, among other variables, pH, which has to be measured hourly. The turbidity should be monitored weekly for raw water, settled and filtered daily, and for final water every eight hours. The recommended motoring programme for residual chlorine is four-hourly and faecal coliforms are dependent on the population served by the plant.

This study therefore calls for an urgent intervention to provide process controllers with sufficient means for the execution of on-site monitoring programme for drinking water quality. It is important for the WSA and WSP to know that the most important aspect for improving the health of people is to provide safe and clean water to communities. Contaminated drinking water carries pathogenic viruses, protozoa and bacteria that cause several waterborne diseases such as cholera, typhoid fever, shigellosis, salmonellosis, campylobacteriosis, gardiasis, cryptosporidiosis and hepatitis A viral infections [25]. The use of total coliform bacteria is very important for assessing the quality of drinking water, whereas the presence of *Escherichia coli indicates* that the water is faecally contaminated and may indicate the presence of pathogenic viruses. Analysis of test water samples conducted during the study period indicated that the maximum counts for total coliforms and *E. coli* in some of the plants far exceeded the recommended limits. Lack of frequent monitoring of the microbial quality of drinking water in the selected plants is a matter of great concern as it prevents process controllers and the managers of these plants from being aware of the performance of their plants and in particular the quality of the drinking water supplied to their communities.

South Africa promotes right to free access to safe and reliable sources of water [29] and this challenges the capital requirement for water services operations and maintenance against the backdrop of inadequate production cost recovery. Inadequate funds for operations and maintenances were noted in the selected plants. This fact was also pointed out by previous investigators. Obi et al. [20] reported inadequate funding for operations and implementation activities as a huge disadvantage that hampers effective and efficient water service delivery in small water treatment plants of the Limpopo province. It is very important for the local WSA and WSP to know that the size of the plant should influence the funds allocation. An effective financial auditing system should be put in place in the selected plants for the assessment of financial matters, information sharing, budget monitoring, expenditure control mechanisms and procurement systems. Transparency in procurement aspects should also be regarded as critical and a success factor for the production of safe drinking water. The WSA or WSP should make financial provision adequate to the most critical aspects for good and acceptable water services delivery, provide sufficient funds for operations and maintenance and conduct proper cost benefit analysis [30]. The budgeting systems should also cover emergency funds for urgent repairs, procurement of equipment and chemicals, scheduled upgrading and extensions.

## 5. Conclusions

Compliance of small water treatment plants with accepted drinking water quality standards and management norms is still a challenge in the rural areas of South Africa. The study revealed that technically all the six water treatment plants that have been assessed were not able to meet the required drinking water standards. Of all the plants visited, only one was found to have the laboratory equipment in good condition and operational. This implies that the water that is supplied to consumers may pose a public health risk. There is a shortage of staff, especially skilled personnel, as well as a lack of measuring instruments/laboratory equipment, chemicals, and sufficient funds and water safety plans in place for the management of drinking water treatment plants. Thus, it was revealed that none of the plants were able to comply with the requirement for drinking water quality norms. In order for the water suppliers to provide water that is safe for domestic consumption, it is therefore crucial that all the WSAs and WSPs in the Vhembe District municipality train the process controllers internally and externally and also encourage them to apply the technical diagnostic tools developed and follow all the steps to improve the supply of drinking water to consumers. The development of water safety plans for all water treatment plans is also required. A proper implementation of this technical tool would ensure better blue drop scores for local municipalities.

## Figures and Tables

**Table 1:** Ranking of the problem areas in priority order.

Ranking of the Problem Areas in Priority Order	
1	Insignificant health consequence
2	Minor health consequence
3	Moderate health consequence
4	Major health consequence
5	Catastrophic health consequence

**Table 2:** Technical compliance assessment scoring [22].

Criterion	Weight %
Technical Compliance Scoring	
1A: Design aspects	0.1
1B: Operation monitoring practices	0.2
1C: Compliance (final water quality) monitoring practices	0.3
1D: Plant monitoring practices	0.1
1E: Maintenance practices	0.2
1F: Risk management practices	0.1
TOTAL	1.0

**Table 3:** Technical compliance rating of the treatment plants [22].

Total Weighted Score	Rating Description
0–50	*Class 3 Compliance:* Total compliance; serious and immediate intervention required (TAC)
50–90	*Class 2 Compliance:* Serious challenges requiring attention and improvement
90–100	*Class 1 Compliance:* Acceptable compliance

**Table 4:** Monitoring of turbidity (NTU) in selected plants.

Plant Name	Sampling Points/Sampling Period							
	First Assessment				Second Assessment			
	RW	FW	PoT	PoU	RW	FW	PoT	PoU
Turbidity (NTU)								
Vondo Water Scheme	3.01	0.99	1.44	1.39	3.73	1.45	1.86	1.81
Malamulele Water Treatment Plant	10.36	0.28	0.36	0.53	13.44	0.37	0.52	0.55
Mutale Regional Water Plant	5.04	0.40	0.82	0.78	4.63	0.72	1.88	3.10
Mutshedzi Drinking Water Plant	6.25	0.84	1.48	1.64	24.25	2.41	3.34	4.35
Tshedza Package Plant	4.29	None	0.91	1.24	16.23	None	1.46	2.55
Dzingahe Package Plant	19.61	None	4.03	None	40.65	None	7.89	None

RW: raw water, FW: filtered water, PoT: point of treatment, PoU: point of use and None: no water samples were collected.

**Table 5:** Monitoring of temperature and pH in selected plants.

Plant Name	Sampling Points/Sampling Period							
	First Assessment				Second Assessment			
	RW	FW	PoT	PoU	RW	FW	PoT	PoU
Temperature (°C)								
Vondo Water Scheme	27.6	27.4	27.2	27.4	25.5	25.8	26.6	27.2
Malamulele Drinking Water Plant	28.1	27.8	27.3	27.7	29.0	28.1	27.9	28.9
Mutshedzi Drinking Water Plant	27.9	28.3	27.3	28.6	26.9	27.9	28.6	29.6
Mutale Regional Water Plant	29.9	28.5	28.2	29.1	28.2	28.2	29.8	30.5
Tshedza Package Plant	27.1	None	27.8	None	27.8	None	28.0	30.5
Dzingahe Package Plant	27.5	None	27.9	29.9	30.9	None	29.6	None
pH								
Vondo Water Scheme	6.61	6.36	6.41	6.27	7.00	7.35	7.2	7.3
Malamulele Drinking Water Plant	7.26	7.45	6.63	7.04	7.24	7.52	7.12	6.89
Mutshedzi Drinking Water Plant	7.62	6.51	7.55	6.85	7.49	6.43	7.20	7.47
Mutale Regional Water Plant	7.54	6.45	6.64	6.76	7.60	7.35	6.20	7.60
Tshedza Package Plant	6.81	None	7.97	6.99	7.00	None	7.65	7.60
Dzingahe Package Plant	6.38	None	6.74	None	6.81	None	7.17	None

RW: raw water, FW: filtered water, PoT: point of treatment, PoU: point of use; None: no water samples were collected.

**Table 6:** Monitoring of residual chlorine (mg/L) in selected plants.

Plant Name	Sampling Points/Sampling Period			
	First Assessment		Second Assessment	
	PoT	PoU	PoT	PoU
Residual chlorine				
Vondo Water Scheme	0.37	0.23	0.60	0.35
Malamulele Water Treatment Plant	0.48	0.28	0.10	0.60
Mutale Regional Water Plant	0.62	0.44	1.50	0.75
Mutshedzi Drinking Water Plant	0.53	0.14	1.50	0.30
Tshedza Package Plant	0.43	0.24	0.20	0.20
Dzingahe Package Plant	0.69	None	1.00	None

PoT: point of treatment, PoU: point of use; None: no water samples were collected.

**Table 7:** Microbiological monitoring of water quality in selected plants: total coliforms and *Escherichia coli* (MPN/100 mL). MPN: most probable number.

Plant Name	Sampling Points/Sampling Period							
	First Assessment				Second Assessment			
	RW	FW	PoT	PoU	RW	FW	PoT	PoU
Total coliforms (MPN/100 mL)								
Vondo Water Scheme	625.50	342.00	236.60	212.20	485.80	220.00	133.30	170.00
Malamulele Water Treatment Plant	755.60	239.70	344.90	166.30	579.40	323.00	268.10	256.10
Mutale regional Water Plant	1553.1	868.80	377.10	387.90	495.00	293.00	172.00	158.00
Mutshedzi Drinking Water Plant	629.40	418.10	254.30	212.20	613.1	402.00	283.60	294.50
Tshedza Package Plant	1011.20	None	466.80	341.70	1011.20	None	427.30	211.90
Dzingahe Package Plant	960.60	None	454.00	None	791.5	None	304.20	None
*Escherichia coli* (MPN/100 mL)								
Vondo Water Scheme	230.00	110.00	98.10	90.00	145.00	120.00	84.00	77.40
Malamulele Water Treatment Plant	316.20	193.00	112.00	136.10	154.80	116.00	97.00	93.00
Mutale Regional Water Plant	659.16	445.00	193.00	171.40	308.40	183.00	108.00	98.30
Mutshedzi Drinking Water Plant	213.50	130.00	98.50	77.50	277.30	120.00	90.00	95.00
Tshedza Package Plant	880.90	None	213.50	112.10	697.60	None	255.70	108.30
Dzingahe Package Plant	501.10	None	328.40	None	548.00	None	120.00	None

RW: raw water, FW: filtered water, PoT: point of treatment, PoU: point of use; None: no water samples were collected.

**Table 8:** Statistical analysis of drinking water collected from different sampling points.

Group		No Observation	Mean	Std. Dev	*p*
Chlorine (mg/L)	First assessment	22	0.518	0.556	0.234
	Second assessment	44	0.398	0.259	
Turbidity (NTU)	First assessment	43	7.122	12.292	0.0203
	Second assessment	84	3.282	6.154	
Coliforms including *E. coli* (CfU/mL)	First assessment	48	197.621	342.664	0.1758
	Second assessment	96	310.446	520.421	
Temperature (°C)	First assessment	42	22.376	10.850	0.4800
	Second assessment	54	23.910	10.238	
pH (pH Unit)	First assessment	48	6.584	2.295	0.5391
	Second assessment	89	6.373	1.672	

CFU: Colony forming units; Std. Dev: Standard deviation.

**Table 9:** Technical assessment: first assessment: period between August 2008 and June 2009.

Name of Plant	Malamulele Drinking Water Plant	Vondo Water Scheme	Mutshedzi Water Treatment Plant	Mutale Regional Water Treatment Plant	Tshedza Package Plant	Dzingahe Package Plant
1A. Design aspect (10%)	8	7	8	7	8	8
1B. Operation monitoring practices (20%)	6	13	8	8	11	8
1C. Compliance (final water quality) monitoring practices (30%)	23	15	15	15	26	15
1D. Plant monitoring practices (10%)	4	6	4	6	6	10
1E. Maintenance practices (20%)	6	10	12	10	6	6
1F. Risk management practices (10%)	0	0	0	0	0	0
Total scores	47%	51%	47%	46%	57%	47%

**Table 10:** Technical assessment: second assessment: period between November and December 2010.

Second Assessment Name of Plant	Malamulele Drinking Water Plant	Vondo Water Scheme	Mutshedzi Water Treatment Plant	Mutale Regional Water Treatment Plant	Tshedza Package Plant	Dzingahe Package Plant
1A. Design aspect (10%)	8	8	9	9	9	8
1B. Operation monitoring practices (20%)	9	9	11	15	14	14
1C. Compliance (final water quality) monitoring practices (30%)	25	23	23	15	27	23
1D. Plant monitoring practices (10%)	6	4	6	6	6	6
1E. Maintenance practices (20%)	14	17	13	17	16	14
1F. Risk management practices (10%)	7	7	7	10	10	10
Total scores	69%	68%	69%	72%	82%	75%

## Supplementary Materials

The following are available online at www.mdpi.com/1660-4601/14/7/810/s1, Table S1: Technical compliance 2009 and 2011, Table S2: Technical compliance rating of the treatment plants during the first between August 2008 and June 2009 and the second assessment between November and December 2010, Table S3: Ranking of the Problem Areas in Priority Order after the first assessment.
