# Supplementary material for: The Limpopo Non-Metropolitan Drinking Water Supplier Response to a Diagnostic Tool for Technical Compliance
Source: Int J Environ Res Public Health. 2017 Jul 19;14(7):810. doi: 10.3390/ijerph14070810 (PMC5551248; doi:10.3390/ijerph14070810)
Supplement: Supplementary file 1 [file ijerph-14-00810-s001.doc]

**Table s1.** Technical compliance 2009 and 2011.

**18 august 2009**

1. MALAMULELE TREATMENT PLANT

| **Table 1.1**  **TECHNICAL COMPLIANCE SCORING** | | | | |
| --- | --- | --- | --- | --- |
| **Criterion** | | **Weight** | | |
| **1. TREATMENT PLANT MEASUREMENTS** | | | | |
| 1A: Design Aspects | | **0.1** | | |
| 1B: Operation Monitoring Practices | | **0.2** | | |
| 1C: Compliance (Final Water Quality) Monitoring Practices | | **0.3** | | |
| 1D: Plant Monitoring Practices | | **0.1** | | |
| 1E: Maintenance Practices | | **0.2** | | |
| 1F: Risk Management Practices | | **0.1** | | |
| **TOTAL** | | **1.0** | | |
| **Table 1.2**  **TECHNICAL COMPLIANCE SCORING:** | | | | |
| **Criterion** | | | **Yes =1** | **No = 0** |
| **1. TREATMENT PLANT MEASUREMENTS** | | | | |
| **1A: Design Aspects** | | | | |
| Rapid Mixing | Is the mixing intensity sufficient? | | 1 |  |
|  | Is the mixing time sufficient? | | 1 |  |
|  | Are chemicals dosed at the correct points? | | 1 |  |
| Flocculation | Is flocculation intensity at correct levels (not too high; not too low)? | | 1 |  |
|  | Is flocculation time sufficient (at least 15 minutes)? | | 1 |  |
|  | Can the flocculation channels or tanks be cleaned easily? | | 1 |  |
| Sedimentation | Is floc break-up prevented in the inlet to the sedimentation tank? | | 1 |  |
|  | Is the upflow velocity in the sedimentation tank sufficiently low? | | 1 |  |
|  | Is the weir overflow rate sufficiently low? | | 1 |  |
|  | Can the sedimentation tank(s) be desludged easily and effectively? | | 1 |  |
| Filtration | Is filtration rate sufficiently low to ensure efficient filtration? | | 1 |  |
|  | Is the filtration depth and filter media suitable for the type of raw water? | | 1 |  |
|  | Is the underdrain system acceptable to ensure sustainable filtration? | | 1 |  |
|  | Is the backwashing system suitable to ensure effective cleaning of sand filters? | | 1 |  |
| Disinfection | Can chlorine (or other disinfectant) be dosed at sufficient levels for the whole range of inflow rates and chlorine demands of the raw water (i.e. are dosing pumps sized correctly)? | | 1 |  |
|  | Are monitoring systems in place to ensure timeous changing of chlorine cylinders or to replenish disinfectant make-up solutions? | | 1 |  |
| Dosing systems | Have dosage systems (coagulation; pH adjustment; oxidation; disinfection; stabilisation) been installed to ensure easy maintenance and trouble-free operation? | | 1 |  |
| Maximum possible score for ***Design Aspects*** | | | **17** | |
| Total score attained for ***Design Aspects*** | | | **17** | |
| Weight for ***Design Aspects*** | | | **0.1** | |
| Total weighted score for ***Design Aspects*** | | | **0.1** | |

| **Table 1.3**  **TECHNICAL COMPLIANCE SCORING:** | | | |
| --- | --- | --- | --- |
| **Criterion** | | **Yes =1** | **No = 0** |
| **1. TREATMENT PLANT MEASUREMENTS** | | | |
| **1B: Operation Monitoring Practices** | | | |
| Coagulation and Flocculation | Do the process controllers know how much they are dosing (even qualitatively)? | **1** |  |
|  | Can the process controllers adjust the dosage rates and monitor how much is dosed? | **1** |  |
|  | Do they have a programme for monitoring floc formation? |  | **0** |
| Sedimentation | Is the floc blanket observed and dislodging practices based thereon? | **1** |  |
|  | Is the overflow weir kept clean? | **1** |  |
|  | Is the flow of more than one sedimentation tank distributed evenly? | **1** |  |
| Filtration | Is backwashing done properly (at the right time and according to the correct procedure)? | **1** |  |
|  | Is the quality of the filtrate monitored on a regular basis? |  | **0** |
|  | Is excessive head loss development or turbidity breakthrough monitored? |  | **0** |
| Disinfection | Is the chlorine dosed according to previously determined chlorine demand and/or by maintaining acceptable chlorine residual in the final water? |  | **0** |
|  | Is the chlorine residual measured correctly and at the suggested frequency? |  | **0** |
| Stabilisation | Is the stability of the final water determined? |  | **0** |
|  | Is the process controller familiar with the reason for stabilisation and how it can be affected and controlled? |  | **0** |
| **Maximum possible score for *Operation Monitoring Practices*** | | **13** | |
| **Total score attained for *Operation Monitoring Practices*** | | **6** | |
| **Weight for *Operation Monitoring Practices*** | | **0.2** | |
| **Total weighted score for *Operation Monitoring Practices*** | | **0.092** | |

| **Table 1.4**  **TECHNICAL COMPLIANCE SCORING:** | | | |
| --- | --- | --- | --- |
| **Criterion** | | **Yes =1** | **No = 0** |
| **1. TREATMENT PLANT MEASUREMENTS** | | | |
| **1C: Compliance Monitoring Practices** | | | |
| Final water quality | Is the free chlorine residual sufficient? |  | **0** |
|  | Is the turbidity less than 1.0 NTU? | **1** |  |
|  | Is the pH between 6.0 and 9.0? | **1** |  |
|  | Is the colour less than 10 mg/L as Pt? |  | **N/A** |
|  | Is the final water free of any coliform indicator organisms? | **1** |  |
|  | Is the electrical conductivity less than 70 mS/m? |  | **N/A** |
|  | Is the iron level less than 0.2 mg/L as Fe? |  | **N/A** |
|  | Is the aluminium level less than 0.2 mg/L as Al? |  | **N/A** |
| Maximum possible score for ***Compliance Monitoring Practices*** | | **4** | |
| Total score attained for ***Compliance Monitoring Practices*** | | **3** | |
| Weight for ***Compliance Monitoring Practices*** | | **0.3** | |
| Total weighted score for ***Compliance Monitoring Practices*** | | **0.225** | |

| **Table 1.5**  **TECHNICAL COMPLIANCE SCORING:** | | | |
| --- | --- | --- | --- |
| **Criterion** | | **Yes =1** | **No = 0** |
| **1. TREATMENT PLANT MEASUREMENTS** | | | |
| **1D: Plant Monitoring Practices** | | | |
| Plant Infrastructure and Equipment | Are all the pumps in working order? | **1** |  |
|  | Can all the valves open and close properly? | **1** |  |
|  | Are all leaks repaired? | **1** |  |
|  | Are all blockages in pipes cleared? | **1** |  |
|  | Are all unit treatment processes and equipment accessible? | **1** |  |
| Maximum possible score for ***Plant Monitoring Practices*** | | **5** | |
| Total score attained for ***Plant Monitoring Practices*** | | **5** | |
| Weight for ***Plant Monitoring Practices*** | | **0.1** | |
| Total weighted score for ***Plant Monitoring Practices*** | | **0.1** | |

| **Table 1.6**  **TECHNICAL COMPLIANCE SCORING:** | | | |
| --- | --- | --- | --- |
| **Criterion** | | **Yes =1** | **No = 0** |
| **1. TREATMENT PLANT MEASUREMENTS** | | | |
| 1E: Maintenance Practices | | | |
| Overall Treatment Plant | Is back-up service available? | **1** |  |
|  | Is there opportunity for training of the plant personnel in using the sophisticated equipment? | **1** |  |
|  | Are spare-parts readily available? | **1** |  |
|  | Can spare parts be replaced quickly? | **1** |  |
|  | Are spare parts affordable by the municipality? | **1** |  |
|  | Is back-up service readily available? | **1** |  |
|  | Can service providers be on site at short notice? | **1** |  |
|  | Is this service reliable? | **1** |  |
|  | Can the community do certain parts of the maintenance themselves? |  | **N/A** |
|  | Are there adequate communication facilities between the plant and management, authorities, service providers, suppliers and consultants? | **1** |  |
|  | Can this be readily improved? | **1** |  |
|  | Can the plant personnel or persons in the community perform their own maintenance (or parts thereof)? |  | **N/A** |
|  | Can they be trained and are such training facilities available? | **1** |  |
|  | Is there good access to the plant by motor vehicles and delivery trucks? | **1** |  |
|  | Can the existing access routes be improved with existing funds? | **1** |  |
|  | Are funds available? | **1** |  |
|  | Is there possibility of alternative sources of funding for maintenance? | **1** |  |
| Maximum possible score for ***Maintenance Practices*** | | **18** | |
| Total score attained for ***Maintenance Practices*** | | **15** | |
| Weight for ***Maintenance Practices*** | | **0.2** | |
| Total weighted score for ***Maintenance Practices*** | | **0.167** | |

| **Table 1.7**  **TECHNICAL COMPLIANCE SCORING:** | | | |
| --- | --- | --- | --- |
| **Criterion** | | **Yes =1** | **No = 0** |
| **1. TREATMENT PLANT MEASUREMENTS** | | | |
| **1F: Risk Management Practices** | | | |
| Source to Tap | Is there a risk management system in place? |  | **0** |
|  | Are risk reduction options drawn up and implemented based on the risk assessment? |  | **0** |
|  | Are the risk assessments carried out on a regular basis? |  | **0** |
| Maximum possible score for ***Risk Management Practices*** | | **3** | |
| Total score attained for ***Risk Management Practices*** | | **0** | |
| Weight for ***Risk Management Practices*** | | **0.1** | |
| Total weighted score for ***Risk Management Practices*** | | **0.0** | |

| **TABLE 1.8**  **TECHNICAL COMPLIANCE SCORING:**  **Total Weighted Scoring for Malamulele treatment plant 68.4 %** | |
| --- | --- |
| **Compliance Criterion** | **Weighted Score** |
| **1. TREATMENT PLANT MEASUREMENTS** | |
| 1A: Design Aspects | **0.1** |
| 1B: Operation Monitoring Practices | **0.092** |
| 1C: Compliance (Final Water Quality) Monitoring Practices | **0.225** |
| 1D: Plant Monitoring Practices | **0.1** |
| 1E: Maintenance Practices | **0.167** |
| 1F: Risk Management Practices | **0.0** |
| TOTAL WEIGHTED SCORE | **1.0** |

**3.5 Technical compliance rating of small water treatment plants**

Based on the Technical Compliance Scoring system used above and the final total weighted score calculated for the specific water treatment plant, the plant can then be rated according to the following Technical Compliance Rating:

| **TABLE 1.9**  **TECHNICAL COMPLIANCE RATING** | |
| --- | --- |
| **Total Weighted Score** | **Rating Description** |
| **0 – 50** | Class 3 Compliance: Total non-compliance; serious and immediate intervention required (TAC) |
| **50 – 90** | Class 2 Compliance: Serious challenges requiring attention and improvement |
| **90 – 100** | Class 1 Compliance: Acceptable compliance |

The following chapter focuses on the corrective actions for non-technical compliance that can be recorded in a drinking water supply.

**18 august 2009**

1. **Vondo Water Scheme**

| **Table 2.1**  **TECHNICAL COMPLIANCE SCORING** | |
| --- | --- |
| **Criterion** | **Weight** |
| **1. TREATMENT PLANT MEASUREMENTS** | |
| 1A: Design Aspects | **0.1** |
| 1B: Operation Monitoring Practices | **0.2** |
| 1C: Compliance (Final Water Quality) Monitoring Practices | **0.3** |
| 1D: Plant Monitoring Practices | **0.1** |
| 1E: Maintenance Practices | **0.2** |
| 1F: Risk Management Practices | **0.1** |
| **TOTAL** | **1.0** |

| **Table 2.2**  **TECHNICAL COMPLIANCE SCORING:** | | | |
| --- | --- | --- | --- |
| **Criterion** | | **Yes =1** | **No = 0** |
| **1. TREATMENT PLANT MEASUREMENTS** | | | |
| **1A: Design Aspects** | | | |
| Rapid Mixing | Is the mixing intensity sufficient? | 1 |  |
|  | Is the mixing time sufficient? | 1 |  |
|  | Are chemicals dosed at the correct points? | 1 |  |
| Flocculation | Is flocculation intensity at correct levels (not too high; not too low)? |  | N/A |
|  | Is flocculation time sufficient (at least 15 minutes)? |  | N/A |
|  | Can the flocculation channels or tanks be cleaned easily? |  | N/A |
| Sedimentation | Is floc break-up prevented in the inlet to the sedimentation tank? |  | N/A |
|  | Is the upflow velocity in the sedimentation tank sufficiently low? |  | N/A |
|  | Is the weir overflow rate sufficiently low? |  | N/A |
|  | Can the sedimentation tank(s) be desludged easily and effectively? |  | N/A |
| Filtration | Is filtration rate sufficiently low to ensure efficient filtration? |  | N/A |
|  | Is the filtration depth and filter media suitable for the type of raw water? |  | N/A |
|  | Is the underdrain system acceptable to ensure sustainable filtration? | 1 |  |
|  | Is the backwashing system suitable to ensure effective cleaning of sand filters? | 1 |  |
| Disinfection | Can chlorine (or other disinfectant) be dosed at sufficient levels for the whole range of inflow rates and chlorine demands of the raw water (i.e. are dosing pumps sized correctly)? | 1 |  |
|  | Are monitoring systems in place to ensure timeous changing of chlorine cylinders or to replenish disinfectant make-up solutions? |  | 0 |
| Dosing systems | Have dosage systems (coagulation; pH adjustment; oxidation; disinfection; stabilisation) been installed to ensure easy maintenance and trouble-free operation? | 1 |  |
| Maximum possible score for ***Design Aspects*** | | **17** | |
| Total score attained for ***Design Aspects*** | | **7** | |
| Weight for ***Design Aspects*** | | **0.1** | |
| Total weighted score for ***Design Aspects*** | | **0.041** | |

| **Table 2.3**  **TECHNICAL COMPLIANCE SCORING:** | | | |
| --- | --- | --- | --- |
| **Criterion** | | **Yes =1** | **No = 0** |
| **1. TREATMENT PLANT MEASUREMENTS** | | | |
| **1B: Operation Monitoring Practices** | | | |
| Coagulation and Flocculation | Do the process controllers know how much they are dosing (even qualitatively)? | **1** |  |
|  | Can the process controllers adjust the dosage rates and monitor how much is dosed? | **1** |  |
|  | Do they have a programme for monitoring floc formation? |  | **N/A** |
| Sedimentation | Is the floc blanket observed and dislodging practices based thereon? |  | **N/A** |
|  | Is the overflow weir kept clean? |  | **N/A** |
|  | Is the flow of more than one sedimentation tank distributed evenly? |  | **N/A** |
| Filtration | Is backwashing done properly (at the right time and according to the correct procedure)? | **1** |  |
|  | Is the quality of the filtrate monitored on a regular basis? |  | **0** |
|  | Is excessive head loss development or turbidity breakthrough monitored? |  | **N/A** |
| Disinfection | Is the chlorine dosed according to previously determined chlorine demand and/or by maintaining acceptable chlorine residual in the final water? | **1** |  |
|  | Is the chlorine residual measured correctly and at the suggested frequency? | **1** |  |
| Stabilisation | Is the stability of the final water determined? | **1** |  |
|  | Is the process controller familiar with the reason for stabilisation and how it can be affected and controlled? | **1** |  |
| **Maximum possible score for *Operation Monitoring Practices*** | | **8** | |
| **Total score attained for *Operation Monitoring Practices*** | | **7** | |
| **Weight for *Operation Monitoring Practices*** | | **0.2** | |
| **Total weighted score for *Operation Monitoring Practices*** | | **0.175** | |

| **Table 2.4**  **TECHNICAL COMPLIANCE SCORING:** | | | |
| --- | --- | --- | --- |
| **Criterion** | | **Yes =1** | **No = 0** |
| **1. TREATMENT PLANT MEASUREMENTS** | | | |
| **1C: Compliance Monitoring Practices** | | | |
| Final water quality | Is the free chlorine residual sufficient? |  | **0** |
|  | Is the turbidity less than 1.0 NTU? |  | **0** |
|  | Is the pH between 6.0 and 9.0? | **1** |  |
|  | Is the colour less than 10 mg/L as Pt? |  | **N/A** |
|  | Is the final water free of any coliform indicator organisms? |  | **0** |
|  | Is the electrical conductivity less than 70 mS/m? |  | **N/A** |
|  | Is the iron level less than 0.2 mg/L as Fe? |  | **N/A** |
|  | Is the aluminium level less than 0.2 mg/L as Al? |  | **N/A** |
| Maximum possible score for ***Compliance Monitoring Practices*** | | **4** | |
| Total score attained for ***Compliance Monitoring Practices*** | | **1** | |
| Weight for ***Compliance Monitoring Practices*** | | **0.3** | |
| Total weighted score for ***Compliance Monitoring Practices*** | | **0.075** | |

| **Table 2.5**  **TECHNICAL COMPLIANCE SCORING:** | | | |
| --- | --- | --- | --- |
| **Criterion** | | **Yes =1** | **No = 0** |
| **1. TREATMENT PLANT MEASUREMENTS** | | | |
| **1D: Plant Monitoring Practices** | | | |
| Plant Infrastructure and Equipment | Are all the pumps in working order? | **1** |  |
|  | Can all the valves open and close properly? | **1** |  |
|  | Are all leaks repaired? | **1** |  |
|  | Are all blockages in pipes cleared? | **1** |  |
|  | Are all unit treatment processes and equipment accessible? | **1** |  |
| Maximum possible score for ***Plant Monitoring Practices*** | | **5** | |
| Total score attained for ***Plant Monitoring Practices*** | | **5** | |
| Weight for ***Plant Monitoring Practices*** | | **0.1** | |
| Total weighted score for ***Plant Monitoring Practices*** | | **0.1** | |

| **Table 2.6**  **TECHNICAL COMPLIANCE SCORING:** | | | |
| --- | --- | --- | --- |
| **Criterion** | | **Yes =1** | **No = 0** |
| **1. TREATMENT PLANT MEASUREMENTS** | | | |
| 1E: Maintenance Practices | | | |
| Overall Treatment Plant | Is back-up service available? | **1** |  |
|  | Is there opportunity for training of the plant personnel in using the sophisticated equipment? | **1** |  |
|  | Are spare-parts readily available? | **1** |  |
|  | Can spare parts be replaced quickly? | **1** |  |
|  | Are spare parts affordable by the municipality? | **1** |  |
|  | Is back-up service readily available? | **1** |  |
|  | Can service providers be on site at short notice? | **1** |  |
|  | Is this service reliable? | **1** |  |
|  | Can the community do certain parts of the maintenance themselves? |  | **N/A** |
|  | Are there adequate communication facilities between the plant and management, authorities, service providers, suppliers and consultants? | **1** |  |
|  | Can this be readily improved? | **1** |  |
|  | Can the plant personnel or persons in the community perform their own maintenance (or parts thereof)? |  | **N/A** |
|  | Can they be trained and are such training facilities available? | **1** |  |
|  | Is there good access to the plant by motor vehicles and delivery trucks? | **1** |  |
|  | Can the existing access routes be improved with existing funds? | **1** |  |
|  | Are funds available? | **1** |  |
|  | Is there possibility of alternative sources of funding for maintenance? | **1** |  |
| Maximum possible score for ***Maintenance Practices*** | | **18** | |
| Total score attained for ***Maintenance Practices*** | | **15** | |
| Weight for ***Maintenance Practices*** | | **0.2** | |
| Total weighted score for ***Maintenance Practices*** | | **0.167** | |

| **Table 2.7**  **TECHNICAL COMPLIANCE SCORING:** | | | |
| --- | --- | --- | --- |
| **Criterion** | | **Yes =1** | **No = 0** |
| **1. TREATMENT PLANT MEASUREMENTS** | | | |
| **1F: Risk Management Practices** | | | |
| Source to Tap | Is there a risk management system in place? |  | **0** |
|  | Are risk reduction options drawn up and implemented based on the risk assessment? |  | **0** |
|  | Are the risk assessments carried out on a regular basis? |  | **0** |
| Maximum possible score for ***Risk Management Practices*** | | **3** | |
| Total score attained for ***Risk Management Practices*** | | **0** | |
| Weight for ***Risk Management Practices*** | | **0.1** | |
| Total weighted score for ***Risk Management Practices*** | | **0** | |

| **TABLE 2.8**  **TECHNICAL COMPLIANCE SCORING:**  **Total Weighted Scoring for Vondo Water treatment plant 55.8 %** | |
| --- | --- |
| **Compliance Criterion** | **Weighted Score** |
| **1. TREATMENT PLANT MEASUREMENTS** | |
| 1A: Design Aspects | **0.041** |
| 1B: Operation Monitoring Practices | **0.175** |
| 1C: Compliance (Final Water Quality) Monitoring Practices | **0.075** |
| 1D: Plant Monitoring Practices | **0.1** |
| 1E: Maintenance Practices | **0.167** |
| 1F: Risk Management Practices | **0.0** |
| TOTAL WEIGHTED SCORE | **1.0** |

**3.5 Technical compliance rating of small water treatment plants**

Based on the Technical Compliance Scoring system used above and the final total weighted score calculated for the specific water treatment plant, the plant can then be rated according to the following Technical Compliance Rating:

| **TABLE 2.9**  **TECHNICAL COMPLIANCE RATING** | |
| --- | --- |
| **Total Weighted Score** | **Rating Description** |
| **0 – 50** | Class 3 Compliance: Total non-compliance; serious and immediate intervention required (TAC) |
| **50 – 90** | Class 2 Compliance: Serious challenges requiring attention and improvement |
| **90 – 100** | Class 1 Compliance: Acceptable compliance |

The following chapter focuses on the corrective actions for non-technical compliance that can be recorded in a drinking water supply.

1. **august 2009**
2. **Mutshedzi Water treatment plant**

| **Table 3.1**  **TECHNICAL COMPLIANCE SCORING** | |
| --- | --- |
| **Criterion** | **Weight** |
| **1. TREATMENT PLANT MEASUREMENTS** | |
| 1A: Design Aspects | **0.1** |
| 1B: Operation Monitoring Practices | **0.2** |
| 1C: Compliance (Final Water Quality) Monitoring Practices | **0.3** |
| 1D: Plant Monitoring Practices | **0.1** |
| 1E: Maintenance Practices | **0.2** |
| 1F: Risk Management Practices | **0.1** |
| **TOTAL** | **1.0** |

| **Table 3.2**  **TECHNICAL COMPLIANCE SCORING:** | | | |
| --- | --- | --- | --- |
| **Criterion** | | **Yes =1** | **No = 0** |
| **1. TREATMENT PLANT MEASUREMENTS** | | | |
| **1A: Design Aspects** | | | |
| Rapid Mixing | Is the mixing intensity sufficient? | 1 |  |
|  | Is the mixing time sufficient? | 1 |  |
|  | Are chemicals dosed at the correct points? | 1 |  |
| Flocculation | Is flocculation intensity at correct levels (not too high; not too low)? | 1 |  |
|  | Is flocculation time sufficient (at least 15 minutes)? | 1 |  |
|  | Can the flocculation channels or tanks be cleaned easily? | 1 |  |
| Sedimentation | Is floc break-up prevented in the inlet to the sedimentation tank? | 1 |  |
|  | Is the upflow velocity in the sedimentation tank sufficiently low? | 1 |  |
|  | Is the weir overflow rate sufficiently low? | 1 |  |
|  | Can the sedimentation tank(s) be desludged easily and effectively? | 1 |  |
| Filtration | Is filtration rate sufficiently low to ensure efficient filtration? | 1 |  |
|  | Is the filtration depth and filter media suitable for the type of raw water? | 1 |  |
|  | Is the underdrain system acceptable to ensure sustainable filtration? | 1 |  |
|  | Is the backwashing system suitable to ensure effective cleaning of sand filters? | 1 |  |
| Disinfection | Can chlorine (or other disinfectant) be dosed at sufficient levels for the whole range of inflow rates and chlorine demands of the raw water (i.e. are dosing pumps sized correctly)? | 1 |  |
|  | Are monitoring systems in place to ensure timeous changing of chlorine cylinders or to replenish disinfectant make-up solutions? | 1 |  |
| Dosing systems | Have dosage systems (coagulation; pH adjustment; oxidation; disinfection; stabilisation) been installed to ensure easy maintenance and trouble-free operation? | 1 |  |
| Maximum possible score for ***Design Aspects*** | | **17** | |
| Total score attained for ***Design Aspects*** | | **17** | |
| Weight for ***Design Aspects*** | | **0.1** | |
| Total weighted score for ***Design Aspects*** | | **0.1** | |

| **Table 3.3**  **TECHNICAL COMPLIANCE SCORING:** | | | |
| --- | --- | --- | --- |
| **Criterion** | | **Yes =1** | **No = 0** |
| **1. TREATMENT PLANT MEASUREMENTS** | | | |
| **1B: Operation Monitoring Practices** | | | |
| Coagulation and Flocculation | Do the process controllers know how much they are dosing (even qualitatively)? | **1** |  |
|  | Can the process controllers adjust the dosage rates and monitor how much is dosed? | **1** |  |
|  | Do they have a programme for monitoring floc formation? |  | **0** |
| Sedimentation | Is the floc blanket observed and dislodging practices based thereon? | **1** |  |
|  | Is the overflow weir kept clean? | **1** |  |
|  | Is the flow of more than one sedimentation tank distributed evenly? | **1** |  |
| Filtration | Is backwashing done properly (at the right time and according to the correct procedure)? | **1** |  |
|  | Is the quality of the filtrate monitored on a regular basis? |  | **0** |
|  | Is excessive head loss development or turbidity breakthrough monitored? |  | **0** |
| Disinfection | Is the chlorine dosed according to previously determined chlorine demand and/or by maintaining acceptable chlorine residual in the final water? |  | **0** |
|  | Is the chlorine residual measured correctly and at the suggested frequency? |  | **0** |
| Stabilisation | Is the stability of the final water determined? |  | **0** |
|  | Is the process controller familiar with the reason for stabilisation and how it can be affected and controlled? |  | **0** |
| **Maximum possible score for *Operation Monitoring Practices*** | | **13** | |
| **Total score attained for *Operation Monitoring Practices*** | | **6** | |
| **Weight for *Operation Monitoring Practices*** | | **0.2** | |
| **Total weighted score for *Operation Monitoring Practices*** | | **0.0923** | |

| **Table 3.4**  **TECHNICAL COMPLIANCE SCORING:** | | | |
| --- | --- | --- | --- |
| **Criterion** | | **Yes =1** | **No = 0** |
| **1. TREATMENT PLANT MEASUREMENTS** | | | |
| **1C: Compliance Monitoring Practices** | | | |
| Final water quality | Is the free chlorine residual sufficient? | **1** |  |
|  | Is the turbidity less than 1.0 NTU? | **1** |  |
|  | Is the pH between 6.0 and 9.0? | **1** |  |
|  | Is the colour less than 10 mg/L as Pt? |  | **N/A** |
|  | Is the final water free of any coliform indicator organisms? |  | **0** |
|  | Is the electrical conductivity less than 70 mS/m? |  | **N/A** |
|  | Is the iron level less than 0.2 mg/L as Fe? |  | **N/A** |
|  | Is the aluminium level less than 0.2 mg/L as Al? |  | **N/A** |
| Maximum possible score for ***Compliance Monitoring Practices*** | | **4** | |
| Total score attained for ***Compliance Monitoring Practices*** | | **3** | |
| Weight for ***Compliance Monitoring Practices*** | | **0.3** | |
| Total weighted score for ***Compliance Monitoring Practices*** | | **0.225** | |

| **Table 3.5**  **TECHNICAL COMPLIANCE SCORING:** | | | |
| --- | --- | --- | --- |
| **Criterion** | | **Yes =1** | **No = 0** |
| **1. TREATMENT PLANT MEASUREMENTS** | | | |
| **1D: Plant Monitoring Practices** | | | |
| Plant Infrastructure and Equipment | Are all the pumps in working order? | **1** |  |
|  | Can all the valves open and close properly? | **1** |  |
|  | Are all leaks repaired? | **1** |  |
|  | Are all blockages in pipes cleared? | **1** |  |
|  | Are all unit treatment processes and equipment accessible? | **1** |  |
| Maximum possible score for ***Plant Monitoring Practices*** | | **5** | |
| Total score attained for ***Plant Monitoring Practices*** | | **5** | |
| Weight for ***Plant Monitoring Practices*** | | **0.1** | |
| Total weighted score for ***Plant Monitoring Practices*** | | **0.1** | |

| **Table 3.6**  **TECHNICAL COMPLIANCE SCORING:** | | | |
| --- | --- | --- | --- |
| **Criterion** | | **Yes =1** | **No = 0** |
| **1. TREATMENT PLANT MEASUREMENTS** | | | |
| 1E: Maintenance Practices | | | |
| Overall Treatment Plant | Is back-up service available? | **1** |  |
|  | Is there opportunity for training of the plant personnel in using the sophisticated equipment? | **1** |  |
|  | Are spare-parts readily available? | **1** |  |
|  | Can spare parts be replaced quickly? | **1** |  |
|  | Are spare parts affordable by the municipality? | **1** |  |
|  | Is back-up service readily available? | **1** |  |
|  | Can service providers be on site at short notice? | **1** |  |
|  | Is this service reliable? | **1** |  |
|  | Can the community do certain parts of the maintenance themselves? |  | **N/A** |
|  | Are there adequate communication facilities between the plant and management, authorities, service providers, suppliers and consultants? | **1** |  |
|  | Can this be readily improved? | **1** |  |
|  | Can the plant personnel or persons in the community perform their own maintenance (or parts thereof)? |  | **N/A** |
|  | Can they be trained and are such training facilities available? | **1** |  |
|  | Is there good access to the plant by motor vehicles and delivery trucks? | **1** |  |
|  | Can the existing access routes be improved with existing funds? | **1** |  |
|  | Are funds available? | **1** |  |
|  | Is there possibility of alternative sources of funding for maintenance? | **1** |  |
| Maximum possible score for ***Maintenance Practices*** | | **18** | |
| Total score attained for ***Maintenance Practices*** | | **15** | |
| Weight for ***Maintenance Practices*** | | **0.2** | |
| Total weighted score for ***Maintenance Practices*** | | **0.167** | |

| **Table 3.7**  **TECHNICAL COMPLIANCE SCORING:** | | | |
| --- | --- | --- | --- |
| **Criterion** | | **Yes =1** | **No = 0** |
| **1. TREATMENT PLANT MEASUREMENTS** | | | |
| **1F: Risk Management Practices** | | | |
| Source to Tap | Is there a risk management system in place? |  | **0** |
|  | Are risk reduction options drawn up and implemented based on the risk assessment? |  | **0** |
|  | Are the risk assessments carried out on a regular basis? |  | **0** |
| Maximum possible score for ***Risk Management Practices*** | | **3** | |
| Total score attained for ***Risk Management Practices*** | | **0** | |
| Weight for ***Risk Management Practices*** | | **0.1** | |
| Total weighted score for ***Risk Management Practices*** | | **0.0** | |

| **TABLE 3.8**  **TECHNICAL COMPLIANCE SCORING:**  **Total Weighted Scoring for Mutshedzi Water Treatment Plant 55.8 %** | |
| --- | --- |
| **Compliance Criterion** | **Weighted Score** |
| **1. TREATMENT PLANT MEASUREMENTS** | |
| 1A: Design Aspects | **0.041** |
| 1B: Operation Monitoring Practices | **0.175** |
| 1C: Compliance (Final Water Quality) Monitoring Practices | **0.075** |
| 1D: Plant Monitoring Practices | **0.1** |
| 1E: Maintenance Practices | **0.167** |
| 1F: Risk Management Practices | **0.00** |
| TOTAL WEIGHTED SCORE | **1.0** |

**3.5 Technical compliance rating of small water treatment plants**

Based on the Technical Compliance Scoring system used above and the final total weighted score calculated for the specific water treatment plant, the plant can then be rated according to the following Technical Compliance Rating:

| **TABLE 3.9**  **TECHNICAL COMPLIANCE RATING** | |
| --- | --- |
| **Total Weighted Score** | **Rating Description** |
| **0 – 50** | Class 3 Compliance: Total non-compliance; serious and immediate intervention required (TAC) |
| **50 – 90** | Class 2 Compliance: Serious challenges requiring attention and improvement |
| **90 – 100** | Class 1 Compliance: Acceptable compliance |

The following chapter focuses on the corrective actions for non-technical compliance that can be recorded in a drinking water supply.

**19 august 2009**

1. **Mutale regional Water treatment plant**

| **Table 4.1**  **TECHNICAL COMPLIANCE SCORING** | |
| --- | --- |
| **Criterion** | **Weight** |
| **1. TREATMENT PLANT MEASUREMENTS** | |
| 1A: Design Aspects | **0.1** |
| 1B: Operation Monitoring Practices | **0.2** |
| 1C: Compliance (Final Water Quality) Monitoring Practices | **0.3** |
| 1D: Plant Monitoring Practices | **0.1** |
| 1E: Maintenance Practices | **0.2** |
| 1F: Risk Management Practices | **0.1** |
| **TOTAL** | **1.0** |

| **Table 4.2**  **TECHNICAL COMPLIANCE SCORING:** | | | |
| --- | --- | --- | --- |
| **Criterion** | | **Yes =1** | **No = 0** |
| **1. TREATMENT PLANT MEASUREMENTS** | | | |
| **1A: Design Aspects** | | | |
| Rapid Mixing | Is the mixing intensity sufficient? | 1 |  |
|  | Is the mixing time sufficient? | 1 |  |
|  | Are chemicals dosed at the correct points? | 1 |  |
| Flocculation | Is flocculation intensity at correct levels (not too high; not too low)? | 1 |  |
|  | Is flocculation time sufficient (at least 15 minutes)? | 1 |  |
|  | Can the flocculation channels or tanks be cleaned easily? | 1 |  |
| Sedimentation | Is floc break-up prevented in the inlet to the sedimentation tank? | 1 |  |
|  | Is the upflow velocity in the sedimentation tank sufficiently low? | 1 |  |
|  | Is the weir overflow rate sufficiently low? | 1 |  |
|  | Can the sedimentation tank(s) be desludged easily and effectively? | 1 |  |
| Filtration | Is filtration rate sufficiently low to ensure efficient filtration? | 1 |  |
|  | Is the filtration depth and filter media suitable for the type of raw water? | 1 |  |
|  | Is the underdrain system acceptable to ensure sustainable filtration? | 1 |  |
|  | Is the backwashing system suitable to ensure effective cleaning of sand filters? | 1 |  |
| Disinfection | Can chlorine (or other disinfectant) be dosed at sufficient levels for the whole range of inflow rates and chlorine demands of the raw water (i.e. are dosing pumps sized correctly)? | 1 |  |
|  | Are monitoring systems in place to ensure timeous changing of chlorine cylinders or to replenish disinfectant make-up solutions? | 1 |  |
| Dosing systems | Have dosage systems (coagulation; pH adjustment; oxidation; disinfection; stabilisation) been installed to ensure easy maintenance and trouble-free operation? |  | 0 |
| Maximum possible score for ***Design Aspects*** | | **17** | |
| Total score attained for ***Design Aspects*** | | **16** | |
| Weight for ***Design Aspects*** | | **0.1** | |
| Total weighted score for ***Design Aspects*** | | **0.094** | |

| **Table 4.3**  **TECHNICAL COMPLIANCE SCORING:** | | | |
| --- | --- | --- | --- |
| **Criterion** | | **Yes =1** | **No = 0** |
| **1. TREATMENT PLANT MEASUREMENTS** | | | |
| **1B: Operation Monitoring Practices** | | | |
| Coagulation and Flocculation | Do the process controllers know how much they are dosing (even qualitatively)? | **1** |  |
|  | Can the process controllers adjust the dosage rates and monitor how much is dosed? | **1** |  |
|  | Do they have a programme for monitoring floc formation? |  | **0** |
| Sedimentation | Is the floc blanket observed and dislodging practices based thereon? | **1** |  |
|  | Is the overflow weir kept clean? | **1** |  |
|  | Is the flow of more than one sedimentation tank distributed evenly? | **1** |  |
| Filtration | Is backwashing done properly (at the right time and according to the correct procedure)? | **1** |  |
|  | Is the quality of the filtrate monitored on a regular basis? |  | **0** |
|  | Is excessive head loss development or turbidity breakthrough monitored? |  | **0** |
| Disinfection | Is the chlorine dosed according to previously determined chlorine demand and/or by maintaining acceptable chlorine residual in the final water? |  | **0** |
|  | Is the chlorine residual measured correctly and at the suggested frequency? |  | **0** |
| Stabilisation | Is the stability of the final water determined? |  | **0** |
|  | Is the process controller familiar with the reason for stabilisation and how it can be affected and controlled? |  | **0** |
| **Maximum possible score for *Operation Monitoring Practices*** | | **13** | |
| **Total score attained for *Operation Monitoring Practices*** | | **6** | |
| **Weight for *Operation Monitoring Practices*** | | **0.2** | |
| **Total weighted score for *Operation Monitoring Practices*** | | **0.092** | |

| **Table 4.4**  **TECHNICAL COMPLIANCE SCORING:** | | | |
| --- | --- | --- | --- |
| **Criterion** | | **Yes =1** | **No = 0** |
| **1. TREATMENT PLANT MEASUREMENTS** | | | |
| **1C: Compliance Monitoring Practices** | | | |
| Final water quality | Is the free chlorine residual sufficient? | **1** |  |
|  | Is the turbidity less than 1.0 NTU? |  | **0** |
|  | Is the pH between 6.0 and 9.0? | **1** |  |
|  | Is the colour less than 10 mg/L as Pt? |  | **N/A** |
|  | Is the final water free of any coliform indicator organisms? |  | **0** |
|  | Is the electrical conductivity less than 70 mS/m? |  | **N/A** |
|  | Is the iron level less than 0.2 mg/L as Fe? |  | **N/A** |
|  | Is the aluminium level less than 0.2 mg/L as Al? |  | **N/A** |
| Maximum possible score for ***Compliance Monitoring Practices*** | | **4** | |
| Total score attained for ***Compliance Monitoring Practices*** | | **2** | |
| Weight for ***Compliance Monitoring Practices*** | | **0.3** | |
| Total weighted score for ***Compliance Monitoring Practices*** | | **0.15** | |

| **Table 4.5**  **TECHNICAL COMPLIANCE SCORING:** | | | |
| --- | --- | --- | --- |
| **Criterion** | | **Yes =1** | **No = 0** |
| **1. TREATMENT PLANT MEASUREMENTS** | | | |
| **1D: Plant Monitoring Practices** | | | |
| Plant Infrastructure and Equipment | Are all the pumps in working order? | **1** |  |
|  | Can all the valves open and close properly? | **1** |  |
|  | Are all leaks repaired? |  | **0** |
|  | Are all blockages in pipes cleared? | **1** |  |
|  | Are all unit treatment processes and equipment accessible? | **1** |  |
| Maximum possible score for ***Plant Monitoring Practices*** | | **5** | |
| Total score attained for ***Plant Monitoring Practices*** | | **5** | |
| Weight for ***Plant Monitoring Practices*** | | **0.1** | |
| Total weighted score for ***Plant Monitoring Practices*** | | **0.1** | |

| **Table 4.6**  **TECHNICAL COMPLIANCE SCORING:** | | | |
| --- | --- | --- | --- |
| **Criterion** | | **Yes =1** | **No = 0** |
| **1. TREATMENT PLANT MEASUREMENTS** | | | |
| 1E: Maintenance Practices | | | |
| Overall Treatment Plant | Is back-up service available? | **1** |  |
|  | Is there opportunity for training of the plant personnel in using the sophisticated equipment? | **1** |  |
|  | Are spare-parts readily available? | **1** |  |
|  | Can spare parts be replaced quickly? | **1** |  |
|  | Are spare parts affordable by the municipality? | **1** |  |
|  | Is back-up service readily available? | **1** |  |
|  | Can service providers be on site at short notice? | **1** |  |
|  | Is this service reliable? | **1** |  |
|  | Can the community do certain parts of the maintenance themselves? |  | **N/A** |
|  | Are there adequate communication facilities between the plant and management, authorities, service providers, suppliers and consultants? | **1** |  |
|  | Can this be readily improved? | **1** |  |
|  | Can the plant personnel or persons in the community perform their own maintenance (or parts thereof)? |  | **N/A** |
|  | Can they be trained and are such training facilities available? | **1** |  |
|  | Is there good access to the plant by motor vehicles and delivery trucks? | **1** |  |
|  | Can the existing access routes be improved with existing funds? | **1** |  |
|  | Are funds available? | **1** |  |
|  | Is there possibility of alternative sources of funding for maintenance? | **1** |  |
| Maximum possible score for ***Maintenance Practices*** | | **18** | |
| Total score attained for ***Maintenance Practices*** | | **15** | |
| Weight for ***Maintenance Practices*** | | **0.2** | |
| Total weighted score for ***Maintenance Practices*** | | **0.167** | |

| **Table 4.7**  **TECHNICAL COMPLIANCE SCORING:** | | | |
| --- | --- | --- | --- |
| **Criterion** | | **Yes =1** | **No = 0** |
| **1. TREATMENT PLANT MEASUREMENTS** | | | |
| **1F: Risk Management Practices** | | | |
| Source to Tap | Is there a risk management system in place? |  | **0** |
|  | Are risk reduction options drawn up and implemented based on the risk assessment? |  | **0** |
|  | Are the risk assessments carried out on a regular basis? |  | **0** |
| Maximum possible score for ***Risk Management Practices*** | | **3** | |
| Total score attained for ***Risk Management Practices*** | | **0** | |
| Weight for ***Risk Management Practices*** | | **0.1** | |
| Total weighted score for ***Risk Management Practices*** | | **0** | |

| **TABLE 4.8**  **TECHNICAL COMPLIANCE SCORING:**  **Total Weighted Scoring for Mutale Water Treatment Plant 60.3 %** | |
| --- | --- |
| **Compliance Criterion** | **Weighted Score** |
| **1. TREATMENT PLANT MEASUREMENTS** | |
| 1A: Design Aspects | **0.094** |
| 1B: Operation Monitoring Practices | **0.092** |
| 1C: Compliance (Final Water Quality) Monitoring Practices | **0.15** |
| 1D: Plant Monitoring Practices | **0.1** |
| 1E: Maintenance Practices | **0.167** |
| 1F: Risk Management Practices | **0** |
| TOTAL WEIGHTED SCORE | **1.0** |

**3.5 Technical compliance rating of small water treatment plants**

Based on the Technical Compliance Scoring system used above and the final total weighted score calculated for the specific water treatment plant, the plant can then be rated according to the following Technical Compliance Rating:

| **TABLE 4.9**  **TECHNICAL COMPLIANCE RATING** | |
| --- | --- |
| **Total Weighted Score** | **Rating Description** |
| **0 – 50** | Class 3 Compliance: Total non-compliance; serious and immediate intervention required (TAC) |
| **50 – 90** | Class 2 Compliance: Serious challenges requiring attention and improvement |
| **90 – 100** | Class 1 Compliance: Acceptable compliance |

The following chapter focuses on the corrective actions for non-technical compliance that can be recorded in a drinking water supply.

1. **august 2009**
2. **TSHEDZA PACKAGE PLANT**

| **Table 5.1**  **TECHNICAL COMPLIANCE SCORING** | |
| --- | --- |
| **Criterion** | **Weight** |
| **1. TREATMENT PLANT MEASUREMENTS** | |
| 1A: Design Aspects | **0.1** |
| 1B: Operation Monitoring Practices | **0.2** |
| 1C: Compliance (Final Water Quality) Monitoring Practices | **0.3** |
| 1D: Plant Monitoring Practices | **0.1** |
| 1E: Maintenance Practices | **0.2** |
| 1F: Risk Management Practices | **0.1** |
| **TOTAL** | **1.0** |

| **Table 5.2**  **TECHNICAL COMPLIANCE SCORING:** | | | |
| --- | --- | --- | --- |
| **Criterion** | | **Yes =1** | **No = 0** |
| **1. TREATMENT PLANT MEASUREMENTS** | | | |
| **1A: Design Aspects** | | | |
| Rapid Mixing | Is the mixing intensity sufficient? | 1 |  |
|  | Is the mixing time sufficient? | 1 |  |
|  | Are chemicals dosed at the correct points? | 1 |  |
| Flocculation | Is flocculation intensity at correct levels (not too high; not too low)? |  | N/A |
|  | Is flocculation time sufficient (at least 15 minutes)? |  | N/A |
|  | Can the flocculation channels or tanks be cleaned easily? |  | N/A |
| Sedimentation | Is floc break-up prevented in the inlet to the sedimentation tank? |  | N/A |
|  | Is the upflow velocity in the sedimentation tank sufficiently low? |  | N/A |
|  | Is the weir overflow rate sufficiently low? |  | N/A |
|  | Can the sedimentation tank(s) be desludged easily and effectively? |  | N/A |
| Filtration | Is filtration rate sufficiently low to ensure efficient filtration? | 1 |  |
|  | Is the filtration depth and filter media suitable for the type of raw water? | 1 |  |
|  | Is the underdrain system acceptable to ensure sustainable filtration? | 1 |  |
|  | Is the backwashing system suitable to ensure effective cleaning of sand filters? | 1 |  |
| Disinfection | Can chlorine (or other disinfectant) be dosed at sufficient levels for the whole range of inflow rates and chlorine demands of the raw water (i.e. are dosing pumps sized correctly)? | 1 |  |
|  | Are monitoring systems in place to ensure timeous changing of chlorine cylinders or to replenish disinfectant make-up solutions? | 1 |  |
| Dosing systems | Have dosage systems (coagulation; pH adjustment; oxidation; disinfection; stabilisation) been installed to ensure easy maintenance and trouble-free operation? | 1 |  |
| Maximum possible score for ***Design Aspects*** | | **17** | |
| Total score attained for ***Design Aspects*** | | **10** | |
| Weight for ***Design Aspects*** | | **0.1** | |
| Total weighted score for ***Design Aspects*** | | **0.095** | |

| **Table 5.3**  **TECHNICAL COMPLIANCE SCORING:** | | | |
| --- | --- | --- | --- |
| **Criterion** | | **Yes =1** | **No = 0** |
| **1. TREATMENT PLANT MEASUREMENTS** | | | |
| **1B: Operation Monitoring Practices** | | | |
| Coagulation and Flocculation | Do the process controllers know how much they are dosing (even qualitatively)? | **1** |  |
|  | Can the process controllers adjust the dosage rates and monitor how much is dosed? | **1** |  |
|  | Do they have a programme for monitoring floc formation? |  | N/A |
| Sedimentation | Is the floc blanket observed and dislodging practices based thereon? |  | N/A |
|  | Is the overflow weir kept clean? |  | N/A |
|  | Is the flow of more than one sedimentation tank distributed evenly? |  | N/A |
| Filtration | Is backwashing done properly (at the right time and according to the correct procedure)? | **1** |  |
|  | Is the quality of the filtrate monitored on a regular basis? |  | N/A |
|  | Is excessive head loss development or turbidity breakthrough monitored? |  | N/A |
| Disinfection | Is the chlorine dosed according to previously determined chlorine demand and/or by maintaining acceptable chlorine residual in the final water? |  | **0** |
|  | Is the chlorine residual measured correctly and at the suggested frequency? |  | **0** |
| Stabilisation | Is the stability of the final water determined? |  | **0** |
|  | Is the process controller familiar with the reason for stabilisation and how it can be affected and controlled? | **1** |  |
| **Maximum possible score for *Operation Monitoring Practices*** | | **13** | |
| **Total score attained for *Operation Monitoring Practices*** | | **4** | |
| **Weight for *Operation Monitoring Practices*** | | **0.2** | |
| **Total weighted score for *Operation Monitoring Practices*** | | **0.062** | |

| **Table 5.4**  **TECHNICAL COMPLIANCE SCORING:** | | | |
| --- | --- | --- | --- |
| **Criterion** | | **Yes =1** | **No = 0** |
| **1. TREATMENT PLANT MEASUREMENTS** | | | |
| **1C: Compliance Monitoring Practices** | | | |
| Final water quality | Is the free chlorine residual sufficient? | **1** |  |
|  | Is the turbidity less than 1.0 NTU? | **1** |  |
|  | Is the pH between 6.0 and 9.0? | **1** |  |
|  | Is the colour less than 10 mg/L as Pt? |  | **N/A** |
|  | Is the final water free of any coliform indicator organisms? | **1** |  |
|  | Is the electrical conductivity less than 70 mS/m? |  | **N/A** |
|  | Is the iron level less than 0.2 mg/L as Fe? |  | **N/A** |
|  | Is the aluminium level less than 0.2 mg/L as Al? |  | **N/A** |
| Maximum possible score for ***Compliance Monitoring Practices*** | | **8** | |
| Total score attained for ***Compliance Monitoring Practices*** | | **4** | |
| Weight for ***Compliance Monitoring Practices*** | | **0.3** | |
| Total weighted score for ***Compliance Monitoring Practices*** | | **0.15** | |

| **Table 5.5**  **TECHNICAL COMPLIANCE SCORING:** | | | |
| --- | --- | --- | --- |
| **Criterion** | | **Yes =1** | **No = 0** |
| **1. TREATMENT PLANT MEASUREMENTS** | | | |
| **1D: Plant Monitoring Practices** | | | |
| Plant Infrastructure and Equipment | Are all the pumps in working order? | **1** |  |
|  | Can all the valves open and close properly? | **1** |  |
|  | Are all leaks repaired? | **1** |  |
|  | Are all blockages in pipes cleared? | **1** |  |
|  | Are all unit treatment processes and equipment accessible? | **1** |  |
| Maximum possible score for ***Plant Monitoring Practices*** | | **5** | |
| Total score attained for ***Plant Monitoring Practices*** | | **5** | |
| Weight for ***Plant Monitoring Practices*** | | **0.1** | |
| Total weighted score for ***Plant Monitoring Practices*** | | **0.1** | |

| **Table 5.6**  **TECHNICAL COMPLIANCE SCORING:** | | | |
| --- | --- | --- | --- |
| **Criterion** | | **Yes =1** | **No = 0** |
| **1. TREATMENT PLANT MEASUREMENTS** | | | |
| 1E: Maintenance Practices | | | |
| Overall Treatment Plant | Is back-up service available? | **1** |  |
|  | Is there opportunity for training of the plant personnel in using the sophisticated equipment? | **1** |  |
|  | Are spare-parts readily available? | **1** |  |
|  | Can spare parts be replaced quickly? | **1** |  |
|  | Are spare parts affordable by the municipality? | **1** |  |
|  | Is back-up service readily available? | **1** |  |
|  | Can service providers be on site at short notice? | **1** |  |
|  | Is this service reliable? | **1** |  |
|  | Can the community do certain parts of the maintenance themselves? |  | **N/A** |
|  | Are there adequate communication facilities between the plant and management, authorities, service providers, suppliers and consultants? | **1** |  |
|  | Can this be readily improved? | **1** |  |
|  | Can the plant personnel or persons in the community perform their own maintenance (or parts thereof)? |  | **N/A** |
|  | Can they be trained and are such training facilities available? | **1** |  |
|  | Is there good access to the plant by motor vehicles and delivery trucks? | **1** |  |
|  | Can the existing access routes be improved with existing funds? | **1** |  |
|  | Are funds available? | **1** |  |
|  | Is there possibility of alternative sources of funding for maintenance? | **1** |  |
| Maximum possible score for ***Maintenance Practices*** | | **18** | |
| Total score attained for ***Maintenance Practices*** | | **15** | |
| Weight for ***Maintenance Practices*** | | **0.2** | |
| Total weighted score for ***Maintenance Practices*** | | **0.167** | |

| **Table 5.7**  **TECHNICAL COMPLIANCE SCORING:** | | | |
| --- | --- | --- | --- |
| **Criterion** | | **Yes =1** | **No = 0** |
| **1. TREATMENT PLANT MEASUREMENTS** | | | |
| **1F: Risk Management Practices** | | | |
| Source to Tap | Is there a risk management system in place? |  | **0** |
|  | Are risk reduction options drawn up and implemented based on the risk assessment? |  | **0** |
|  | Are the risk assessments carried out on a regular basis? |  | **0** |
| Maximum possible score for ***Risk Management Practices*** | | **3** | |
| Total score attained for ***Risk Management Practices*** | | **0** | |
| Weight for ***Risk Management Practices*** | | **0.1** | |
| Total weighted score for ***Risk Management Practices*** | | **0.0** | |

| **TABLE 5.8**  **TECHNICAL COMPLIANCE SCORING:**  **Total Weighted Scoring for Tshedza Package plant 53.8 %** | |
| --- | --- |
| **Compliance Criterion** | **Weighted Score** |
| **1. TREATMENT PLANT MEASUREMENTS** | |
| 1A: Design Aspects | **0.059** |
| 1B: Operation Monitoring Practices | **0.062** |
| 1C: Compliance (Final Water Quality) Monitoring Practices | **0.15** |
| 1D: Plant Monitoring Practices | **0.1** |
| 1E: Maintenance Practices | **0.017** |
| 1F: Risk Management Practices | **0.0** |
| TOTAL WEIGHTED SCORE | **1.0** |

**3.5 Technical compliance rating of small water treatment plants**

Based on the Technical Compliance Scoring system used above and the final total weighted score calculated for the specific water treatment plant, the plant can then be rated according to the following Technical Compliance Rating:

| **TABLE 5.9**  **TECHNICAL COMPLIANCE RATING** | |
| --- | --- |
| **Total Weighted Score** | **Rating Description** |
| **0 – 50** | Class 3 Compliance: Total non-compliance; serious and immediate intervention required (TAC) |
| **50 – 90** | Class 2 Compliance: Serious challenges requiring attention and improvement |
| **90 – 100** | Class 1 Compliance: Acceptable compliance |

The following chapter focuses on the corrective actions for non-technical compliance that can be recorded in a drinking water supply.

1. **August 2009**
2. **DZINGAHE PACKAGE PLANT-**

| **Table 6.1**  **TECHNICAL COMPLIANCE SCORING** | |
| --- | --- |
| **Criterion** | **Weight** |
| **1. TREATMENT PLANT MEASUREMENTS** | |
| 1A: Design Aspects | **0.1** |
| 1B: Operation Monitoring Practices | **0.2** |
| 1C: Compliance (Final Water Quality) Monitoring Practices | **0.3** |
| 1D: Plant Monitoring Practices | **0.1** |
| 1E: Maintenance Practices | **0.2** |
| 1F: Risk Management Practices | **0.1** |
| **TOTAL** | **1.0** |

| **Table 6.2**  **TECHNICAL COMPLIANCE SCORING:** | | | |
| --- | --- | --- | --- |
| **Criterion** | | **Yes =1** | **No = 0** |
| **1. TREATMENT PLANT MEASUREMENTS** | | | |
| **1A: Design Aspects** | | | |
| Rapid Mixing | Is the mixing intensity sufficient? | 1 |  |
|  | Is the mixing time sufficient? | 1 |  |
|  | Are chemicals dosed at the correct points? | 1 |  |
| Flocculation | Is flocculation intensity at correct levels (not too high; not too low)? |  | N/A |
|  | Is flocculation time sufficient (at least 15 minutes)? |  | N/A |
|  | Can the flocculation channels or tanks be cleaned easily? |  | N/A |
| Sedimentation | Is floc break-up prevented in the inlet to the sedimentation tank? |  | N/A |
|  | Is the upflow velocity in the sedimentation tank sufficiently low? |  | N/A |
|  | Is the weir overflow rate sufficiently low? |  | N/A |
|  | Can the sedimentation tank(s) be desludged easily and effectively? |  | N/A |
| Filtration | Is filtration rate sufficiently low to ensure efficient filtration? | 1 |  |
|  | Is the filtration depth and filter media suitable for the type of raw water? | 1 |  |
|  | Is the underdrain system acceptable to ensure sustainable filtration? | 1 |  |
|  | Is the backwashing system suitable to ensure effective cleaning of sand filters? | 1 |  |
| Disinfection | Can chlorine (or other disinfectant) be dosed at sufficient levels for the whole range of inflow rates and chlorine demands of the raw water (i.e. are dosing pumps sized correctly)? | 1 |  |
|  | Are monitoring systems in place to ensure timeous changing of chlorine cylinders or to replenish disinfectant make-up solutions? | 1 |  |
| Dosing systems | Have dosage systems (coagulation; pH adjustment; oxidation; disinfection; stabilisation) been installed to ensure easy maintenance and trouble-free operation? | 1 |  |
| Maximum possible score for ***Design Aspects*** | | **17** | |
| Total score attained for ***Design Aspects*** | | **9** | |
| Weight for ***Design Aspects*** | | **0.1** | |
| Total weighted score for ***Design Aspects*** | | **0.053** | |

| **Table 6.3**  **TECHNICAL COMPLIANCE SCORING:** | | | |
| --- | --- | --- | --- |
| **Criterion** | | **Yes =1** | **No = 0** |
| **1. TREATMENT PLANT MEASUREMENTS** | | | |
| **1B: Operation Monitoring Practices** | | | |
| Coagulation and Flocculation | Do the process controllers know how much they are dosing (even qualitatively)? | **1** |  |
|  | Can the process controllers adjust the dosage rates and monitor how much is dosed? | **1** |  |
|  | Do they have a programme for monitoring floc formation? |  | N/A |
| Sedimentation | Is the floc blanket observed and dislodging practices based thereon? |  | N/A |
|  | Is the overflow weir kept clean? |  | N/A |
|  | Is the flow of more than one sedimentation tank distributed evenly? |  | N/A |
| Filtration | Is backwashing done properly (at the right time and according to the correct procedure)? | **1** |  |
|  | Is the quality of the filtrate monitored on a regular basis? |  | N/A |
|  | Is excessive head loss development or turbidity breakthrough monitored? |  | N/A |
| Disinfection | Is the chlorine dosed according to previously determined chlorine demand and/or by maintaining acceptable chlorine residual in the final water? |  | **0** |
|  | Is the chlorine residual measured correctly and at the suggested frequency? |  | **0** |
| Stabilisation | Is the stability of the final water determined? |  | **0** |
|  | Is the process controller familiar with the reason for stabilisation and how it can be affected and controlled? |  | **0** |
| **Maximum possible score for *Operation Monitoring Practices*** | | **13** | |
| **Total score attained for *Operation Monitoring Practices*** | | **3** | |
| **Weight for *Operation Monitoring Practices*** | | **0.2** | |
| **Total weighted score for *Operation Monitoring Practices*** | | **0.046** | |

| **Table 6.4**  **TECHNICAL COMPLIANCE SCORING:** | | | |
| --- | --- | --- | --- |
| **Criterion** | | **Yes =1** | **No = 0** |
| **1. TREATMENT PLANT MEASUREMENTS** | | | |
| **1C: Compliance Monitoring Practices** | | | |
| Final water quality | Is the free chlorine residual sufficient? |  | **0** |
|  | Is the turbidity less than 1.0 NTU? |  | **0** |
|  | Is the pH between 6.0 and 9.0? | **1** |  |
|  | Is the colour less than 10 mg/L as Pt? |  | **N/A** |
|  | Is the final water free of any coliform indicator organisms? | **1** |  |
|  | Is the electrical conductivity less than 70 mS/m? |  | **N/A** |
|  | Is the iron level less than 0.2 mg/L as Fe? |  | **N/A** |
|  | Is the aluminium level less than 0.2 mg/L as Al? |  | **N/A** |
| Maximum possible score for ***Compliance Monitoring Practices*** | | **8** | |
| Total score attained for ***Compliance Monitoring Practices*** | | **2** | |
| Weight for ***Compliance Monitoring Practices*** | | **0.3** | |
| Total weighted score for ***Compliance Monitoring Practices*** | | **0.075** | |

| **Table 6.5**  **TECHNICAL COMPLIANCE SCORING:** | | | |
| --- | --- | --- | --- |
| **Criterion** | | **Yes =1** | **No = 0** |
| **1. TREATMENT PLANT MEASUREMENTS** | | | |
| **1D: Plant Monitoring Practices** | | | |
| Plant Infrastructure and Equipment | Are all the pumps in working order? | **1** |  |
|  | Can all the valves open and close properly? | **1** |  |
|  | Are all leaks repaired? | **1** |  |
|  | Are all blockages in pipes cleared? | **1** |  |
|  | Are all unit treatment processes and equipment accessible? | **1** |  |
| Maximum possible score for ***Plant Monitoring Practices*** | | **5** | |
| Total score attained for ***Plant Monitoring Practices*** | | **5** | |
| Weight for ***Plant Monitoring Practices*** | | **0.1** | |
| Total weighted score for ***Plant Monitoring Practices*** | | **0.1** | |

| **Table 6.6**  **TECHNICAL COMPLIANCE SCORING:** | | | |
| --- | --- | --- | --- |
| **Criterion** | | **Yes =1** | **No = 0** |
| **1. TREATMENT PLANT MEASUREMENTS** | | | |
| 1E: Maintenance Practices | | | |
| Overall Treatment Plant | Is back-up service available? | **1** |  |
|  | Is there opportunity for training of the plant personnel in using the sophisticated equipment? | **1** |  |
|  | Are spare-parts readily available? | **1** |  |
|  | Can spare parts be replaced quickly? | **1** |  |
|  | Are spare parts affordable by the municipality? | **1** |  |
|  | Is back-up service readily available? | **1** |  |
|  | Can service providers be on site at short notice? | **1** |  |
|  | Is this service reliable? | **1** |  |
|  | Can the community do certain parts of the maintenance themselves? |  | **N/A** |
|  | Are there adequate communication facilities between the plant and management, authorities, service providers, suppliers and consultants? | **1** |  |
|  | Can this be readily improved? | **1** |  |
|  | Can the plant personnel or persons in the community perform their own maintenance (or parts thereof)? |  | **N/A** |
|  | Can they be trained and are such training facilities available? | **1** |  |
|  | Is there good access to the plant by motor vehicles and delivery trucks? | **1** |  |
|  | Can the existing access routes be improved with existing funds? | **1** |  |
|  | Are funds available? | **1** |  |
|  | Is there possibility of alternative sources of funding for maintenance? | **1** |  |
| Maximum possible score for ***Maintenance Practices*** | | **18** | |
| Total score attained for ***Maintenance Practices*** | | **15** | |
| Weight for ***Maintenance Practices*** | | **0.2** | |
| Total weighted score for ***Maintenance Practices*** | | **0.167** | |

| **Table 6.7**  **TECHNICAL COMPLIANCE SCORING:** | | | |
| --- | --- | --- | --- |
| **Criterion** | | **Yes =1** | **No = 0** |
| **1. TREATMENT PLANT MEASUREMENTS** | | | |
| **1F: Risk Management Practices** | | | |
| Source to Tap | Is there a risk management system in place? |  | **0** |
|  | Are risk reduction options drawn up and implemented based on the risk assessment? |  | **0** |
|  | Are the risk assessments carried out on a regular basis? |  | **0** |
| Maximum possible score for ***Risk Management Practices*** | | **3** | |
| Total score attained for ***Risk Management Practices*** | | **0** | |
| Weight for ***Risk Management Practices*** | | **0.1** | |
| Total weighted score for ***Risk Management Practices*** | | **0** | |

| **TABLE 6.8**  **TECHNICAL COMPLIANCE SCORING:**  **Total Weighted Scoring for Dzingahe Package plant 44.1 %** | |
| --- | --- |
| **Compliance Criterion** | **Weighted Score** |
| **1. TREATMENT PLANT MEASUREMENTS** | |
| 1A: Design Aspects | **0.053** |
| 1B: Operation Monitoring Practices | **0.046** |
| 1C: Compliance (Final Water Quality) Monitoring Practices | **0.075** |
| 1D: Plant Monitoring Practices | **0.1** |
| 1E: Maintenance Practices | **0.167** |
| 1F: Risk Management Practices | **0.0** |
| TOTAL WEIGHTED SCORE | **1.0** |

**3.5 Technical compliance rating of small water treatment plants**

Based on the Technical Compliance Scoring system used above and the final total weighted score calculated for the specific water treatment plant, the plant can then be rated according to the following Technical Compliance Rating:

| **TABLE 6.9**  **TECHNICAL COMPLIANCE RATING** | |
| --- | --- |
| **Total Weighted Score** | **Rating Description** |
| **0 – 50** | Class 3 Compliance: Total non-compliance; serious and immediate intervention required (TAC) |
| **50 – 90** | Class 2 Compliance: Serious challenges requiring attention and improvement |
| **90 – 100** | Class 1 Compliance: Acceptable compliance |

The following chapter focuses on the corrective actions for non-technical compliance that can be recorded in a drinking water supply.

**TECHNICAL COMPLIANCE 2011**

**10 May 2011**

1. **MALAMULELE TREATMENT PLANT**

| **Table 1.1**  **TECHNICAL COMPLIANCE SCORING** | |
| --- | --- |
| **Criterion** | **Weight** |
| **1. TREATMENT PLANT MEASUREMENTS** | |
| 1A: Design Aspects | **0.1** |
| 1B: Operation Monitoring Practices | **0.2** |
| 1C: Compliance (Final Water Quality) Monitoring Practices | **0.3** |
| 1D: Plant Monitoring Practices | **0.1** |
| 1E: Maintenance Practices | **0.2** |
| 1F: Risk Management Practices | **0.1** |
| **TOTAL** | **1.0** |

| **Table 1.2**  **TECHNICAL COMPLIANCE SCORING:** | | | |
| --- | --- | --- | --- |
| **Criterion** | | **Yes =1** | **No = 0** |
| **1. TREATMENT PLANT MEASUREMENTS** | | | |
| **1A: Design Aspects** | | | |
| Rapid Mixing | Is the mixing intensity sufficient? | 1 |  |
|  | Is the mixing time sufficient? | 1 |  |
|  | Are chemicals dosed at the correct points? | 1 |  |
| Flocculation | Is flocculation intensity at correct levels (not too high; not too low)? | 1 |  |
|  | Is flocculation time sufficient (at least 15 minutes)? | 1 |  |
|  | Can the flocculation channels or tanks be cleaned easily? | 1 |  |
| Sedimentation | Is floc break-up prevented in the inlet to the sedimentation tank? | 1 |  |
|  | Is the upflow velocity in the sedimentation tank sufficiently low? | 1 |  |
|  | Is the weir overflow rate sufficiently low? | 1 |  |
|  | Can the sedimentation tank(s) be desludged easily and effectively? | 1 |  |
| Filtration | Is filtration rate sufficiently low to ensure efficient filtration? | 1 |  |
|  | Is the filtration depth and filter media suitable for the type of raw water? | 1 |  |
|  | Is the underdrain system acceptable to ensure sustainable filtration? | 1 |  |
|  | Is the backwashing system suitable to ensure effective cleaning of sand filters? | 1 |  |
| Disinfection | Can chlorine (or other disinfectant) be dosed at sufficient levels for the whole range of inflow rates and chlorine demands of the raw water (i.e. are dosing pumps sized correctly)? | 1 |  |
|  | Are monitoring systems in place to ensure timeous changing of chlorine cylinders or to replenish disinfectant make-up solutions? | 1 |  |
| Dosing systems | Have dosage systems (coagulation; pH adjustment; oxidation; disinfection; stabilisation) been installed to ensure easy maintenance and trouble-free operation? | 1 |  |
| Maximum possible score for ***Design Aspects*** | | **17** | |
| Total score attained for ***Design Aspects*** | | **17** | |
| Weight for ***Design Aspects*** | | **0.1** | |
| Total weighted score for ***Design Aspects*** | | **0.1** | |

| **Table 1.3**  **TECHNICAL COMPLIANCE SCORING:** | | | |
| --- | --- | --- | --- |
| **Criterion** | | **Yes =1** | **No = 0** |
| **1. TREATMENT PLANT MEASUREMENTS** | | | |
| **1B: Operation Monitoring Practices** | | | |
| Coagulation and Flocculation | Do the process controllers know how much they are dosing (even qualitatively)? | **1** |  |
|  | Can the process controllers adjust the dosage rates and monitor how much is dosed? | **1** |  |
|  | Do they have a programme for monitoring floc formation? |  | **0** |
| Sedimentation | Is the floc blanket observed and dislodging practices based thereon? | **1** |  |
|  | Is the overflow weir kept clean? | **1** |  |
|  | Is the flow of more than one sedimentation tank distributed evenly? | **1** |  |
| Filtration | Is backwashing done properly (at the right time and according to the correct procedure)? | **1** |  |
|  | Is the quality of the filtrate monitored on a regular basis? |  | **0** |
|  | Is excessive head loss development or turbidity breakthrough monitored? |  | **0** |
| Disinfection | Is the chlorine dosed according to previously determined chlorine demand and/or by maintaining acceptable chlorine residual in the final water? |  | **0** |
|  | Is the chlorine residual measured correctly and at the suggested frequency? |  | **0** |
| Stabilisation | Is the stability of the final water determined? | **1** |  |
|  | Is the process controller familiar with the reason for stabilisation and how it can be affected and controlled? | **1** |  |
| **Maximum possible score for *Operation Monitoring Practices*** | | **13** | |
| **Total score attained for *Operation Monitoring Practices*** | | **8** | |
| **Weight for *Operation Monitoring Practices*** | | **0.2** | |
| **Total weighted score for *Operation Monitoring Practices*** | | **0.123** | |

| **Table 1.4**  **TECHNICAL COMPLIANCE SCORING:** | | | |
| --- | --- | --- | --- |
| **Criterion** | | **Yes =1** | **No = 0** |
| **1. TREATMENT PLANT MEASUREMENTS** | | | |
| **1C: Compliance Monitoring Practices** | | | |
| Final water quality | Is the free chlorine residual sufficient? | **1** |  |
|  | Is the turbidity less than 1.0 NTU? |  | **0** |
|  | Is the pH between 6.0 and 9.0? | **1** |  |
|  | Is the colour less than 10 mg/L as Pt? |  | **N/A** |
|  | Is the final water free of any coliform indicator organisms? | **1** |  |
|  | Is the electrical conductivity less than 70 mS/m? |  | **N/A** |
|  | Is the iron level less than 0.2 mg/L as Fe? |  | **N/A** |
|  | Is the aluminium level less than 0.2 mg/L as Al? |  | **N/A** |
| Maximum possible score for ***Compliance Monitoring Practices*** | | **4** | |
| Total score attained for ***Compliance Monitoring Practices*** | | **3** | |
| Weight for ***Compliance Monitoring Practices*** | | **0.3** | |
| Total weighted score for ***Compliance Monitoring Practices*** | | **0.225** | |

| **Table 1.5**  **TECHNICAL COMPLIANCE SCORING:** | | | |
| --- | --- | --- | --- |
| **Criterion** | | **Yes =1** | **No = 0** |
| **1. TREATMENT PLANT MEASUREMENTS** | | | |
| **1D: Plant Monitoring Practices** | | | |
| Plant Infrastructure and Equipment | Are all the pumps in working order? | **1** |  |
|  | Can all the valves open and close properly? | **1** |  |
|  | Are all leaks repaired? | **1** |  |
|  | Are all blockages in pipes cleared? | **1** |  |
|  | Are all unit treatment processes and equipment accessible? | **1** |  |
| Maximum possible score for ***Plant Monitoring Practices*** | | **5** | |
| Total score attained for ***Plant Monitoring Practices*** | | **5** | |
| Weight for ***Plant Monitoring Practices*** | | **0.1** | |
| Total weighted score for ***Plant Monitoring Practices*** | | **0.1** | |

| **Table 1.6**  **TECHNICAL COMPLIANCE SCORING:** | | | |
| --- | --- | --- | --- |
| **Criterion** | | **Yes =1** | **No = 0** |
| **1. TREATMENT PLANT MEASUREMENTS** | | | |
| 1E: Maintenance Practices | | | |
| Overall Treatment Plant | Is back-up service available? | **1** |  |
|  | Is there opportunity for training of the plant personnel in using the sophisticated equipment? | **1** |  |
|  | Are spare-parts readily available? | **1** |  |
|  | Can spare parts be replaced quickly? | **1** |  |
|  | Are spare parts affordable by the municipality? | **1** |  |
|  | Is back-up service readily available? | **1** |  |
|  | Can service providers be on site at short notice? | **1** |  |
|  | Is this service reliable? | **1** |  |
|  | Can the community do certain parts of the maintenance themselves? |  | **N/A** |
|  | Are there adequate communication facilities between the plant and management, authorities, service providers, suppliers and consultants? | **1** |  |
|  | Can this be readily improved? | **1** |  |
|  | Can the plant personnel or persons in the community perform their own maintenance (or parts thereof)? |  | **N/A** |
|  | Can they be trained and are such training facilities available? | **1** |  |
|  | Is there good access to the plant by motor vehicles and delivery trucks? | **1** |  |
|  | Can the existing access routes be improved with existing funds? | **1** |  |
|  | Are funds available? | **1** |  |
|  | Is there possibility of alternative sources of funding for maintenance? | **1** |  |
| Maximum possible score for ***Maintenance Practices*** | | **18** | |
| Total score attained for ***Maintenance Practices*** | | **15** | |
| Weight for ***Maintenance Practices*** | | **0.2** | |
| Total weighted score for ***Maintenance Practices*** | | **0.167** | |

| **Table 1.7**  **TECHNICAL COMPLIANCE SCORING:** | | | |
| --- | --- | --- | --- |
| **Criterion** | | **Yes =1** | **No = 0** |
| **1. TREATMENT PLANT MEASUREMENTS** | | | |
| **1F: Risk Management Practices** | | | |
| Source to Tap | Is there a risk management system in place? | **1** |  |
|  | Are risk reduction options drawn up and implemented based on the risk assessment? | **1** |  |
|  | Are the risk assessments carried out on a regular basis? |  | **0** |
| Maximum possible score for ***Risk Management Practices*** | | **3** | |
| Total score attained for ***Risk Management Practices*** | | **2** | |
| Weight for ***Risk Management Practices*** | | **0.1** | |
| Total weighted score for ***Risk Management Practices*** | | **0.067** | |

| **TABLE 1.8**  **TECHNICAL COMPLIANCE SCORING:**  **Total Weighted Scoring for Malamulele Treatment Plant 78.17 %** | |
| --- | --- |
| **Compliance Criterion** | **Weighted Score** |
| **1. TREATMENT PLANT MEASUREMENTS** | |
| 1A: Design Aspects | **0.1** |
| 1B: Operation Monitoring Practices | **0.123** |
| 1C: Compliance (Final Water Quality) Monitoring Practices | **0.225** |
| 1D: Plant Monitoring Practices | **0.1** |
| 1E: Maintenance Practices | **0.167** |
| 1F: Risk Management Practices | **0.067** |
| TOTAL WEIGHTED SCORE | **1.0** |

**3.5 Technical compliance rating of small water treatment plants**

Based on the Technical Compliance Scoring system used above and the final total weighted score calculated for the specific water treatment plant, the plant can then be rated according to the following Technical Compliance Rating:

| **TABLE 1.9**  **TECHNICAL COMPLIANCE RATING** | |
| --- | --- |
| **Total Weighted Score** | **Rating Description** |
| **0 – 50** | Class 3 Compliance: Total non-compliance; serious and immediate intervention required (TAC) |
| **50 – 90** | Class 2 Compliance: Serious challenges requiring attention and improvement |
| **90 – 100** | Class 1 Compliance: Acceptable compliance |

The following chapter focuses on the corrective actions for non-technical compliance that can be recorded in a drinking water supply.

**13 may 2011**

1. **Vondo Water Scheme**

| **Table 2.1**  **TECHNICAL COMPLIANCE SCORING** | |
| --- | --- |
| **Criterion** | **Weight** |
| **1. TREATMENT PLANT MEASUREMENTS** | |
| 1A: Design Aspects | **0.1** |
| 1B: Operation Monitoring Practices | **0.2** |
| 1C: Compliance (Final Water Quality) Monitoring Practices | **0.3** |
| 1D: Plant Monitoring Practices | **0.1** |
| 1E: Maintenance Practices | **0.2** |
| 1F: Risk Management Practices | **0.1** |
| **TOTAL** | **1.0** |

| **Table 2.2**  **TECHNICAL COMPLIANCE SCORING:** | | | |
| --- | --- | --- | --- |
| **Criterion** | | **Yes =1** | **No = 0** |
| **1. TREATMENT PLANT MEASUREMENTS** | | | |
| **1A: Design Aspects** | | | |
| Rapid Mixing | Is the mixing intensity sufficient? | 1 |  |
|  | Is the mixing time sufficient? | 1 |  |
|  | Are chemicals dosed at the correct points? | 1 |  |
| Flocculation | Is flocculation intensity at correct levels (not too high; not too low)? | 1 |  |
|  | Is flocculation time sufficient (at least 15 minutes)? | 1 |  |
|  | Can the flocculation channels or tanks be cleaned easily? | 1 |  |
| Sedimentation | Is floc break-up prevented in the inlet to the sedimentation tank? |  | N/A |
|  | Is the upflow velocity in the sedimentation tank sufficiently low? |  | N/A |
|  | Is the weir overflow rate sufficiently low? |  | N/A |
|  | Can the sedimentation tank(s) be desludged easily and effectively? |  | N/A |
| Filtration | Is filtration rate sufficiently low to ensure efficient filtration? | 1 |  |
|  | Is the filtration depth and filter media suitable for the type of raw water? | 1 |  |
|  | Is the underdrain system acceptable to ensure sustainable filtration? | 1 |  |
|  | Is the backwashing system suitable to ensure effective cleaning of sand filters? | 1 |  |
| Disinfection | Can chlorine (or other disinfectant) be dosed at sufficient levels for the whole range of inflow rates and chlorine demands of the raw water (i.e. are dosing pumps sized correctly)? | 1 |  |
|  | Are monitoring systems in place to ensure timeous changing of chlorine cylinders or to replenish disinfectant make-up solutions? | 1 |  |
| Dosing systems | Have dosage systems (coagulation; pH adjustment; oxidation; disinfection; stabilisation) been installed to ensure easy maintenance and trouble-free operation? | 1 |  |
| Maximum possible score for ***Design Aspects*** | | **17** | |
| Total score attained for ***Design Aspects*** | | **13** | |
| Weight for ***Design Aspects*** | | **0.1** | |
| Total weighted score for ***Design Aspects*** | | **0.076** | |

| **Table 2.3**  **TECHNICAL COMPLIANCE SCORING:** | | | |
| --- | --- | --- | --- |
| **Criterion** | | **Yes =1** | **No = 0** |
| **1. TREATMENT PLANT MEASUREMENTS** | | | |
| **1B: Operation Monitoring Practices** | | | |
| Coagulation and Flocculation | Do the process controllers know how much they are dosing (even qualitatively)? | **1** |  |
|  | Can the process controllers adjust the dosage rates and monitor how much is dosed? | **1** |  |
|  | Do they have a programme for monitoring floc formation? |  | **N/A** |
| Sedimentation | Is the floc blanket observed and dislodging practices based thereon? |  | **N/A** |
|  | Is the overflow weir kept clean? |  | **N/A** |
|  | Is the flow of more than one sedimentation tank distributed evenly? |  | **N/A** |
| Filtration | Is backwashing done properly (at the right time and according to the correct procedure)? | **1** |  |
|  | Is the quality of the filtrate monitored on a regular basis? |  | **0** |
|  | Is excessive head loss development or turbidity breakthrough monitored? |  | **N/A** |
| Disinfection | Is the chlorine dosed according to previously determined chlorine demand and/or by maintaining acceptable chlorine residual in the final water? | **1** |  |
|  | Is the chlorine residual measured correctly and at the suggested frequency? | **1** |  |
| Stabilisation | Is the stability of the final water determined? | **1** |  |
|  | Is the process controller familiar with the reason for stabilisation and how it can be affected and controlled? | **1** |  |
| **Maximum possible score for *Operation Monitoring Practices*** | | **13** | |
| **Total score attained for *Operation Monitoring Practices*** | | **7** | |
| **Weight for *Operation Monitoring Practices*** | | **0.2** | |
| **Total weighted score for *Operation Monitoring Practices*** | | **0.108** | |

| **Table 2.4**  **TECHNICAL COMPLIANCE SCORING:** | | | |
| --- | --- | --- | --- |
| **Criterion** | | **Yes =1** | **No = 0** |
| **1. TREATMENT PLANT MEASUREMENTS** | | | |
| **1C: Compliance Monitoring Practices** | | | |
| Final water quality | Is the free chlorine residual sufficient? |  | **0** |
|  | Is the turbidity less than 1.0 NTU? |  | **0** |
|  | Is the pH between 6.0 and 9.0? | **1** |  |
|  | Is the colour less than 10 mg/L as Pt? |  | **N/A** |
|  | Is the final water free of any coliform indicator organisms? |  | **0** |
|  | Is the electrical conductivity less than 70 mS/m? |  | **N/A** |
|  | Is the iron level less than 0.2 mg/L as Fe? |  | **N/A** |
|  | Is the aluminium level less than 0.2 mg/L as Al? |  | **N/A** |
| Maximum possible score for ***Compliance Monitoring Practices*** | | **4** | |
| Total score attained for ***Compliance Monitoring Practices*** | | **1** | |
| Weight for ***Compliance Monitoring Practices*** | | **0.3** | |
| Total weighted score for ***Compliance Monitoring Practices*** | | **0.075** | |

| **Table 2.5**  **TECHNICAL COMPLIANCE SCORING:** | | | |
| --- | --- | --- | --- |
| **Criterion** | | **Yes =1** | **No = 0** |
| **1. TREATMENT PLANT MEASUREMENTS** | | | |
| **1D: Plant Monitoring Practices** | | | |
| Plant Infrastructure and Equipment | Are all the pumps in working order? | **1** |  |
|  | Can all the valves open and close properly? | **1** |  |
|  | Are all leaks repaired? | **1** |  |
|  | Are all blockages in pipes cleared? | **1** |  |
|  | Are all unit treatment processes and equipment accessible? | **1** |  |
| Maximum possible score for ***Plant Monitoring Practices*** | | **5** | |
| Total score attained for ***Plant Monitoring Practices*** | | **5** | |
| Weight for ***Plant Monitoring Practices*** | | **0.1** | |
| Total weighted score for ***Plant Monitoring Practices*** | | **0.1** | |

| **Table 2.6**  **TECHNICAL COMPLIANCE SCORING:** | | | |
| --- | --- | --- | --- |
| **Criterion** | | **Yes =1** | **No = 0** |
| **1. TREATMENT PLANT MEASUREMENTS** | | | |
| 1E: Maintenance Practices | | | |
| Overall Treatment Plant | Is back-up service available? | **1** |  |
|  | Is there opportunity for training of the plant personnel in using the sophisticated equipment? | **1** |  |
|  | Are spare-parts readily available? | **1** |  |
|  | Can spare parts be replaced quickly? | **1** |  |
|  | Are spare parts affordable by the municipality? | **1** |  |
|  | Is back-up service readily available? | **1** |  |
|  | Can service providers be on site at short notice? | **1** |  |
|  | Is this service reliable? | **1** |  |
|  | Can the community do certain parts of the maintenance themselves? |  | **N/A** |
|  | Are there adequate communication facilities between the plant and management, authorities, service providers, suppliers and consultants? | **1** |  |
|  | Can this be readily improved? | **1** |  |
|  | Can the plant personnel or persons in the community perform their own maintenance (or parts thereof)? |  | **N/A** |
|  | Can they be trained and are such training facilities available? | **1** |  |
|  | Is there good access to the plant by motor vehicles and delivery trucks? | **1** |  |
|  | Can the existing access routes be improved with existing funds? | **1** |  |
|  | Are funds available? | **1** |  |
|  | Is there possibility of alternative sources of funding for maintenance? | **1** |  |
| Maximum possible score for ***Maintenance Practices*** | | **18** | |
| Total score attained for ***Maintenance Practices*** | | **15** | |
| Weight for ***Maintenance Practices*** | | **0.2** | |
| Total weighted score for ***Maintenance Practices*** | | **0.167** | |

| **Table 2.7**  **TECHNICAL COMPLIANCE SCORING:** | | | |
| --- | --- | --- | --- |
| **Criterion** | | **Yes =1** | **No = 0** |
| **1. TREATMENT PLANT MEASUREMENTS** | | | |
| **1F: Risk Management Practices** | | | |
| Source to Tap | Is there a risk management system in place? | **1** |  |
|  | Are risk reduction options drawn up and implemented based on the risk assessment? | **1** |  |
|  | Are the risk assessments carried out on a regular basis? |  | **0** |
| Maximum possible score for ***Risk Management Practices*** | | **3** | |
| Total score attained for ***Risk Management Practices*** | | **2** | |
| Weight for ***Risk Management Practices*** | | **0.1** | |
| Total weighted score for ***Risk Management Practices*** | | **0.067** | |

| **TABLE 2.8**  **TECHNICAL COMPLIANCE SCORING:**  **Total Weighted Scoring for Vondo Water Scheme 49.4 %** | |
| --- | --- |
| **Compliance Criterion** | **Weighted Score** |
| **1. TREATMENT PLANT MEASUREMENTS** | |
| 1A: Design Aspects | **0.076** |
| 1B: Operation Monitoring Practices | **0.108** |
| 1C: Compliance (Final Water Quality) Monitoring Practices | **0.075** |
| 1D: Plant Monitoring Practices | **0.10** |
| 1E: Maintenance Practices | **0.167** |
| 1F: Risk Management Practices | **0.067** |
| TOTAL WEIGHTED SCORE | **1.0** |

**3.5 Technical compliance rating of small water treatment plants**

Based on the Technical Compliance Scoring system used above and the final total weighted score calculated for the specific water treatment plant, the plant can then be rated according to the following Technical Compliance Rating:

| **TABLE 2.9**  **TECHNICAL COMPLIANCE RATING** | |
| --- | --- |
| **Total Weighted Score** | **Rating Description** |
| **0 – 50** | Class 3 Compliance: Total non-compliance; serious and immediate intervention required (TAC) |
| **50 – 90** | Class 2 Compliance: Serious challenges requiring attention and improvement |
| **90 – 100** | Class 1 Compliance: Acceptable compliance |

The following chapter focuses on the corrective actions for non-technical compliance that can be recorded in a drinking water supply.

1. **May 2011**
2. **MUTSHEDZI TREATMENT NEW PLANT**

| **Table 3.1**  **TECHNICAL COMPLIANCE SCORING** | |
| --- | --- |
| **Criterion** | **Weight** |
| **1. TREATMENT PLANT MEASUREMENTS** | |
| 1A: Design Aspects | **0.1** |
| 1B: Operation Monitoring Practices | **0.2** |
| 1C: Compliance (Final Water Quality) Monitoring Practices | **0.3** |
| 1D: Plant Monitoring Practices | **0.1** |
| 1E: Maintenance Practices | **0.2** |
| 1F: Risk Management Practices | **0.1** |
| **TOTAL** | **1.0** |

| **Table 3.2**  **TECHNICAL COMPLIANCE SCORING:** | | | |
| --- | --- | --- | --- |
| **Criterion** | | **Yes =1** | **No = 0** |
| **1. TREATMENT PLANT MEASUREMENTS** | | | |
| **1A: Design Aspects** | | | |
| Rapid Mixing | Is the mixing intensity sufficient? | 1 |  |
|  | Is the mixing time sufficient? | 1 |  |
|  | Are chemicals dosed at the correct points? | 1 |  |
| Flocculation | Is flocculation intensity at correct levels (not too high; not too low)? | 1 |  |
|  | Is flocculation time sufficient (at least 15 minutes)? | 1 |  |
|  | Can the flocculation channels or tanks be cleaned easily? | 1 |  |
| Sedimentation | Is floc break-up prevented in the inlet to the sedimentation tank? | 1 |  |
|  | Is the upflow velocity in the sedimentation tank sufficiently low? | 1 |  |
|  | Is the weir overflow rate sufficiently low? | 1 |  |
|  | Can the sedimentation tank(s) be desludged easily and effectively? | 1 |  |
| Filtration | Is filtration rate sufficiently low to ensure efficient filtration? | 1 |  |
|  | Is the filtration depth and filter media suitable for the type of raw water? | 1 |  |
|  | Is the underdrain system acceptable to ensure sustainable filtration? | 1 |  |
|  | Is the backwashing system suitable to ensure effective cleaning of sand filters? | 1 |  |
| Disinfection | Can chlorine (or other disinfectant) be dosed at sufficient levels for the whole range of inflow rates and chlorine demands of the raw water (i.e. are dosing pumps sized correctly)? | 1 |  |
|  | Are monitoring systems in place to ensure timeous changing of chlorine cylinders or to replenish disinfectant make-up solutions? | 1 |  |
| Dosing systems | Have dosage systems (coagulation; pH adjustment; oxidation; disinfection; stabilisation) been installed to ensure easy maintenance and trouble-free operation? | 1 |  |
| Maximum possible score for ***Design Aspects*** | | **17** | |
| Total score attained for ***Design Aspects*** | | **17** | |
| Weight for ***Design Aspects*** | | **0.1** | |
| Total weighted score for ***Design Aspects*** | | **0.1** | |

| **Table 3.3**  **TECHNICAL COMPLIANCE SCORING:** | | | |
| --- | --- | --- | --- |
| **Criterion** | | **Yes =1** | **No = 0** |
| **1. TREATMENT PLANT MEASUREMENTS** | | | |
| **1B: Operation Monitoring Practices** | | | |
| Coagulation and Flocculation | Do the process controllers know how much they are dosing (even qualitatively)? | **1** |  |
|  | Can the process controllers adjust the dosage rates and monitor how much is dosed? | **1** |  |
|  | Do they have a programme for monitoring floc formation? |  | **0** |
| Sedimentation | Is the floc blanket observed and dislodging practices based thereon? | **1** |  |
|  | Is the overflow weir kept clean? | **1** |  |
|  | Is the flow of more than one sedimentation tank distributed evenly? | **1** |  |
| Filtration | Is backwashing done properly (at the right time and according to the correct procedure)? | **1** |  |
|  | Is the quality of the filtrate monitored on a regular basis? |  | **0** |
|  | Is excessive head loss development or turbidity breakthrough monitored? |  | **0** |
| Disinfection | Is the chlorine dosed according to previously determined chlorine demand and/or by maintaining acceptable chlorine residual in the final water? |  | **0** |
|  | Is the chlorine residual measured correctly and at the suggested frequency? |  | **0** |
| Stabilisation | Is the stability of the final water determined? | **1** |  |
|  | Is the process controller familiar with the reason for stabilisation and how it can be affected and controlled? | **1** |  |
| **Maximum possible score for *Operation Monitoring Practices*** | | **13** | |
| **Total score attained for *Operation Monitoring Practices*** | | **9** | |
| **Weight for *Operation Monitoring Practices*** | | **0.2** | |
| **Total weighted score for *Operation Monitoring Practices*** | | **0.138** | |

| **Table 3.4**  **TECHNICAL COMPLIANCE SCORING:** | | | |
| --- | --- | --- | --- |
| **Criterion** | | **Yes =1** | **No = 0** |
| **1. TREATMENT PLANT MEASUREMENTS** | | | |
| **1C: Compliance Monitoring Practices** | | | |
| Final water quality | Is the free chlorine residual sufficient? | **1** |  |
|  | Is the turbidity less than 1.0 NTU? |  | **0** |
|  | Is the pH between 6.0 and 9.0? | **1** |  |
|  | Is the colour less than 10 mg/L as Pt? |  | **N/A** |
|  | Is the final water free of any coliform indicator organisms? | **1** |  |
|  | Is the electrical conductivity less than 70 mS/m? |  | **N/A** |
|  | Is the iron level less than 0.2 mg/L as Fe? |  | **N/A** |
|  | Is the aluminium level less than 0.2 mg/L as Al? |  | **N/A** |
| Maximum possible score for ***Compliance Monitoring Practices*** | | **4** | |
| Total score attained for ***Compliance Monitoring Practices*** | | **3** | |
| Weight for ***Compliance Monitoring Practices*** | | **0.3** | |
| Total weighted score for ***Compliance Monitoring Practices*** | | **0.225** | |

| **Table 3.5**  **TECHNICAL COMPLIANCE SCORING:** | | | |
| --- | --- | --- | --- |
| **Criterion** | | **Yes =1** | **No = 0** |
| **1. TREATMENT PLANT MEASUREMENTS** | | | |
| **1D: Plant Monitoring Practices** | | | |
| Plant Infrastructure and Equipment | Are all the pumps in working order? | **1** |  |
|  | Can all the valves open and close properly? | **1** |  |
|  | Are all leaks repaired? | **1** |  |
|  | Are all blockages in pipes cleared? | **1** |  |
|  | Are all unit treatment processes and equipment accessible? | **1** |  |
| Maximum possible score for ***Plant Monitoring Practices*** | | **5** | |
| Total score attained for ***Plant Monitoring Practices*** | | **5** | |
| Weight for ***Plant Monitoring Practices*** | | **0.1** | |
| Total weighted score for ***Plant Monitoring Practices*** | | **0.1** | |

| **Table 3.6**  **TECHNICAL COMPLIANCE SCORING:** | | | |
| --- | --- | --- | --- |
| **Criterion** | | **Yes =1** | **No = 0** |
| **1. TREATMENT PLANT MEASUREMENTS** | | | |
| 1E: Maintenance Practices | | | |
| Overall Treatment Plant | Is back-up service available? | **1** |  |
|  | Is there opportunity for training of the plant personnel in using the sophisticated equipment? | **1** |  |
|  | Are spare-parts readily available? | **1** |  |
|  | Can spare parts be replaced quickly? | **1** |  |
|  | Are spare parts affordable by the municipality? | **1** |  |
|  | Is back-up service readily available? | **1** |  |
|  | Can service providers be on site at short notice? | **1** |  |
|  | Is this service reliable? | **1** |  |
|  | Can the community do certain parts of the maintenance themselves? |  | **N/A** |
|  | Are there adequate communication facilities between the plant and management, authorities, service providers, suppliers and consultants? | **1** |  |
|  | Can this be readily improved? | **1** |  |
|  | Can the plant personnel or persons in the community perform their own maintenance (or parts thereof)? |  | **N/A** |
|  | Can they be trained and are such training facilities available? | **1** |  |
|  | Is there good access to the plant by motor vehicles and delivery trucks? | **1** |  |
|  | Can the existing access routes be improved with existing funds? | **1** |  |
|  | Are funds available? | **1** |  |
|  | Is there possibility of alternative sources of funding for maintenance? | **1** |  |
| Maximum possible score for ***Maintenance Practices*** | | **18** | |
| Total score attained for ***Maintenance Practices*** | | **15** | |
| Weight for ***Maintenance Practices*** | | **0.2** | |
| Total weighted score for ***Maintenance Practices*** | | **0.167** | |

| **Table 3.7**  **TECHNICAL COMPLIANCE SCORING:** | | | |
| --- | --- | --- | --- |
| **Criterion** | | **Yes =1** | **No = 0** |
| **1. TREATMENT PLANT MEASUREMENTS** | | | |
| **1F: Risk Management Practices** | | | |
| Source to Tap | Is there a risk management system in place? | **1** |  |
|  | Are risk reduction options drawn up and implemented based on the risk assessment? | **1** |  |
|  | Are the risk assessments carried out on a regular basis? |  | **0** |
| Maximum possible score for ***Risk Management Practices*** | | **3** | |
| Total score attained for ***Risk Management Practices*** | | **2** | |
| Weight for ***Risk Management Practices*** | | **0.1** | |
| Total weighted score for ***Risk Management Practices*** | | **0.067** | |

| **TABLE 3.8**  **TECHNICAL COMPLIANCE SCORING:**  **Total Weighted Scoring for ­Mutshedzi treatment plant (New plant) 79.8 %** | |
| --- | --- |
| **Compliance Criterion** | **Weighted Score** |
| **1. TREATMENT PLANT MEASUREMENTS** | |
| 1A: Design Aspects | **0.1** |
| 1B: Operation Monitoring Practices | **0.138** |
| 1C: Compliance (Final Water Quality) Monitoring Practices | **0.225** |
| 1D: Plant Monitoring Practices | **0.1** |
| 1E: Maintenance Practices | **0.167** |
| 1F: Risk Management Practices | **0.067** |
| TOTAL WEIGHTED SCORE | **1.0** |

**3.5 Technical compliance rating of small water treatment plants**

Based on the Technical Compliance Scoring system used above and the final total weighted score calculated for the specific water treatment plant, the plant can then be rated according to the following Technical Compliance Rating:

| **TABLE 3.9**  **TECHNICAL COMPLIANCE RATING** | |
| --- | --- |
| **Total Weighted Score** | **Rating Description** |
| **0 – 50** | Class 3 Compliance: Total non-compliance; serious and immediate intervention required (TAC) |
| **50 – 90** | Class 2 Compliance: Serious challenges requiring attention and improvement |
| **90 – 100** | Class 1 Compliance: Acceptable compliance |

The following chapter focuses on the corrective actions for non-technical compliance that can be recorded in a drinking water supply.

1. **May 2011**
2. **Mutshedzi Water treatment OLD plant**

| **Table 4.1**  **TECHNICAL COMPLIANCE SCORING** | |
| --- | --- |
| **Criterion** | **Weight** |
| **1. TREATMENT PLANT MEASUREMENTS** | |
| 1A: Design Aspects | **0.1** |
| 1B: Operation Monitoring Practices | **0.2** |
| 1C: Compliance (Final Water Quality) Monitoring Practices | **0.3** |
| 1D: Plant Monitoring Practices | **0.1** |
| 1E: Maintenance Practices | **0.2** |
| 1F: Risk Management Practices | **0.1** |
| **TOTAL** | **1.0** |

| **Table 4.2**  **TECHNICAL COMPLIANCE SCORING:** | | | |
| --- | --- | --- | --- |
| **Criterion** | | **Yes =1** | **No = 0** |
| **1. TREATMENT PLANT MEASUREMENTS** | | | |
| **1A: Design Aspects** | | | |
| Rapid Mixing | Is the mixing intensity sufficient? | 1 |  |
|  | Is the mixing time sufficient? | 1 |  |
|  | Are chemicals dosed at the correct points? | 1 |  |
| Flocculation | Is flocculation intensity at correct levels (not too high; not too low)? | 1 |  |
|  | Is flocculation time sufficient (at least 15 minutes)? | 1 |  |
|  | Can the flocculation channels or tanks be cleaned easily? | 1 |  |
| Sedimentation | Is floc break-up prevented in the inlet to the sedimentation tank? | 1 |  |
|  | Is the upflow velocity in the sedimentation tank sufficiently low? | 1 |  |
|  | Is the weir overflow rate sufficiently low? | 1 |  |
|  | Can the sedimentation tank(s) be desludged easily and effectively? | 1 |  |
| Filtration | Is filtration rate sufficiently low to ensure efficient filtration? | 1 |  |
|  | Is the filtration depth and filter media suitable for the type of raw water? | 1 |  |
|  | Is the underdrain system acceptable to ensure sustainable filtration? | 1 |  |
|  | Is the backwashing system suitable to ensure effective cleaning of sand filters? | 1 |  |
| Disinfection | Can chlorine (or other disinfectant) be dosed at sufficient levels for the whole range of inflow rates and chlorine demands of the raw water (i.e. are dosing pumps sized correctly)? | 1 |  |
|  | Are monitoring systems in place to ensure timeous changing of chlorine cylinders or to replenish disinfectant make-up solutions? | 1 |  |
| Dosing systems | Have dosage systems (coagulation; pH adjustment; oxidation; disinfection; stabilisation) been installed to ensure easy maintenance and trouble-free operation? | 1 |  |
| Maximum possible score for ***Design Aspects*** | | **17** | |
| Total score attained for ***Design Aspects*** | | **17** | |
| Weight for ***Design Aspects*** | | **0.1** | |
| Total weighted score for ***Design Aspects*** | | **0.1** | |

| **Table 4.3**  **TECHNICAL COMPLIANCE SCORING:** | | | |
| --- | --- | --- | --- |
| **Criterion** | | **Yes =1** | **No = 0** |
| **1. TREATMENT PLANT MEASUREMENTS** | | | |
| **1B: Operation Monitoring Practices** | | | |
| Coagulation and Flocculation | Do the process controllers know how much they are dosing (even qualitatively)? | **1** |  |
|  | Can the process controllers adjust the dosage rates and monitor how much is dosed? | **1** |  |
|  | Do they have a programme for monitoring floc formation? |  | **0** |
| Sedimentation | Is the floc blanket observed and dislodging practices based thereon? | **1** |  |
|  | Is the overflow weir kept clean? | **1** |  |
|  | Is the flow of more than one sedimentation tank distributed evenly? | **1** |  |
| Filtration | Is backwashing done properly (at the right time and according to the correct procedure)? | **1** |  |
|  | Is the quality of the filtrate monitored on a regular basis? |  | **0** |
|  | Is excessive head loss development or turbidity breakthrough monitored? |  | **0** |
| Disinfection | Is the chlorine dosed according to previously determined chlorine demand and/or by maintaining acceptable chlorine residual in the final water? |  | **0** |
|  | Is the chlorine residual measured correctly and at the suggested frequency? |  | **0** |
| Stabilisation | Is the stability of the final water determined? | **1** |  |
|  | Is the process controller familiar with the reason for stabilisation and how it can be affected and controlled? | **1** |  |
| **Maximum possible score for *Operation Monitoring Practices*** | | **13** | |
| **Total score attained for *Operation Monitoring Practices*** | | **8** | |
| **Weight for *Operation Monitoring Practices*** | | **0.2** | |
| **Total weighted score for *Operation Monitoring Practices*** | | **0.123** | |

| **Table 4.4**  **TECHNICAL COMPLIANCE SCORING:** | | | |
| --- | --- | --- | --- |
| **Criterion** | | **Yes =1** | **No = 0** |
| **1. TREATMENT PLANT MEASUREMENTS** | | | |
| **1C: Compliance Monitoring Practices** | | | |
| Final water quality | Is the free chlorine residual sufficient? | **1** |  |
|  | Is the turbidity less than 1.0 NTU? |  | **0** |
|  | Is the pH between 6.0 and 9.0? | **1** |  |
|  | Is the colour less than 10 mg/L as Pt? |  | **N/A** |
|  | Is the final water free of any coliform indicator organisms? |  | **0** |
|  | Is the electrical conductivity less than 70 mS/m? |  | **N/A** |
|  | Is the iron level less than 0.2 mg/L as Fe? |  | **N/A** |
|  | Is the aluminium level less than 0.2 mg/L as Al? |  | **N/A** |
| Maximum possible score for ***Compliance Monitoring Practices*** | | **4** | |
| Total score attained for ***Compliance Monitoring Practices*** | | **2** | |
| Weight for ***Compliance Monitoring Practices*** | | **0.3** | |
| Total weighted score for ***Compliance Monitoring Practices*** | | **0.15** | |

| **Table 4.5**  **TECHNICAL COMPLIANCE SCORING:** | | | |
| --- | --- | --- | --- |
| **Criterion** | | **Yes =1** | **No = 0** |
| **1. TREATMENT PLANT MEASUREMENTS** | | | |
| **1D: Plant Monitoring Practices** | | | |
| Plant Infrastructure and Equipment | Are all the pumps in working order? | **1** |  |
|  | Can all the valves open and close properly? | **1** |  |
|  | Are all leaks repaired? | **1** |  |
|  | Are all blockages in pipes cleared? | **1** |  |
|  | Are all unit treatment processes and equipment accessible? | **1** |  |
| Maximum possible score for ***Plant Monitoring Practices*** | | **5** | |
| Total score attained for ***Plant Monitoring Practices*** | | **5** | |
| Weight for ***Plant Monitoring Practices*** | | **0.1** | |
| Total weighted score for ***Plant Monitoring Practices*** | | **0.1** | |

| **Table 4.6**  **TECHNICAL COMPLIANCE SCORING:** | | | |
| --- | --- | --- | --- |
| **Criterion** | | **Yes =1** | **No = 0** |
| **1. TREATMENT PLANT MEASUREMENTS** | | | |
| 1E: Maintenance Practices | | | |
| Overall Treatment Plant | Is back-up service available? | **1** |  |
|  | Is there opportunity for training of the plant personnel in using the sophisticated equipment? | **1** |  |
|  | Are spare-parts readily available? | **1** |  |
|  | Can spare parts be replaced quickly? | **1** |  |
|  | Are spare parts affordable by the municipality? | **1** |  |
|  | Is back-up service readily available? | **1** |  |
|  | Can service providers be on site at short notice? | **1** |  |
|  | Is this service reliable? | **1** |  |
|  | Can the community do certain parts of the maintenance themselves? |  | **N/A** |
|  | Are there adequate communication facilities between the plant and management, authorities, service providers, suppliers and consultants? | **1** |  |
|  | Can this be readily improved? | **1** |  |
|  | Can the plant personnel or persons in the community perform their own maintenance (or parts thereof)? |  | **N/A** |
|  | Can they be trained and are such training facilities available? | **1** |  |
|  | Is there good access to the plant by motor vehicles and delivery trucks? | **1** |  |
|  | Can the existing access routes be improved with existing funds? | **1** |  |
|  | Are funds available? | **1** |  |
|  | Is there possibility of alternative sources of funding for maintenance? | **1** |  |
| Maximum possible score for ***Maintenance Practices*** | | **18** | |
| Total score attained for ***Maintenance Practices*** | | **15** | |
| Weight for ***Maintenance Practices*** | | **0.2** | |
| Total weighted score for ***Maintenance Practices*** | | **0.167** | |

| **Table 4.7**  **TECHNICAL COMPLIANCE SCORING:** | | | |
| --- | --- | --- | --- |
| **Criterion** | | **Yes =1** | **No = 0** |
| **1. TREATMENT PLANT MEASUREMENTS** | | | |
| **1F: Risk Management Practices** | | | |
| Source to Tap | Is there a risk management system in place? | **1** |  |
|  | Are risk reduction options drawn up and implemented based on the risk assessment? | **1** |  |
|  | Are the risk assessments carried out on a regular basis? |  | **0** |
| Maximum possible score for ***Risk Management Practices*** | | **3** | |
| Total score attained for ***Risk Management Practices*** | | **2** | |
| Weight for ***Risk Management Practices*** | | **0.1** | |
| Total weighted score for ***Risk Management Practices*** | | **0.067** | |

| **TABLE 4.8**  **TECHNICAL COMPLIANCE SCORING:**  **Total Weighted Scoring for Mutshedzi Water Treatment Plant 66.8 %** | |
| --- | --- |
| **Compliance Criterion** | **Weighted Score** |
| **1. TREATMENT PLANT MEASUREMENTS** | |
| 1A: Design Aspects | **0.1** |
| 1B: Operation Monitoring Practices | **0.123** |
| 1C: Compliance (Final Water Quality) Monitoring Practices | **0.15** |
| 1D: Plant Monitoring Practices | **0.1** |
| 1E: Maintenance Practices | **0.167** |
| 1F: Risk Management Practices | **0.067** |
| TOTAL WEIGHTED SCORE | **1.0** |

**3.5 Technical compliance rating of small water treatment plants**

Based on the Technical Compliance Scoring system used above and the final total weighted score calculated for the specific water treatment plant, the plant can then be rated according to the following Technical Compliance Rating:

| **TABLE 4.9**  **TECHNICAL COMPLIANCE RATING** | |
| --- | --- |
| **Total Weighted Score** | **Rating Description** |
| **0 – 50** | Class 3 Compliance: Total non-compliance; serious and immediate intervention required (TAC) |
| **50 – 90** | Class 2 Compliance: Serious challenges requiring attention and improvement |
| **90 – 100** | Class 1 Compliance: Acceptable compliance |

The following chapter focuses on the corrective actions for non-technical compliance that can be recorded in a drinking water supply.

1. **May 2011**
2. **Mutale regional Water treatment new plant**

| **Table 5.1**  **TECHNICAL COMPLIANCE SCORING** | |
| --- | --- |
| **Criterion** | **Weight** |
| **1. TREATMENT PLANT MEASUREMENTS** | |
| 1A: Design Aspects | **0.1** |
| 1B: Operation Monitoring Practices | **0.2** |
| 1C: Compliance (Final Water Quality) Monitoring Practices | **0.3** |
| 1D: Plant Monitoring Practices | **0.1** |
| 1E: Maintenance Practices | **0.2** |
| 1F: Risk Management Practices | **0.1** |
| **TOTAL** | **1.0** |

| **Table 5.2**  **TECHNICAL COMPLIANCE SCORING:** | | | |
| --- | --- | --- | --- |
| **Criterion** | | **Yes =1** | **No = 0** |
| **1. TREATMENT PLANT MEASUREMENTS** | | | |
| **1A: Design Aspects** | | | |
| Rapid Mixing | Is the mixing intensity sufficient? | 1 |  |
|  | Is the mixing time sufficient? | 1 |  |
|  | Are chemicals dosed at the correct points? | 1 |  |
| Flocculation | Is flocculation intensity at correct levels (not too high; not too low)? | 1 |  |
|  | Is flocculation time sufficient (at least 15 minutes)? | 1 |  |
|  | Can the flocculation channels or tanks be cleaned easily? | 1 |  |
| Sedimentation | Is floc break-up prevented in the inlet to the sedimentation tank? | 1 |  |
|  | Is the upflow velocity in the sedimentation tank sufficiently low? | 1 |  |
|  | Is the weir overflow rate sufficiently low? | 1 |  |
|  | Can the sedimentation tank(s) be desludged easily and effectively? | 1 |  |
| Filtration | Is filtration rate sufficiently low to ensure efficient filtration? | 1 |  |
|  | Is the filtration depth and filter media suitable for the type of raw water? | 1 |  |
|  | Is the underdrain system acceptable to ensure sustainable filtration? | 1 |  |
|  | Is the backwashing system suitable to ensure effective cleaning of sand filters? | 1 |  |
| Disinfection | Can chlorine (or other disinfectant) be dosed at sufficient levels for the whole range of inflow rates and chlorine demands of the raw water (i.e. are dosing pumps sized correctly)? | 1 |  |
|  | Are monitoring systems in place to ensure timeous changing of chlorine cylinders or to replenish disinfectant make-up solutions? | 1 |  |
| Dosing systems | Have dosage systems (coagulation; pH adjustment; oxidation; disinfection; stabilisation) been installed to ensure easy maintenance and trouble-free operation? | 1 |  |
| Maximum possible score for ***Design Aspects*** | | **17** | |
| Total score attained for ***Design Aspects*** | | **17** | |
| Weight for ***Design Aspects*** | | **0.1** | |
| Total weighted score for ***Design Aspects*** | | **0.1** | |

| **Table 5.3**  **TECHNICAL COMPLIANCE SCORING:** | | | |
| --- | --- | --- | --- |
| **Criterion** | | **Yes =1** | **No = 0** |
| **1. TREATMENT PLANT MEASUREMENTS** | | | |
| **1B: Operation Monitoring Practices** | | | |
| Coagulation and Flocculation | Do the process controllers know how much they are dosing (even qualitatively)? | **1** |  |
|  | Can the process controllers adjust the dosage rates and monitor how much is dosed? | **1** |  |
|  | Do they have a programme for monitoring floc formation? |  | **0** |
| Sedimentation | Is the floc blanket observed and dislodging practices based thereon? | **1** |  |
|  | Is the overflow weir kept clean? | **1** |  |
|  | Is the flow of more than one sedimentation tank distributed evenly? | **1** |  |
| Filtration | Is backwashing done properly (at the right time and according to the correct procedure)? | **1** |  |
|  | Is the quality of the filtrate monitored on a regular basis? |  | **0** |
|  | Is excessive head loss development or turbidity breakthrough monitored? |  | **0** |
| Disinfection | Is the chlorine dosed according to previously determined chlorine demand and/or by maintaining acceptable chlorine residual in the final water? | **1** |  |
|  | Is the chlorine residual measured correctly and at the suggested frequency? | **1** |  |
| Stabilisation | Is the stability of the final water determined? | **1** |  |
|  | Is the process controller familiar with the reason for stabilisation and how it can be affected and controlled? | **1** |  |
| **Maximum possible score for *Operation Monitoring Practices*** | | **13** | |
| **Total score attained for *Operation Monitoring Practices*** | | **10** | |
| **Weight for *Operation Monitoring Practices*** | | **0.2** | |
| **Total weighted score for *Operation Monitoring Practices*** | | **0.154** | |

| **Table 5.4**  **TECHNICAL COMPLIANCE SCORING:** | | | |
| --- | --- | --- | --- |
| **Criterion** | | **Yes =1** | **No = 0** |
| **1. TREATMENT PLANT MEASUREMENTS** | | | |
| **1C: Compliance Monitoring Practices** | | | |
| Final water quality | Is the free chlorine residual sufficient? | **1** |  |
|  | Is the turbidity less than 1.0 NTU? |  | **0** |
|  | Is the pH between 6.0 and 9.0? | **1** |  |
|  | Is the colour less than 10 mg/L as Pt? |  | **N/A** |
|  | Is the final water free of any coliform indicator organisms? |  | **0** |
|  | Is the electrical conductivity less than 70 mS/m? |  | **N/A** |
|  | Is the iron level less than 0.2 mg/L as Fe? |  | **N/A** |
|  | Is the aluminium level less than 0.2 mg/L as Al? |  | **N/A** |
| Maximum possible score for ***Compliance Monitoring Practices*** | | **4** | |
| Total score attained for ***Compliance Monitoring Practices*** | | **2** | |
| Weight for ***Compliance Monitoring Practices*** | | **0.3** | |
| Total weighted score for ***Compliance Monitoring Practices*** | | **0.15** | |

| **Table 5.5**  **TECHNICAL COMPLIANCE SCORING:** | | | |
| --- | --- | --- | --- |
| **Criterion** | | **Yes =1** | **No = 0** |
| **1. TREATMENT PLANT MEASUREMENTS** | | | |
| **1D: Plant Monitoring Practices** | | | |
| Plant Infrastructure and Equipment | Are all the pumps in working order? | **1** |  |
|  | Can all the valves open and close properly? | **1** |  |
|  | Are all leaks repaired? |  | **0** |
|  | Are all blockages in pipes cleared? | **1** |  |
|  | Are all unit treatment processes and equipment accessible? | **1** |  |
| Maximum possible score for ***Plant Monitoring Practices*** | | **5** | |
| Total score attained for ***Plant Monitoring Practices*** | | **5** | |
| Weight for ***Plant Monitoring Practices*** | | **0.1** | |
| Total weighted score for ***Plant Monitoring Practices*** | | **0.1** | |

| **Table 5.6**  **TECHNICAL COMPLIANCE SCORING:** | | | |
| --- | --- | --- | --- |
| **Criterion** | | **Yes =1** | **No = 0** |
| **1. TREATMENT PLANT MEASUREMENTS** | | | |
| 1E: Maintenance Practices | | | |
| Overall Treatment Plant | Is back-up service available? | **1** |  |
|  | Is there opportunity for training of the plant personnel in using the sophisticated equipment? | **1** |  |
|  | Are spare-parts readily available? | **1** |  |
|  | Can spare parts be replaced quickly? | **1** |  |
|  | Are spare parts affordable by the municipality? | **1** |  |
|  | Is back-up service readily available? | **1** |  |
|  | Can service providers be on site at short notice? | **1** |  |
|  | Is this service reliable? | **1** |  |
|  | Can the community do certain parts of the maintenance themselves? |  | **N/A** |
|  | Are there adequate communication facilities between the plant and management, authorities, service providers, suppliers and consultants? | **1** |  |
|  | Can this be readily improved? | **1** |  |
|  | Can the plant personnel or persons in the community perform their own maintenance (or parts thereof)? |  | **N/A** |
|  | Can they be trained and are such training facilities available? | **1** |  |
|  | Is there good access to the plant by motor vehicles and delivery trucks? | **1** |  |
|  | Can the existing access routes be improved with existing funds? | **1** |  |
|  | Are funds available? | **1** |  |
|  | Is there possibility of alternative sources of funding for maintenance? | **1** |  |
| Maximum possible score for ***Maintenance Practices*** | | **18** | |
| Total score attained for ***Maintenance Practices*** | | **15** | |
| Weight for ***Maintenance Practices*** | | **0.2** | |
| Total weighted score for ***Maintenance Practices*** | | **0.167** | |

| **Table 5.7**  **TECHNICAL COMPLIANCE SCORING:** | | | |
| --- | --- | --- | --- |
| **Criterion** | | **Yes =1** | **No = 0** |
| **1. TREATMENT PLANT MEASUREMENTS** | | | |
| **1F: Risk Management Practices** | | | |
| Source to Tap | Is there a risk management system in place? | **1** |  |
|  | Are risk reduction options drawn up and implemented based on the risk assessment? | **1** |  |
|  | Are the risk assessments carried out on a regular basis? |  | **0** |
| Maximum possible score for ***Risk Management Practices*** | | **3** | |
| Total score attained for ***Risk Management Practices*** | | **2** | |
| Weight for ***Risk Management Practices*** | | **0.1** | |
| Total weighted score for ***Risk Management Practices*** | | **0.067** | |

| **TABLE 5.8**  **TECHNICAL COMPLIANCE SCORING:**  **Total Weighted Scoring for Mutshedzi Water Treatment Plant 73.7 %** | |
| --- | --- |
| **Compliance Criterion** | **Weighted Score** |
| **1. TREATMENT PLANT MEASUREMENTS** | |
| 1A: Design Aspects | **0.1** |
| 1B: Operation Monitoring Practices | **0.154** |
| 1C: Compliance (Final Water Quality) Monitoring Practices | **0.15** |
| 1D: Plant Monitoring Practices | **0.1** |
| 1E: Maintenance Practices | **0.167** |
| 1F: Risk Management Practices | **0.067** |
| TOTAL WEIGHTED SCORE | **1.0** |

**3.5 Technical compliance rating of small water treatment plants**

Based on the Technical Compliance Scoring system used above and the final total weighted score calculated for the specific water treatment plant, the plant can then be rated according to the following Technical Compliance Rating:

| **TABLE 5.9**  **TECHNICAL COMPLIANCE RATING** | |
| --- | --- |
| **Total Weighted Score** | **Rating Description** |
| **0 – 50** | Class 3 Compliance: Total non-compliance; serious and immediate intervention required (TAC) |
| **50 – 90** | Class 2 Compliance: Serious challenges requiring attention and improvement |
| **90 – 100** | Class 1 Compliance: Acceptable compliance |

The following chapter focuses on the corrective actions for non-technical compliance that can be recorded in a drinking water supply.

1. **May 2011**
2. **Mutale regional Water treatment OLD plant**

| **Table 6.1**  **TECHNICAL COMPLIANCE SCORING** | |
| --- | --- |
| **Criterion** | **Weight** |
| **1. TREATMENT PLANT MEASUREMENTS** | |
| 1A: Design Aspects | **0.1** |
| 1B: Operation Monitoring Practices | **0.2** |
| 1C: Compliance (Final Water Quality) Monitoring Practices | **0.3** |
| 1D: Plant Monitoring Practices | **0.1** |
| 1E: Maintenance Practices | **0.2** |
| 1F: Risk Management Practices | **0.1** |
| **TOTAL** | **1.0** |

| **Table 6.2**  **TECHNICAL COMPLIANCE SCORING:** | | | |
| --- | --- | --- | --- |
| **Criterion** | | **Yes =1** | **No = 0** |
| **1. TREATMENT PLANT MEASUREMENTS** | | | |
| **1A: Design Aspects** | | | |
| Rapid Mixing | Is the mixing intensity sufficient? |  | 0 |
|  | Is the mixing time sufficient? |  | 0 |
|  | Are chemicals dosed at the correct points? |  | 0 |
| Flocculation | Is flocculation intensity at correct levels (not too high; not too low)? |  | 0 |
|  | Is flocculation time sufficient (at least 15 minutes)? |  | 0 |
|  | Can the flocculation channels or tanks be cleaned easily? | 1 |  |
| Sedimentation | Is floc break-up prevented in the inlet to the sedimentation tank? |  | 0 |
|  | Is the upflow velocity in the sedimentation tank sufficiently low? | 1 |  |
|  | Is the weir overflow rate sufficiently low? | 1 |  |
|  | Can the sedimentation tank(s) be desludged easily and effectively? | 1 |  |
| Filtration | Is filtration rate sufficiently low to ensure efficient filtration? | 1 |  |
|  | Is the filtration depth and filter media suitable for the type of raw water? | 1 |  |
|  | Is the underdrain system acceptable to ensure sustainable filtration? | 1 |  |
|  | Is the backwashing system suitable to ensure effective cleaning of sand filters? | 1 |  |
| Disinfection | Can chlorine (or other disinfectant) be dosed at sufficient levels for the whole range of inflow rates and chlorine demands of the raw water (i.e. are dosing pumps sized correctly)? | 1 |  |
|  | Are monitoring systems in place to ensure timeous changing of chlorine cylinders or to replenish disinfectant make-up solutions? | 1 |  |
| Dosing systems | Have dosage systems (coagulation; pH adjustment; oxidation; disinfection; stabilisation) been installed to ensure easy maintenance and trouble-free operation? | 1 |  |
| Maximum possible score for ***Design Aspects*** | | **17** | |
| Total score attained for ***Design Aspects*** | | **17** | |
| Weight for ***Design Aspects*** | | **0.1** | |
| Total weighted score for ***Design Aspects*** | | **0.1** | |

| **Table 6.3**  **TECHNICAL COMPLIANCE SCORING:** | | | |
| --- | --- | --- | --- |
| **Criterion** | | **Yes =1** | **No = 0** |
| **1. TREATMENT PLANT MEASUREMENTS** | | | |
| **1B: Operation Monitoring Practices** | | | |
| Coagulation and Flocculation | Do the process controllers know how much they are dosing (even qualitatively)? | **1** |  |
|  | Can the process controllers adjust the dosage rates and monitor how much is dosed? | **1** |  |
|  | Do they have a programme for monitoring floc formation? |  | **0** |
| Sedimentation | Is the floc blanket observed and dislodging practices based thereon? | **1** |  |
|  | Is the overflow weir kept clean? | **1** |  |
|  | Is the flow of more than one sedimentation tank distributed evenly? | **1** |  |
| Filtration | Is backwashing done properly (at the right time and according to the correct procedure)? | **1** |  |
|  | Is the quality of the filtrate monitored on a regular basis? |  | **0** |
|  | Is excessive head loss development or turbidity breakthrough monitored? |  | **0** |
| Disinfection | Is the chlorine dosed according to previously determined chlorine demand and/or by maintaining acceptable chlorine residual in the final water? | **1** |  |
|  | Is the chlorine residual measured correctly and at the suggested frequency? | **1** |  |
| Stabilisation | Is the stability of the final water determined? | **1** |  |
|  | Is the process controller familiar with the reason for stabilisation and how it can be affected and controlled? | **1** |  |
| **Maximum possible score for *Operation Monitoring Practices*** | | **13** | |
| **Total score attained for *Operation Monitoring Practices*** | | **10** | |
| **Weight for *Operation Monitoring Practices*** | | **0.2** | |
| **Total weighted score for *Operation Monitoring Practices*** | | **0.154** | |

| **Table 6.4**  **TECHNICAL COMPLIANCE SCORING:** | | | |
| --- | --- | --- | --- |
| **Criterion** | | **Yes =1** | **No = 0** |
| **1. TREATMENT PLANT MEASUREMENTS** | | | |
| **1C: Compliance Monitoring Practices** | | | |
| Final water quality | Is the free chlorine residual sufficient? | **1** |  |
|  | Is the turbidity less than 1.0 NTU? |  | **0** |
|  | Is the pH between 6.0 and 9.0? | **1** |  |
|  | Is the colour less than 10 mg/L as Pt? |  | **N/A** |
|  | Is the final water free of any coliform indicator organisms? |  | **0** |
|  | Is the electrical conductivity less than 70 mS/m? |  | **N/A** |
|  | Is the iron level less than 0.2 mg/L as Fe? |  | **N/A** |
|  | Is the aluminium level less than 0.2 mg/L as Al? |  | **N/A** |
| Maximum possible score for ***Compliance Monitoring Practices*** | | **4** | |
| Total score attained for ***Compliance Monitoring Practices*** | | **2** | |
| Weight for ***Compliance Monitoring Practices*** | | **0.3** | |
| Total weighted score for ***Compliance Monitoring Practices*** | | **0.15** | |

| **Table 6.5**  **TECHNICAL COMPLIANCE SCORING:** | | | |
| --- | --- | --- | --- |
| **Criterion** | | **Yes =1** | **No = 0** |
| **1. TREATMENT PLANT MEASUREMENTS** | | | |
| **1D: Plant Monitoring Practices** | | | |
| Plant Infrastructure and Equipment | Are all the pumps in working order? | **1** |  |
|  | Can all the valves open and close properly? | **1** |  |
|  | Are all leaks repaired? |  | **0** |
|  | Are all blockages in pipes cleared? | **1** |  |
|  | Are all unit treatment processes and equipment accessible? | **1** |  |
| Maximum possible score for ***Plant Monitoring Practices*** | | **5** | |
| Total score attained for ***Plant Monitoring Practices*** | | **5** | |
| Weight for ***Plant Monitoring Practices*** | | **0.1** | |
| Total weighted score for ***Plant Monitoring Practices*** | | **0.1** | |

| **Table 6.6**  **TECHNICAL COMPLIANCE SCORING:** | | | |
| --- | --- | --- | --- |
| **Criterion** | | **Yes =1** | **No = 0** |
| **1. TREATMENT PLANT MEASUREMENTS** | | | |
| 1E: Maintenance Practices | | | |
| Overall Treatment Plant | Is back-up service available? | **1** |  |
|  | Is there opportunity for training of the plant personnel in using the sophisticated equipment? | **1** |  |
|  | Are spare-parts readily available? | **1** |  |
|  | Can spare parts be replaced quickly? | **1** |  |
|  | Are spare parts affordable by the municipality? | **1** |  |
|  | Is back-up service readily available? | **1** |  |
|  | Can service providers be on site at short notice? | **1** |  |
|  | Is this service reliable? | **1** |  |
|  | Can the community do certain parts of the maintenance themselves? |  | **N/A** |
|  | Are there adequate communication facilities between the plant and management, authorities, service providers, suppliers and consultants? | **1** |  |
|  | Can this be readily improved? | **1** |  |
|  | Can the plant personnel or persons in the community perform their own maintenance (or parts thereof)? |  | **N/A** |
|  | Can they be trained and are such training facilities available? | **1** |  |
|  | Is there good access to the plant by motor vehicles and delivery trucks? | **1** |  |
|  | Can the existing access routes be improved with existing funds? | **1** |  |
|  | Are funds available? | **1** |  |
|  | Is there possibility of alternative sources of funding for maintenance? | **1** |  |
| Maximum possible score for ***Maintenance Practices*** | | **18** | |
| Total score attained for ***Maintenance Practices*** | | **15** | |
| Weight for ***Maintenance Practices*** | | **0.2** | |
| Total weighted score for ***Maintenance Practices*** | | **0.167** | |

| **Table 6.7**  **TECHNICAL COMPLIANCE SCORING:** | | | |
| --- | --- | --- | --- |
| **Criterion** | | **Yes =1** | **No = 0** |
| **1. TREATMENT PLANT MEASUREMENTS** | | | |
| **1F: Risk Management Practices** | | | |
| Source to Tap | Is there a risk management system in place? | **1** |  |
|  | Are risk reduction options drawn up and implemented based on the risk assessment? | **1** |  |
|  | Are the risk assessments carried out on a regular basis? |  | **0** |
| Maximum possible score for ***Risk Management Practices*** | | **3** | |
| Total score attained for ***Risk Management Practices*** | | **2** | |
| Weight for ***Risk Management Practices*** | | **0.1** | |
| Total weighted score for ***Risk Management Practices*** | | **0.067** | |

| **TABLE 6.8**  **TECHNICAL COMPLIANCE SCORING:**  **Total Weighted Scoring for Mutshedzi Water Treatment Plant 73.7 %** | |
| --- | --- |
| **Compliance Criterion** | **Weighted Score** |
| **1. TREATMENT PLANT MEASUREMENTS** | |
| 1A: Design Aspects | **0.1** |
| 1B: Operation Monitoring Practices | **0.154** |
| 1C: Compliance (Final Water Quality) Monitoring Practices | **0.15** |
| 1D: Plant Monitoring Practices | **0.1** |
| 1E: Maintenance Practices | **0.167** |
| 1F: Risk Management Practices | **0.067** |
| TOTAL WEIGHTED SCORE | **1.0** |

**3.5 Technical compliance rating of small water treatment plants**

Based on the Technical Compliance Scoring system used above and the final total weighted score calculated for the specific water treatment plant, the plant can then be rated according to the following Technical Compliance Rating:

| **TABLE 6.9**  **TECHNICAL COMPLIANCE RATING** | |
| --- | --- |
| **Total Weighted Score** | **Rating Description** |
| **0 – 50** | Class 3 Compliance: Total non-compliance; serious and immediate intervention required (TAC) |
| **50 – 90** | Class 2 Compliance: Serious challenges requiring attention and improvement |
| **90 – 100** | Class 1 Compliance: Acceptable compliance |

The following chapter focuses on the corrective actions for non-technical compliance that can be recorded in a drinking water supply.

1. **May 2011**
2. **TSHEDZA PACKAGE PLANT**

| **Table 7.1**  **TECHNICAL COMPLIANCE SCORING** | |
| --- | --- |
| **Criterion** | **Weight** |
| **1. TREATMENT PLANT MEASUREMENTS** | |
| 1A: Design Aspects | **0.1** |
| 1B: Operation Monitoring Practices | **0.2** |
| 1C: Compliance (Final Water Quality) Monitoring Practices | **0.3** |
| 1D: Plant Monitoring Practices | **0.1** |
| 1E: Maintenance Practices | **0.2** |
| 1F: Risk Management Practices | **0.1** |
| **TOTAL** | **1.0** |

| **Table 7.2**  **TECHNICAL COMPLIANCE SCORING:** | | | |
| --- | --- | --- | --- |
| **Criterion** | | **Yes =1** | **No = 0** |
| **1. TREATMENT PLANT MEASUREMENTS** | | | |
| **1A: Design Aspects** | | | |
| Rapid Mixing | Is the mixing intensity sufficient? | 1 |  |
|  | Is the mixing time sufficient? | 1 |  |
|  | Are chemicals dosed at the correct points? | 1 |  |
| Flocculation | Is flocculation intensity at correct levels (not too high; not too low)? |  | N/A |
|  | Is flocculation time sufficient (at least 15 minutes)? |  | N/A |
|  | Can the flocculation channels or tanks be cleaned easily? |  | N/A |
| Sedimentation | Is floc break-up prevented in the inlet to the sedimentation tank? |  | N/A |
|  | Is the upflow velocity in the sedimentation tank sufficiently low? |  | N/A |
|  | Is the weir overflow rate sufficiently low? |  | N/A |
|  | Can the sedimentation tank(s) be desludged easily and effectively? |  | N/A |
| Filtration | Is filtration rate sufficiently low to ensure efficient filtration? | 1 |  |
|  | Is the filtration depth and filter media suitable for the type of raw water? | 1 |  |
|  | Is the underdrain system acceptable to ensure sustainable filtration? | 1 |  |
|  | Is the backwashing system suitable to ensure effective cleaning of sand filters? | 1 |  |
| Disinfection | Can chlorine (or other disinfectant) be dosed at sufficient levels for the whole range of inflow rates and chlorine demands of the raw water (i.e. are dosing pumps sized correctly)? | 1 |  |
|  | Are monitoring systems in place to ensure timeous changing of chlorine cylinders or to replenish disinfectant make-up solutions? | 1 |  |
| Dosing systems | Have dosage systems (coagulation; pH adjustment; oxidation; disinfection; stabilisation) been installed to ensure easy maintenance and trouble-free operation? | 1 |  |
| Maximum possible score for ***Design Aspects*** | | **10** | |
| Total score attained for ***Design Aspects*** | | **10** | |
| Weight for ***Design Aspects*** | | **0.1** | |
| Total weighted score for ***Design Aspects*** | | **0.1** | |

| **Table 7.3**  **TECHNICAL COMPLIANCE SCORING:** | | | |
| --- | --- | --- | --- |
| **Criterion** | | **Yes =1** | **No = 0** |
| **1. TREATMENT PLANT MEASUREMENTS** | | | |
| **1B: Operation Monitoring Practices** | | | |
| Coagulation and Flocculation | Do the process controllers know how much they are dosing (even qualitatively)? | **1** |  |
|  | Can the process controllers adjust the dosage rates and monitor how much is dosed? | **1** |  |
|  | Do they have a programme for monitoring floc formation? |  | N/A |
| Sedimentation | Is the floc blanket observed and dislodging practices based thereon? |  | N/A |
|  | Is the overflow weir kept clean? |  | N/A |
|  | Is the flow of more than one sedimentation tank distributed evenly? |  | N/A |
| Filtration | Is backwashing done properly (at the right time and according to the correct procedure)? | **1** |  |
|  | Is the quality of the filtrate monitored on a regular basis? |  | N/A |
|  | Is excessive head loss development or turbidity breakthrough monitored? |  | N/A |
| Disinfection | Is the chlorine dosed according to previously determined chlorine demand and/or by maintaining acceptable chlorine residual in the final water? | **1** |  |
|  | Is the chlorine residual measured correctly and at the suggested frequency? | **1** |  |
| Stabilisation | Is the stability of the final water determined? | **1** |  |
|  | Is the process controller familiar with the reason for stabilisation and how it can be affected and controlled? | **1** |  |
| **Maximum possible score for *Operation Monitoring Practices*** | | **7** | |
| **Total score attained for *Operation Monitoring Practices*** | | **7** | |
| **Weight for *Operation Monitoring Practices*** | | **0.2** | |
| **Total weighted score for *Operation Monitoring Practices*** | | **0.2** | |

| **Table 7.4**  **TECHNICAL COMPLIANCE SCORING:** | | | |
| --- | --- | --- | --- |
| **Criterion** | | **Yes =1** | **No = 0** |
| **1. TREATMENT PLANT MEASUREMENTS** | | | |
| **1C: Compliance Monitoring Practices** | | | |
| Final water quality | Is the free chlorine residual sufficient? | **1** |  |
|  | Is the turbidity less than 1.0 NTU? | **1** |  |
|  | Is the pH between 6.0 and 9.0? | **1** |  |
|  | Is the colour less than 10 mg/L as Pt? |  | **N/A** |
|  | Is the final water free of any coliform indicator organisms? | **1** |  |
|  | Is the electrical conductivity less than 70 mS/m? |  | **N/A** |
|  | Is the iron level less than 0.2 mg/L as Fe? |  | **N/A** |
|  | Is the aluminium level less than 0.2 mg/L as Al? |  | **N/A** |
| Maximum possible score for ***Compliance Monitoring Practices*** | | **4** | |
| Total score attained for ***Compliance Monitoring Practices*** | | **4** | |
| Weight for ***Compliance Monitoring Practices*** | | **0.3** | |
| Total weighted score for ***Compliance Monitoring Practices*** | | **0.3** | |

| **Table 7.5**  **TECHNICAL COMPLIANCE SCORING:** | | | |
| --- | --- | --- | --- |
| **Criterion** | | **Yes =1** | **No = 0** |
| **1. TREATMENT PLANT MEASUREMENTS** | | | |
| **1D: Plant Monitoring Practices** | | | |
| Plant Infrastructure and Equipment | Are all the pumps in working order? | **1** |  |
|  | Can all the valves open and close properly? | **1** |  |
|  | Are all leaks repaired? | **1** |  |
|  | Are all blockages in pipes cleared? | **1** |  |
|  | Are all unit treatment processes and equipment accessible? | **1** |  |
| Maximum possible score for ***Plant Monitoring Practices*** | | **5** | |
| Total score attained for ***Plant Monitoring Practices*** | | **5** | |
| Weight for ***Plant Monitoring Practices*** | | **0.1** | |
| Total weighted score for ***Plant Monitoring Practices*** | | **0.1** | |

| **Table 7.6**  **TECHNICAL COMPLIANCE SCORING:** | | | |
| --- | --- | --- | --- |
| **Criterion** | | **Yes =1** | **No = 0** |
| **1. TREATMENT PLANT MEASUREMENTS** | | | |
| 1E: Maintenance Practices | | | |
| Overall Treatment Plant | Is back-up service available? | **1** |  |
|  | Is there opportunity for training of the plant personnel in using the sophisticated equipment? | **1** |  |
|  | Are spare-parts readily available? | **1** |  |
|  | Can spare parts be replaced quickly? | **1** |  |
|  | Are spare parts affordable by the municipality? | **1** |  |
|  | Is back-up service readily available? | **1** |  |
|  | Can service providers be on site at short notice? | **1** |  |
|  | Is this service reliable? | **1** |  |
|  | Can the community do certain parts of the maintenance themselves? |  | **N/A** |
|  | Are there adequate communication facilities between the plant and management, authorities, service providers, suppliers and consultants? | **1** |  |
|  | Can this be readily improved? | **1** |  |
|  | Can the plant personnel or persons in the community perform their own maintenance (or parts thereof)? |  | **N/A** |
|  | Can they be trained and are such training facilities available? | **1** |  |
|  | Is there good access to the plant by motor vehicles and delivery trucks? | **1** |  |
|  | Can the existing access routes be improved with existing funds? | **1** |  |
|  | Are funds available? | **1** |  |
|  | Is there possibility of alternative sources of funding for maintenance? | **1** |  |
| Maximum possible score for ***Maintenance Practices*** | | **15** | |
| Total score attained for ***Maintenance Practices*** | | **15** | |
| Weight for ***Maintenance Practices*** | | **0.2** | |
| Total weighted score for ***Maintenance Practices*** | | **0.2** | |

| **Table 7.7**  **TECHNICAL COMPLIANCE SCORING:** | | | |
| --- | --- | --- | --- |
| **Criterion** | | **Yes =1** | **No = 0** |
| **1. TREATMENT PLANT MEASUREMENTS** | | | |
| **1F: Risk Management Practices** | | | |
| Source to Tap | Is there a risk management system in place? | **1** |  |
|  | Are risk reduction options drawn up and implemented based on the risk assessment? | **1** |  |
|  | Are the risk assessments carried out on a regular basis? |  | **0** |
| Maximum possible score for ***Risk Management Practices*** | | **3** | |
| Total score attained for ***Risk Management Practices*** | | **2** | |
| Weight for ***Risk Management Practices*** | | **0.1** | |
| Total weighted score for ***Risk Management Practices*** | | **0.067** | |

| **TABLE 7.8**  **TECHNICAL COMPLIANCE SCORING:**  **Total Weighted Scoring Tshedza package plant 96.7 %** | |
| --- | --- |
| **Compliance Criterion** | **Weighted Score** |
| **1. TREATMENT PLANT MEASUREMENTS** | |
| 1A: Design Aspects | **0.1** |
| 1B: Operation Monitoring Practices | **0.2** |
| 1C: Compliance (Final Water Quality) Monitoring Practices | **0.3** |
| 1D: Plant Monitoring Practices | **0.1** |
| 1E: Maintenance Practices | **0.2** |
| 1F: Risk Management Practices | **0.067** |
| TOTAL WEIGHTED SCORE | **1.0** |

**3.5 Technical compliance rating of small water treatment plants**

Based on the Technical Compliance Scoring system used above and the final total weighted score calculated for the specific water treatment plant, the plant can then be rated according to the following Technical Compliance Rating:

| **TABLE 7.9**  **TECHNICAL COMPLIANCE RATING** | |
| --- | --- |
| **Total Weighted Score** | **Rating Description** |
| **0 – 50** | Class 3 Compliance: Total non-compliance; serious and immediate intervention required (TAC) |
| **50 – 90** | Class 2 Compliance: Serious challenges requiring attention and improvement |
| **90 – 100** | Class 1 Compliance: Acceptable compliance |

The following chapter focuses on the corrective actions for non-technical compliance that can be recorded in a drinking water supply.

1. **May 2011**
2. **DZINGAHE PACKAGE PLANT**

| **Table 8.1**  **TECHNICAL COMPLIANCE SCORING** | |
| --- | --- |
| **Criterion** | **Weight** |
| **1. TREATMENT PLANT MEASUREMENTS** | |
| 1A: Design Aspects | **0.1** |
| 1B: Operation Monitoring Practices | **0.2** |
| 1C: Compliance (Final Water Quality) Monitoring Practices | **0.3** |
| 1D: Plant Monitoring Practices | **0.1** |
| 1E: Maintenance Practices | **0.2** |
| 1F: Risk Management Practices | **0.1** |
| **TOTAL** | **1.0** |

| **Table 8.2**  **TECHNICAL COMPLIANCE SCORING:** | | | |
| --- | --- | --- | --- |
| **Criterion** | | **Yes =1** | **No = 0** |
| **1. TREATMENT PLANT MEASUREMENTS** | | | |
| **1A: Design Aspects** | | | |
| Rapid Mixing | Is the mixing intensity sufficient? | 1 |  |
|  | Is the mixing time sufficient? | 1 |  |
|  | Are chemicals dosed at the correct points? | 1 |  |
| Flocculation | Is flocculation intensity at correct levels (not too high; not too low)? |  | N/A |
|  | Is flocculation time sufficient (at least 15 minutes)? |  | N/A |
|  | Can the flocculation channels or tanks be cleaned easily? |  | N/A |
| Sedimentation | Is floc break-up prevented in the inlet to the sedimentation tank? |  | N/A |
|  | Is the upflow velocity in the sedimentation tank sufficiently low? |  | N/A |
|  | Is the weir overflow rate sufficiently low? |  | N/A |
|  | Can the sedimentation tank(s) be desludged easily and effectively? |  | N/A |
| Filtration | Is filtration rate sufficiently low to ensure efficient filtration? | 1 |  |
|  | Is the filtration depth and filter media suitable for the type of raw water? | 1 |  |
|  | Is the underdrain system acceptable to ensure sustainable filtration? | 1 |  |
|  | Is the backwashing system suitable to ensure effective cleaning of sand filters? | 1 |  |
| Disinfection | Can chlorine (or other disinfectant) be dosed at sufficient levels for the whole range of inflow rates and chlorine demands of the raw water (i.e. are dosing pumps sized correctly)? | 1 |  |
|  | Are monitoring systems in place to ensure timeous changing of chlorine cylinders or to replenish disinfectant make-up solutions? | 1 |  |
| Dosing systems | Have dosage systems (coagulation; pH adjustment; oxidation; disinfection; stabilisation) been installed to ensure easy maintenance and trouble-free operation? | 1 |  |
| Maximum possible score for ***Design Aspects*** | | **10** | |
| Total score attained for ***Design Aspects*** | | **10** | |
| Weight for ***Design Aspects*** | | **0.1** | |
| Total weighted score for ***Design Aspects*** | | **0.1** | |

| **Table 8.3**  **TECHNICAL COMPLIANCE SCORING:** | | | |
| --- | --- | --- | --- |
| **Criterion** | | **Yes =1** | **No = 0** |
| **1. TREATMENT PLANT MEASUREMENTS** | | | |
| **1B: Operation Monitoring Practices** | | | |
| Coagulation and Flocculation | Do the process controllers know how much they are dosing (even qualitatively)? | **1** |  |
|  | Can the process controllers adjust the dosage rates and monitor how much is dosed? | **1** |  |
|  | Do they have a programme for monitoring floc formation? |  | N/A |
| Sedimentation | Is the floc blanket observed and dislodging practices based thereon? |  | N/A |
|  | Is the overflow weir kept clean? |  | N/A |
|  | Is the flow of more than one sedimentation tank distributed evenly? |  | N/A |
| Filtration | Is backwashing done properly (at the right time and according to the correct procedure)? | **1** |  |
|  | Is the quality of the filtrate monitored on a regular basis? |  | N/A |
|  | Is excessive head loss development or turbidity breakthrough monitored? |  | N/A |
| Disinfection | Is the chlorine dosed according to previously determined chlorine demand and/or by maintaining acceptable chlorine residual in the final water? | **1** |  |
|  | Is the chlorine residual measured correctly and at the suggested frequency? | **1** |  |
| Stabilisation | Is the stability of the final water determined? | **1** |  |
|  | Is the process controller familiar with the reason for stabilisation and how it can be affected and controlled? | **1** |  |
| **Maximum possible score for *Operation Monitoring Practices*** | | **7** | |
| **Total score attained for *Operation Monitoring Practices*** | | **7** | |
| **Weight for *Operation Monitoring Practices*** | | **0.2** | |
| **Total weighted score for *Operation Monitoring Practices*** | | **0.2** | |

| **Table 8.4**  **TECHNICAL COMPLIANCE SCORING:** | | | |
| --- | --- | --- | --- |
| **Criterion** | | **Yes =1** | **No = 0** |
| **1. TREATMENT PLANT MEASUREMENTS** | | | |
| **1C: Compliance Monitoring Practices** | | | |
| Final water quality | Is the free chlorine residual sufficient? | **1** |  |
|  | Is the turbidity less than 1.0 NTU? | **1** |  |
|  | Is the pH between 6.0 and 9.0? | **1** |  |
|  | Is the colour less than 10 mg/L as Pt? |  | **N/A** |
|  | Is the final water free of any coliform indicator organisms? | **1** |  |
|  | Is the electrical conductivity less than 70 mS/m? |  | **N/A** |
|  | Is the iron level less than 0.2 mg/L as Fe? |  | **N/A** |
|  | Is the aluminium level less than 0.2 mg/L as Al? |  | **N/A** |
| Maximum possible score for ***Compliance Monitoring Practices*** | | **4** | |
| Total score attained for ***Compliance Monitoring Practices*** | | **4** | |
| Weight for ***Compliance Monitoring Practices*** | | **0.3** | |
| Total weighted score for ***Compliance Monitoring Practices*** | | **0.3** | |

| **Table 8.5**  **TECHNICAL COMPLIANCE SCORING:** | | | |
| --- | --- | --- | --- |
| **Criterion** | | **Yes =1** | **No = 0** |
| **1. TREATMENT PLANT MEASUREMENTS** | | | |
| **1D: Plant Monitoring Practices** | | | |
| Plant Infrastructure and Equipment | Are all the pumps in working order? | **1** |  |
|  | Can all the valves open and close properly? | **1** |  |
|  | Are all leaks repaired? | **1** |  |
|  | Are all blockages in pipes cleared? | **1** |  |
|  | Are all unit treatment processes and equipment accessible? | **1** |  |
| Maximum possible score for ***Plant Monitoring Practices*** | | **5** | |
| Total score attained for ***Plant Monitoring Practices*** | | **5** | |
| Weight for ***Plant Monitoring Practices*** | | **0.1** | |
| Total weighted score for ***Plant Monitoring Practices*** | | **0.1** | |

| **Table 8.6**  **TECHNICAL COMPLIANCE SCORING:** | | | |
| --- | --- | --- | --- |
| **Criterion** | | **Yes =1** | **No = 0** |
| **1. TREATMENT PLANT MEASUREMENTS** | | | |
| 1E: Maintenance Practices | | | |
| Overall Treatment Plant | Is back-up service available? | **1** |  |
|  | Is there opportunity for training of the plant personnel in using the sophisticated equipment? | **1** |  |
|  | Are spare-parts readily available? | **1** |  |
|  | Can spare parts be replaced quickly? | **1** |  |
|  | Are spare parts affordable by the municipality? | **1** |  |
|  | Is back-up service readily available? | **1** |  |
|  | Can service providers be on site at short notice? | **1** |  |
|  | Is this service reliable? | **1** |  |
|  | Can the community do certain parts of the maintenance themselves? |  | **N/A** |
|  | Are there adequate communication facilities between the plant and management, authorities, service providers, suppliers and consultants? | **1** |  |
|  | Can this be readily improved? | **1** |  |
|  | Can the plant personnel or persons in the community perform their own maintenance (or parts thereof)? |  | **N/A** |
|  | Can they be trained and are such training facilities available? | **1** |  |
|  | Is there good access to the plant by motor vehicles and delivery trucks? | **1** |  |
|  | Can the existing access routes be improved with existing funds? | **1** |  |
|  | Are funds available? | **1** |  |
|  | Is there possibility of alternative sources of funding for maintenance? | **1** |  |
| Maximum possible score for ***Maintenance Practices*** | | **15** | |
| Total score attained for ***Maintenance Practices*** | | **15** | |
| Weight for ***Maintenance Practices*** | | **0.2** | |
| Total weighted score for ***Maintenance Practices*** | | **0.2** | |

| **Table 8.7**  **TECHNICAL COMPLIANCE SCORING:** | | | |
| --- | --- | --- | --- |
| **Criterion** | | **Yes =1** | **No = 0** |
| **1. TREATMENT PLANT MEASUREMENTS** | | | |
| **1F: Risk Management Practices** | | | |
| Source to Tap | Is there a risk management system in place? | **1** |  |
|  | Are risk reduction options drawn up and implemented based on the risk assessment? | **1** |  |
|  | Are the risk assessments carried out on a regular basis? |  | **0** |
| Maximum possible score for ***Risk Management Practices*** | | **3** | |
| Total score attained for ***Risk Management Practices*** | | **2** | |
| Weight for ***Risk Management Practices*** | | **0.1** | |
| Total weighted score for ***Risk Management Practices*** | | **0.067** | |

| **TABLE 8.8**  **TECHNICAL COMPLIANCE SCORING:**  **Total Weighted Scoring Dzingahe package plant 96.7 %** | |
| --- | --- |
| **Compliance Criterion** | **Weighted Score** |
| **1. TREATMENT PLANT MEASUREMENTS** | |
| 1A: Design Aspects | **0.1** |
| 1B: Operation Monitoring Practices | **0.2** |
| 1C: Compliance (Final Water Quality) Monitoring Practices | **0.3** |
| 1D: Plant Monitoring Practices | **0.1** |
| 1E: Maintenance Practices | **0.2** |
| 1F: Risk Management Practices | **0.067** |
| TOTAL WEIGHTED SCORE | **1.0** |

**3.5 Technical compliance rating of small water treatment plants**

Based on the Technical Compliance Scoring system used above and the final total weighted score calculated for the specific water treatment plant, the plant can then be rated according to the following Technical Compliance Rating:

| **TABLE 8.9**  **TECHNICAL COMPLIANCE RATING** | |
| --- | --- |
| **Total Weighted Score** | **Rating Description** |
| **0 – 50** | Class 3 Compliance: Total non-compliance; serious and immediate intervention required (TAC) |
| **50 – 90** | Class 2 Compliance: Serious challenges requiring attention and improvement |
| **90 – 100** | Class 1 Compliance: Acceptable compliance |

The following chapter focuses on the corrective actions for non-technical compliance that can be recorded in a drinking water supply.

**NON-TECHNICAL COMPLIANCE**

**2009 AND 2011**

**MALAMULELE TREATMENT WATER PLANT**

19 August 2009

| **TABLE 1.1**  **NON-TECHNICAL (MANAGEMENT) COMPLIANCE SCORING** | |
| --- | --- |
| **Criterion** | **Weight** |
| **2. NON-TECHNICAL ASSESSMENT** | |
| **2A:** Management Issues | **0.1** |
| **2B:** Management Practices | **0.2** |
| **2C:** Human Resources | **0.2** |
| **2D:** Financial Systems | **0.1** |
| **2E:** Communication Systems | **0.2** |
| **2F:** Safety, Health and Environmental Quality | **0.15** |
| **2G:** Community Involvement and Awareness | **0.05** |
| **TOTAL** | **1.0** |

| **NON-TECHNICAL COMPLIANCE (MANAGEMENT) SCORING: Table 1.2** | | | | | |
| --- | --- | --- | --- | --- | --- |
| **Criterion** | | **Yes =1** | | **No = 0** | |
| **2. NON-TECHNICAL ASSESSMENT** | | | | | |
| **2A: Management Issues** | | | | | |
| **Assessment of strategic plans** | Does a strategic plan for the assessment of drinking water supply systems exist? | | **1** | |  |
|  | Are there preventive strategic plans for drinking water quality management? | | **1** | |  |
|  | Is there a strategic plan for recruitment of personnel per water treatment plant available? | | **1** | |  |
|  | Is there a strategic plan for operational monitoring and verification of drinking water system? | | **1** | |  |
|  | Is there a strategic plan for sharing and clarifying roles and responsibilities among all personnel involved in the production of safe drinking water quality? | | **1** | |  |
|  | Does a strategic plan for training and development of water plant personnel, and for community involvement and awareness exist? | | **1** | |  |
|  | Is there any funding strategic plan for implementation of drinking water quality management programmes? | | **1** | |  |
| **Assessment of tact plans** | Does a tactical plan for handling emergencies including communication of such emergencies to communities exist? | | **1** | |  |
|  | Is the overall periodic overseeing of water care function performed? | | **1** | |  |
|  | Is there maintenance and asset management plans? | | **1** | |  |
| **Assessment of operational plans** | Is the attendance of process controllers (and in particular shift workers) at the treatment plant monitored? Is there a clock–in-system for the attendance? | | **1** | |  |
|  | Is the superintendent available to operators on a day-to-day basis? | | **1** | |  |
|  | Is there a substitute system plan put in place when one of the operators is ill or away attending a course? | | **1** | |  |
|  | Are the supervisors and process controllers aware of their job requirements? Are job descriptions available? | | **1** | |  |
| **Maximum possible score for *Management Issues*** | | | **14** | | |
| **Total score attained for *Management Issues*** | | | **14** | | |
| **Weight for *Management Issues*** | | | **0.1** | | |
| **Total weighted score for *Management Issues*** | | | **0.1** | | |

| **NON-TECHNICAL COMPLIANCE (MANAGEMENT) SCORING: Table 1.3** | | | |
| --- | --- | --- | --- |
| **Criterion** | | **Yes =1** | **No = 0** |
| **2. NON-TECHNICAL ASSESSMENT** | | | |
| **2B: Management Practices** | | | |
|  | Do the WSPs use existing local (Emanti/DWAF) or international management practices? Are they implementing them in their water treatment plants? | **1** |  |
|  | Are the WPSs able to understand the existing local or international practices and implementing them correctly? | **1** |  |
|  | When local or international practices are not available, are the WSPs able to draw their own criteria? | **1** |  |
| **Maximum possible score for *Management Practices*** | | **3** | |
| **Total score attained for *Management Practices*** | | **3** | |
| **Weight for *Management Practices*** | | **0.2** | |
| **Total weighted score for *Management Practices*** | | **0.2** | |

| **NON-TECHNICAL COMPLIANCE (MANAGEMENT) SCORING: Table 1.4** | | | | | |
| --- | --- | --- | --- | --- | --- |
| **Criterion** | | **Yes =1** | | **No = 0** | |
| **2. NON-TECHNICAL ASSESSMENT** | | | | | |
| **2C: Human Resources** | | | | | |
| **Personnel at the plant** | Is the number of personnel related to the size of the plant? | |  | | **0** |
|  | Are the needs of process controllers/supervisors and shift workers per plants adequate? | |  | | **0** |
|  | Are there specific guidelines/rules for shift workers and their deployment? | | **1** | |  |
|  | How many operators are there in total? How many shift workers? | | **1** | |  |
| **Qualifications of Personnel** | How many operators with qualifications in the DWAF categories? | | **1** | |  |
|  | Are personnel being correctly applied according to their qualifications? | | **1** | |  |
|  | Are there guidelines or formal rules for upgrading of qualifications? | | **1** | |  |
| **Training Needs** | Are there in service training programmes and policies? What is the in-service training policy of the WSA? | | **1** | |  |
|  | Do process controllers / supervisors / managers know what training opportunities and courses are available? | | **1** | |  |
|  | Are they sent on courses regularly? | |  | | **0** |
|  | Are there specific criteria or formal rules to be qualified for training? | | **1** | |  |
|  | Are there specific promotion requirement protocols in place? | | **1** | |  |
|  | Are there career advancement opportunities? | | **1** | |  |
| **Plant class** | Is the plant classified by DWAF? | |  | | **0** |
|  | Has it been done according to the new classification system? | |  | | **0** |
| **Working Conditions** | Are the responsibilities of process controllers, supervisors and plant managers in line with their salary? | |  | | **0** |
|  | What system is used for overtime management and payment? | | **1** | |  |
|  | What is the WSA policy on conflict management (internal and external)? | | **1** | |  |
|  | **Is any medical assistance available for on the job injuries?** | | **1** | |  |
|  | Can all workers participate in medical schemes? | | **1** | |  |
|  | Are the retirement/pension-aid benefits, the housing and travel allowance benefits available? | | **1** | |  |
|  | Are the leave benefits (vacation /study/ sick), and the bonus scheme available? | | **1** | |  |
|  | Are there any other incentives? | | **1** | |  |
|  | Are plant personnel satisfied with their working environment? | | **1** | |  |
|  | Are the relationships between supervisors and their subordinates good or poor? | | **1** | |  |
|  | Is there any performance indicators? | | **1** | |  |
| **Maximum possible score for *Human Resources*** | | | **27** | | |
| **Total score attained for *Human Resources*** | | | **22** | | |
| **Weight for *Human Resources*** | | | **0.2** | | |
| **Total weighted score for *Human Resources*** | | | **0.163** | | |

| **NON-TECHNICAL COMPLIANCE (MANAGEMENT) SCORING: Table 1.5** | | | |
| --- | --- | --- | --- |
| Criterion | | Yes =1 | No = 0 |
| 2. NON-TECHNICAL ASSESSMENT | | | |
| 2D: Financial Systems | | | |
| **Financial Systems Sharing in Place** | Which financial systems are in place? | **1** |  |
|  | Are there sufficient funds for operation, maintenance and overtime? |  | **0** |
|  | Do the size of the plants (small versus large) influence the allocation of funds? | **1** |  |
|  | Does the WSA’s budgeting system work in relation to emergency funds, scheduled upgrading/extensions, unscheduled rehabilitation/upgrading? | **1** |  |
| **Information Sharing in Place** | Are there communication channels and transparency between the financial section and the water care section? | **1** |  |
|  | Are regular meetings held? Who attends / chairs these meetings? | **1** |  |
|  | Are decisions taken at consensus / majority? | **1** |  |
|  | Are reports freely available? | **1** |  |
| **Procurement System** | Is the procurement system transparent? | **1** |  |
|  | Is the procurement system open to all? | **1** |  |
|  | Is the water sector involved in the adjudication of tenders? | **1** |  |
| **Maximum possible score for *Financial Systems*** | | **11** | |
| **Total score attained for *Financial Systems*** | | **10** | |
| **Weight for *Financial Systems*** | | **0.1** | |
| **Total weighted score for *Financial Systems*** | | **0.0091** | |

| **NON-TECHNICAL COMPLIANCE (MANAGEMENT) SCORING: Table 1.6** | | | |
| --- | --- | --- | --- |
| Criterion | | Yes =1 | No = 0 |
| 2. NON-TECHNICAL ASSESSMENT | | | |
| 2E: Communication Systems | | | |
| **Internal** | Are there sufficient internal communication channels in place, including the needs and shortcomings? | **1** |  |
|  | Are these communication channels effective? | **1** |  |
|  | Is the communication between supervisors and process controllers good or poor? | **1** |  |
|  | Is there any interaction between the maintenance team and the rest of personnel in the water treatment plant? | **1** |  |
|  | Is the communication between management and lower level process controllers good or poor? | **1** |  |
| **External** | Are there sufficient communication channels with other Municipalities and/or Departments? | **1** |  |
|  | Are there sufficient communication channels with consumers? | **1** |  |
|  | Are these external communication channels effective, good or poor? | **1** |  |
| **Maximum possible score for *Communication Systems*** | | **8** | |
| **Total score attained for *Communication Systems*** | | **8** | |
| **Weight for *Communication Systems*** | | **0.2** | |
| **Total weighted score for *Communication Systems*** | | **0.2** | |

| **NON-TECHNICAL COMPLIANCE (MANAGEMENT) SCORING: Table 1.7** | | | |
| --- | --- | --- | --- |
| **Criterion** | | **Yes =1** | **No = 0** |
| **2. NON-TECHNICAL ASSESSMENT** | | | |
| **2F: Audit of safety, health and environmental quality** | | | |
| **Safety Matters** | Are there water and safety management procedures for normal operation, incidents and emergency situations? | **1** |  |
|  | Is there any safety plan at water treatment plants including the safety of process controllers on duties? | **1** |  |
|  | Are there emergency plans in place for chlorine leaks? | **1** |  |
|  | Are there hazardous chemicals and ablution facilities, appropriate fire extinguishers location? | **1** |  |
|  | Are safety meetings held regularly and who attends such meetings? | **1** |  |
| **Health Aspects** | Are there representatives for health and safety of consumers? If yes, are they effective? | **1** |  |
|  | Are there control measures that identify risks and ensure that health based- targets are met in terms of providing safe drinking water to all consumers? | **1** |  |
|  | Are there emergency measures in place for water quality health impact? | **1** |  |
| **Environmental Quality** | Are process controllers satisfied with their working environment? | **1** |  |
|  | Is the working environment appropriate for improving or accelerating the service delivery? | **1** |  |
|  | Is there any strategic plan to improve the quality of the working environment? | **1** |  |
| **Maximum possible score for *SHEQ*** | | **11** | |
| **Total score attained for *SHEQ*** | | **11** | |
| **Weight for *SHEQ*** | | **0.15** | |
| **Total weighted score for *SHEQ*** | | **0.15** | |

| **NON-TECHNICAL COMPLIANCE (MANAGEMENT) SCORING: Table 1.8** | | | |
| --- | --- | --- | --- |
| **Criterion** | | **Yes =1** | **No = 0** |
| **2. NON-TECHNICAL ASSESSMENT** | | | |
| **2G: Community involvement and awareness** | | | |
| **Community Involvement** | Are there protocols involved communities in decision-making? |  | **0** |
|  | Is there an effective reporting system for communities to assist in more rapid response to any water quality incident? |  | **0** |
|  | Is there a Consumer Service to which non-compliance can be reported? |  | **0** |
| **Community Awareness** | Is there a mechanism system to receive and actively address community complaints in a timely fashion? | **1** |  |
|  | Are there procedures for promptly advising of any significant incidents within the drinking water supply including notification of the public health authority? | **1** |  |
|  | Is there any water quality information system available to consumers through annual reports and in the internet? | **1** |  |
| **Maximum possible score for *Community Involvement*** | | **6** | |
| **Total score attained for *Community Involvement*** | | **3** | |
| **Weight for *Community Involvement*** | | **0.05** | |
| **Total weighted score for *Community Involvement*** | | **0.025** | |

| **TABLE 1.9**  **NON-TECHNICAL (MANAGEMENT) COMPLIANCE SCORING:**  **Total Weighted Scoring for Malamulele treatment water plant 92.9 %** | |
| --- | --- |
| **Compliance Criterion** | **Weighted Score** |
| **2. NON-TECHNICAL ASSESSMENT** | |
| **2A: Management Issues** | **0.1** |
| **2B: Management Practices** | **0.2** |
| **2C: Human Resources** | **0.163** |
| **2D: Financial Systems** | **0.091** |
| **2E: Communication Systems** | **0.2** |
| **2F: Safety, Health and Environmental Quality** | **0.15** |
| **2G: Community Involvement and Awareness** | **0.025** |
| **TOTAL WEIGHTED SCORE** | **0.929** |

| **TABLE 1.10**  **NON-TECHNICAL (MANAGEMENT) COMPLIANCE RATING:** | |
| --- | --- |
| **Total Weighted Score** | **Rating Description** |
| **0 – 50** | ***Class 3 Compliance*:**  Total non-compliance; serious and immediate intervention required (TAC) |
| **50 – 90** | ***Class 2 Compliance:***  Serious challenges requiring attention and improvement |
| **90 – 100** | ***Class 1 Compliance:***  Acceptable compliance |

**Vondo treatment Plant**

18 August 2009

| **TABLE 2.1**  **NON-TECHNICAL (MANAGEMENT) COMPLIANCE SCORING** | |
| --- | --- |
| **Criterion** | **Weight** |
| **2. NON-TECHNICAL ASSESSMENT** | |
| **2A: Management Issues** | **0.1** |
| **2B: Management Practices** | **0.2** |
| **2C: Human Resources** | **0.2** |
| **2D: Financial Systems** | **0.1** |
| **2E: Communication Systems** | **0.2** |
| **2F: Safety, Health and Environmental Quality** | **0.15** |
| **2G: Community Involvement and Awareness** | **0.05** |
| **TOTAL** | **1.0** |

| **NON-TECHNICAL COMPLIANCE (MANAGEMENT) SCORING: Table 2.2** | | | | | |
| --- | --- | --- | --- | --- | --- |
| **Criterion** | | **Yes =1** | | **No = 0** | |
| **2. NON-TECHNICAL ASSESSMENT** | | | | | |
| **2A: Management Issues** | | | | | |
| **Assessment of strategic plans** | Does a strategic plan for the assessment of drinking water supply systems exist? | | **1** | |  |
|  | Are there preventive strategic plans for drinking water quality management? | | **1** | |  |
|  | Is there a strategic plan for recruitment of personnel per water treatment plant available? | | **1** | |  |
|  | Is there a strategic plan for operational monitoring and verification of drinking water system? | | **1** | |  |
|  | Is there a strategic plan for sharing and clarifying roles and responsibilities among all personnel involved in the production of safe drinking water quality? | | **1** | |  |
|  | Does a strategic plan for training and development of water plant personnel, and for community involvement and awareness exist? | | **1** | |  |
|  | Is there any funding strategic plan for implementation of drinking water quality management programmes? | | **1** | |  |
| **Assm of tact plans** | Does a tactical plan for handling emergencies including communication of such emergencies to communities exist? | | **1** | |  |
|  | Is the overall periodic overseeing of water care function performed? | | **1** | |  |
|  | Is there maintenance and asset management plans? | | **1** | |  |
| **Assessment of operational plans** | Is the attendance of process controllers (and in particular shift workers) at the treatment plant monitored? Is there a clock–in-system for the attendance? | | **1** | |  |
|  | Is the superintendent available to operators on a day-to-day basis? | | **1** | |  |
|  | Is there a substitute system plan put in place when one of the operators is ill or away attending a course? | | **1** | |  |
|  | Are the supervisors and process controllers aware of their job requirements? Are job descriptions available? | | **1** | |  |
| **Maximum possible score for *Management Issues*** | | | **14** | | |
| **Total score attained for *Management Issues*** | | | **14** | | |
| **Weight for *Management Issues*** | | | **0.1** | | |
| **Total weighted score for *Management Issues*** | | | **0.1** | | |

| **NON-TECHNICAL COMPLIANCE (MANAGEMENT) SCORING: Table 2.3** | | | |
| --- | --- | --- | --- |
| **Criterion** | | **Yes =1** | **No = 0** |
| **2. NON-TECHNICAL ASSESSMENT** | | | |
| **2B: Management Practices** | | | |
|  | Do the WSPs use existing local (Emanti/DWAF) or international management practices? Are they implementing them in their water treatment plants? | **1** |  |
|  | Are the WPSs able to understand the existing local or international practices and implementing them correctly? | **1** |  |
|  | When local or international practices are not available, are the WSPs able to draw their own criteria? | **1** |  |
| **Maximum possible score for *Management Practices*** | | **3** | |
| **Total score attained for *Management Practices*** | | **3** | |
| **Weight for *Management Practices*** | | **0.2** | |
| **Total weighted score for *Management Practices*** | | **0.2** | |

| **NON-TECHNICAL COMPLIANCE (MANAGEMENT) SCORING: Table 2.4** | | | | | |
| --- | --- | --- | --- | --- | --- |
| **Criterion** | | **Yes =1** | | **No = 0** | |
| **2. NON-TECHNICAL ASSESSMENT** | | | | | |
| **2C: Human Resources** | | | | | |
| **Personnel at the plant** | Is the number of personnel related to the size of the plant? | |  | | **0** |
|  | Are the needs of process controllers/supervisors and shift workers per plants adequate? | |  | | **0** |
|  | Are there specific guidelines/rules for shift workers and their deployment? | | **1** | |  |
|  | How many operators are there in total? How many shift workers? | | **1** | |  |
| **Qualifications of Personnel** | How many operators with qualifications in the DWAF categories? | | **1** | |  |
|  | Are personnel being correctly applied according to their qualifications? | | **1** | |  |
|  | Are there guidelines or formal rules for upgrading of qualifications? | | **1** | |  |
| **Training Needs** | Are there in service training programmes and policies? What is the in-service training policy of the WSA? | | **1** | |  |
|  | Do process controllers / supervisors / managers know what training opportunities and courses are available? | | **1** | |  |
|  | Are they sent on courses regularly? | | **1** | |  |
|  | Are there specific criteria or formal rules to be qualified for training? | | **1** | |  |
|  | Are there specific promotion requirement protocols in place? | | **1** | |  |
|  | Are there career advancement opportunities? | | **1** | |  |
| **Plant class** | Is the plant classified by DWAF? | |  | | **0** |
|  | Has it been done according to the new classification system? | |  | | **0** |
| **Working Conditions** | Are the responsibilities of process controllers, supervisors and plant managers in line with their salary? | |  | | **0** |
|  | What system is used for overtime management and payment? | | **1** | |  |
|  | What is the WSA policy on conflict management (internal and external)? | | **1** | |  |
|  | Is any medical assistance available for on the job injuries? | | **1** | |  |
|  | Can all workers participate in medical schemes? | | **1** | |  |
|  | Are the retirement/pension-aid benefits, the housing and travel allowance benefits available? | | **1** | |  |
|  | Are the leave benefits (vacation /study/ sick), and the bonus scheme available? | | **1** | |  |
|  | Are there any other incentives? | | **1** | |  |
|  | Are plant personnel satisfied with their working environment? | | **1** | |  |
|  | Are the relationships between supervisors and their subordinates good or poor? | | **1** | |  |
|  | Is there any performance indicators? | | **1** | |  |
| **Maximum possible score for *Human Resources*** | | | **27** | | |
| **Total score attained for *Human Resources*** | | | **22** | | |
| **Weight for *Human Resources*** | | | **0.2** | | |
| **Total weighted score for *Human Resources*** | | | **0.163** | | |

| **NON-TECHNICAL COMPLIANCE (MANAGEMENT) SCORING: Table 2.5** | | | |
| --- | --- | --- | --- |
| **Criterion** | | **Yes =1** | **No = 0** |
| **2. NON-TECHNICAL ASSESSMENT** | | | |
| **2D: Financial Systems** | | | |
| **Financial Systems Sharing in Place** | Which financial systems are in place? | **1** |  |
|  | Are there sufficient funds for operation, maintenance and overtime? | **1** |  |
|  | Do the size of the plants (small versus large) influence the allocation of funds? | **1** |  |
|  | Does the WSA’s budgeting system work in relation to emergency funds, scheduled upgrading/extensions, unscheduled rehabilitation/upgrading? | **1** |  |
| **Information Sharing in Place** | Are there communication channels and transparency between the financial section and the water care section? | **1** |  |
|  | Are regular meetings held? Who attends / chairs these meetings? | **1** |  |
|  | Are decisions taken at consensus / majority? | **1** |  |
|  | Are reports freely available? | **1** |  |
| **Procurement System** | Is the procurement system transparent? | **1** |  |
|  | Is the procurement system open to all? | **1** |  |
|  | Is the water sector involved in the adjudication of tenders? | **1** |  |
| **Maximum possible score for *Financial Systems*** | | **11** | |
| **Total score attained for *Financial Systems*** | | **11** | |
| **Weight for *Financial Systems*** | | **0.1** | |
| **Total weighted score for *Financial Systems*** | | **0.1** | |

| **NON-TECHNICAL COMPLIANCE (MANAGEMENT) SCORING: Table 2.6** | | | |
| --- | --- | --- | --- |
| **Criterion** | | **Yes =1** | **No = 0** |
| **2. NON-TECHNICAL ASSESSMENT** | | | |
| **2E: Communication Systems** | | | |
| **Internal** | Are there sufficient internal communication channels in place, including the needs and shortcomings? | **1** |  |
|  | Are these communication channels effective? | **1** |  |
|  | Is the communication between supervisors and process controllers good or poor? | **1** |  |
|  | Is there any interaction between the maintenance team and the rest of personnel in the water treatment plant? | **1** |  |
|  | Is the communication between management and lower level process controllers good or poor? | **1** |  |
| **External** | Are there sufficient communication channels with other Municipalities and/or Departments? | **1** |  |
|  | Are there sufficient communication channels with consumers? | **1** |  |
|  | Are these external communication channels effective, good or poor? | **1** |  |
| **Maximum possible score for *Communication Systems*** | | **8** | |
| **Total score attained for *Communication Systems*** | | **8** | |
| **Weight for *Communication Systems*** | | **0.2** | |
| **Total weighted score for *Communication Systems*** | | **0.2** | |

| **NON-TECHNICAL COMPLIANCE (MANAGEMENT) SCORING: Table 2.7** | | | |
| --- | --- | --- | --- |
| **Criterion** | | **Yes =1** | **No = 0** |
| **2. NON-TECHNICAL ASSESSMENT** | | | |
| **2F: Audit of safety, health and environmental quality** | | | |
| **Safety Matters** | Are there water and safety management procedures for normal operation, incidents and emergency situations? | **1** |  |
|  | Is there any safety plan at water treatment plants including the safety of process controllers on duties? | **1** |  |
|  | Are there emergency plans in place for chlorine leaks? | **1** |  |
|  | Are there hazardous chemicals and ablution facilities, appropriate fire extinguishers location? | **1** |  |
|  | Are safety meetings held regularly and who attends such meetings? | **1** |  |
| **Health Aspects** | Are there representatives for health and safety of consumers? If yes, are they effective? | **1** |  |
|  | Are there control measures that identify risks and ensure that health based- targets are met in terms of providing safe drinking water to all consumers? | **1** |  |
|  | Are there emergency measures in place for water quality health impact? | **1** |  |
| **Environmental Quality** | Are process controllers satisfied with their working environment? | **1** |  |
|  | Is the working environment appropriate for improving or accelerating the service delivery? | **1** |  |
|  | Is there any strategic plan to improve the quality of the working environment? |  | **0** |
| **Maximum possible score for *SHEQ*** | | **11** | |
| **Total score attained for *SHEQ*** | | **10** | |
| **Weight for *SHEQ*** | | **0.15** | |
| **Total weighted score for *SHEQ*** | | **0.136** | |

| **NON-TECHNICAL COMPLIANCE (MANAGEMENT) SCORING: Table 2.8** | | | |
| --- | --- | --- | --- |
| **Criterion** | | **Yes =1** | **No = 0** |
| **2. NON-TECHNICAL ASSESSMENT** | | | |
| **2G: Community involvement and awareness** | | | |
| **Community Involvement** | Are there protocols involved communities in decision-making? | **1** |  |
|  | Is there an effective reporting system for communities to assist in more rapid response to any water quality incident? | **1** |  |
|  | Is there a Consumer Service to which non-compliance can be reported? | **1** |  |
| **Community Awareness** | Is there a mechanism system to receive and actively address community complaints in a timely fashion? | **1** |  |
|  | Are there procedures for promptly advising of any significant incidents within the drinking water supply including notification of the public health authority? | **1** |  |
|  | Is there any water quality information system available to consumers through annual reports and in the internet? |  | **0** |
| **Maximum possible score for *Community Involvement*** | | **6** | |
| **Total score attained for *Community Involvement*** | | **5** | |
| **Weight for *Community Involvement*** | | **0.05** | |
| **Total weighted score for *Community Involvement*** | | **0.042** | |

| **TABLE 2.9**  **NON-TECHNICAL (MANAGEMENT) COMPLIANCE SCORING:**  **Total Weighted Scoring for Vondo treatment plant 94.1 %** | |
| --- | --- |
| **Compliance Criterion** | **Weighted Score** |
| **2. NON-TECHNICAL ASSESSMENT** | |
| **2A: Management Issues** | **0.1** |
| **2B: Management Practices** | **0.2** |
| **2C: Human Resources** | **0.163** |
| **2D: Financial Systems** | **0.1** |
| **2E: Communication Systems** | **0.2** |
| **2F: Safety, Health and Environmental Quality** | **0.136** |
| **2G: Community Involvement and Awareness** | **0.042** |
| **TOTAL WEIGHTED SCORE** | **0.941** |

| **TABLE 2.10**  **NON-TECHNICAL (MANAGEMENT) COMPLIANCE RATING:** | |
| --- | --- |
| **Total Weighted Score** | **Rating Description** |
| **0 – 50** | ***Class 3 Compliance*:**  Total non-compliance; serious and immediate intervention required (TAC) |
| **50 – 90** | ***Class 2 Compliance:***  Serious challenges requiring attention and improvement |
| **90 – 100** | ***Class 1 Compliance:***  Acceptable compliance |

**mutshedzi Plant**

**19 August 2009**

| **TABLE 3.1**  **NON-TECHNICAL (MANAGEMENT) COMPLIANCE SCORING** | |
| --- | --- |
| **Criterion** | **Weight** |
| **2. NON-TECHNICAL ASSESSMENT** | |
| **2A: Management Issues** | **0.1** |
| **2B: Management Practices** | **0.2** |
| **2C: Human Resources** | **0.2** |
| **2D: Financial Systems** | **0.1** |
| **2E: Communication Systems** | **0.2** |
| **2F: Safety, Health and Environmental Quality** | **0.15** |
| **2G: Community Involvement and Awareness** | **0.05** |
| **TOTAL** | **1.0** |

| **NON-TECHNICAL COMPLIANCE (MANAGEMENT) SCORING: Table 3.2** | | | | | |
| --- | --- | --- | --- | --- | --- |
| **Criterion** | | **Yes =1** | | **No = 0** | |
| **2. NON-TECHNICAL ASSESSMENT** | | | | | |
| **2A: Management Issues** | | | | | |
| **Assessment of strategic plans** | Does a strategic plan for the assessment of drinking water supply systems exist? | | **1** | |  |
|  | Are there preventive strategic plans for drinking water quality management? | | **1** | |  |
|  | Is there a strategic plan for recruitment of personnel per water treatment plant available? | | **1** | |  |
|  | Is there a strategic plan for operational monitoring and verification of drinking water system? | | **1** | |  |
|  | Is there a strategic plan for sharing and clarifying roles and responsibilities among all personnel involved in the production of safe drinking water quality? | | **1** | |  |
|  | Does a strategic plan for training and development of water plant personnel, and for community involvement and awareness exist? | | **1** | |  |
|  | Is there any funding strategic plan for implementation of drinking water quality management programmes? | | **1** | |  |
| **Assessment of tact plans** | Does a tactical plan for handling emergencies including communication of such emergencies to communities exist? | | **1** | |  |
|  | Is the overall periodic overseeing of water care function performed? | | **1** | |  |
|  | Is there maintenance and asset management plans? | | **1** | |  |
| **Assessment of operational plans** | Is the attendance of process controllers (and in particular shift workers) at the treatment plant monitored? Is there a clock–in-system for the attendance? | | **1** | |  |
|  | Is the superintendent available to operators on a day-to-day basis? | | **1** | |  |
|  | Is there a substitute system plan put in place when one of the operators is ill or away attending a course? | | **1** | |  |
|  | Are the supervisors and process controllers aware of their job requirements? Are job descriptions available? | | **1** | |  |
| **Maximum possible score for *Management Issues*** | | | **14** | | |
| **Total score attained for *Management Issues*** | | | **14** | | |
| **Weight for *Management Issues*** | | | **0.1** | | |
| **Total weighted score for *Management Issues*** | | | **0.1** | | |

| **NON-TECHNICAL COMPLIANCE (MANAGEMENT) SCORING: Table 3.3** | | | |
| --- | --- | --- | --- |
| **Criterion** | | **Yes =1** | **No = 0** |
| **2. NON-TECHNICAL ASSESSMENT** | | | |
| **2B: Management Practices** | | | |
|  | Do the WSPs use existing local (Emanti/DWAF) or international management practices? Are they implementing them in their water treatment plants? | **1** |  |
|  | Are the WPSs able to understand the existing local or international practices and implementing them correctly? | **1** |  |
|  | When local or international practices are not available, are the WSPs able to draw their own criteria? | **1** |  |
| **Maximum possible score for *Management Practices*** | | **3** | |
| **Total score attained for *Management Practices*** | | **3** | |
| **Weight for *Management Practices*** | | **0.2** | |
| **Total weighted score for *Management Practices*** | | **0.2** | |

| **NON-TECHNICAL COMPLIANCE (MANAGEMENT) SCORING: Table 3.4** | | | | | |
| --- | --- | --- | --- | --- | --- |
| **Criterion** | | **Yes =1** | | **No = 0** | |
| **2. NON-TECHNICAL ASSESSMENT** | | | | | |
| **2C: Human Resources** | | | | | |
| **Personnel at the plant** | Is the number of personnel related to the size of the plant? | |  | | **0** |
|  | Are the needs of process controllers/supervisors and shift workers per plants adequate? | |  | | **0** |
|  | Are there specific guidelines/rules for shift workers and their deployment? | | **1** | |  |
|  | How many operators are there in total? How many shift workers? | | **1** | |  |
| **Qualifications of Personnel** | How many operators with qualifications in the DWAF categories? | | **1** | |  |
|  | Are personnel being correctly applied according to their qualifications? | | **1** | |  |
|  | Are there guidelines or formal rules for upgrading of qualifications? | | **1** | |  |
| **Training Needs** | Are there in service training programmes and policies? What is the in-service training policy of the WSA? | | **1** | |  |
|  | Do process controllers / supervisors / managers know what training opportunities and courses are available? | | **1** | |  |
|  | Are they sent on courses regularly? | |  | | **0** |
|  | Are there specific criteria or formal rules to be qualified for training? | | **1** | |  |
|  | Are there specific promotion requirement protocols in place? | | **1** | |  |
|  | Are there career advancement opportunities? | | **1** | |  |
| **Plant class** | Is the plant classified by DWAF? | |  | | **0** |
|  | Has it been done according to the new classification system? | |  | | **0** |
| **Working Conditions** | Are the responsibilities of process controllers, supervisors and plant managers in line with their salary? | |  | | **0** |
|  | What system is used for overtime management and payment? | | **1** | |  |
|  | What is the WSA policy on conflict management (internal and external)? | | **1** | |  |
|  | Is any medical assistance available for on the job injuries? | | **1** | |  |
|  | Can all workers participate in medical schemes? | | **1** | |  |
|  | Are the retirement/pension-aid benefits, the housing and travel allowance benefits available? | | **1** | |  |
|  | Are the leave benefits (vacation /study/ sick), and the bonus scheme available? | | **1** | |  |
|  | Are there any other incentives? | | **1** | |  |
|  | Are plant personnel satisfied with their working environment? | | **1** | |  |
|  | Are the relationships between supervisors and their subordinates good or poor? | | **1** | |  |
|  | Is there any performance indicators? | | **1** | |  |
| **Maximum possible score for *Human Resources*** | | | **27** | | |
| **Total score attained for *Human Resources*** | | | **21** | | |
| **Weight for *Human Resources*** | | | **0.2** | | |
| **Total weighted score for *Human Resources*** | | | **0.156** | | |

| **NON-TECHNICAL COMPLIANCE (MANAGEMENT) SCORING: Table 3.5** | | | |
| --- | --- | --- | --- |
| **Criterion** | | **Yes =1** | **No = 0** |
| **2. NON-TECHNICAL ASSESSMENT** | | | |
| **2D: Financial Systems** | | | |
| **Financial Systems Sharing in Place** | Which financial systems are in place? | 1 |  |
|  | Are there sufficient funds for operation, maintenance and overtime? | 1 |  |
|  | Do the size of the plants (small versus large) influence the allocation of funds? | 1 |  |
|  | Does the WSA’s budgeting system work in relation to emergency funds, scheduled upgrading/extensions, unscheduled rehabilitation/upgrading? | 1 |  |
| **Information Sharing in Place** | Are there communication channels and transparency between the financial section and the water care section? | **1** |  |
|  | Are regular meetings held? Who attends / chairs these meetings? | **1** |  |
|  | Are decisions taken at consensus / majority? | **1** |  |
|  | Are reports freely available? | **1** |  |
| **Procurement System** | Is the procurement system transparent? | **1** |  |
|  | Is the procurement system open to all? | **1** |  |
|  | Is the water sector involved in the adjudication of tenders? | **1** |  |
| **Maximum possible score for *Financial Systems*** | | **11** | |
| **Total score attained for *Financial Systems*** | | **11** | |
| **Weight for *Financial Systems*** | | **0.1** | |
| **Total weighted score for *Financial Systems*** | | **0.1** | |

| **NON-TECHNICAL COMPLIANCE (MANAGEMENT) SCORING: Table 3.6** | | | |
| --- | --- | --- | --- |
| **Criterion** | | **Yes =1** | **No = 0** |
| **2. NON-TECHNICAL ASSESSMENT** | | | |
| **2E: Communication Systems** | | | |
| **Internal** | Are there sufficient internal communication channels in place, including the needs and shortcomings? | **1** |  |
|  | Are these communication channels effective? | **1** |  |
|  | Is the communication between supervisors and process controllers good or poor? | **1** |  |
|  | Is there any interaction between the maintenance team and the rest of personnel in the water treatment plant? | **1** |  |
|  | Is the communication between management and lower level process controllers good or poor? | **1** |  |
| **External** | Are there sufficient communication channels with other Municipalities and/or Departments? | **1** |  |
|  | Are there sufficient communication channels with consumers? | **1** |  |
|  | Are these external communication channels effective, good or poor? | **1** |  |
| **Maximum possible score for *Communication Systems*** | | **8** | |
| **Total score attained for *Communication Systems*** | | **8** | |
| **Weight for *Communication Systems*** | | **0.2** | |
| **Total weighted score for *Communication Systems*** | | **0.2** | |

| **NON-TECHNICAL COMPLIANCE (MANAGEMENT) SCORING: Table 3.7** | | | |
| --- | --- | --- | --- |
| **Criterion** | | **Yes =1** | **No = 0** |
| **2. NON-TECHNICAL ASSESSMENT** | | | |
| **2F: Audit of safety, health and environmental quality** | | | |
| **Safety Matters** | Are there water and safety management procedures for normal operation, incidents and emergency situations? | **1** |  |
|  | Is there any safety plan at water treatment plants including the safety of process controllers on duties? | **1** |  |
|  | Are there emergency plans in place for chlorine leaks? | **1** |  |
|  | Are there hazardous chemicals and ablution facilities, appropriate fire extinguishers location? | **1** |  |
|  | Are safety meetings held regularly and who attends such meetings? | **1** |  |
| **Health Aspects** | Are there representatives for health and safety of consumers? If yes, are they effective? | **1** |  |
|  | Are there control measures that identify risks and ensure that health based- targets are met in terms of providing safe drinking water to all consumers? | **1** |  |
|  | Are there emergency measures in place for water quality health impact? | **1** |  |
| **Environmental Quality** | Are process controllers satisfied with their working environment? | **1** |  |
|  | Is the working environment appropriate for improving or accelerating the service delivery? | **1** |  |
|  | Is there any strategic plan to improve the quality of the working environment? | **1** |  |
| **Maximum possible score for *SHEQ*** | | **11** | |
| **Total score attained for *SHEQ*** | | **11** | |
| **Weight for *SHEQ*** | | **0.15** | |
| **Total weighted score for *SHEQ*** | | **0.15** | |

| **NON-TECHNICAL COMPLIANCE (MANAGEMENT) SCORING: Table 3.8** | | | |
| --- | --- | --- | --- |
| **Criterion** | | **Yes =1** | **No = 0** |
| **2. NON-TECHNICAL ASSESSMENT** | | | |
| **2G: Community involvement and awareness** | | | |
| **Community Involvement** | Are there protocols involved communities in decision-making? |  | **0** |
|  | Is there an effective reporting system for communities to assist in more rapid response to any water quality incident? | **1** |  |
|  | Is there a Consumer Service to which non-compliance can be reported? | **1** |  |
| **Community Awareness** | Is there a mechanism system to receive and actively address community complaints in a timely fashion? | **1** |  |
|  | Are there procedures for promptly advising of any significant incidents within the drinking water supply including notification of the public health authority? | **1** |  |
|  | Is there any water quality information system available to consumers through annual reports and in the internet? | **1** |  |
| **Maximum possible score for *Community Involvement*** | | **6** | |
| **Total score attained for *Community Involvement*** | | **5** | |
| **Weight for *Community Involvement*** | | **0.05** | |
| **Total weighted score for *Community Involvement*** | | **0.042** | |

| **TABLE 3.9**  **NON-TECHNICAL (MANAGEMENT) COMPLIANCE SCORING:**  **Total Weighted Scoring for Mutshedzi package plant 94.7 %** | |
| --- | --- |
| **Compliance Criterion** | **Weighted Score** |
| **2. NON-TECHNICAL ASSESSMENT** | |
| **2A: Management Issues** | **0.1** |
| **2B: Management Practices** | **0.2** |
| **2C: Human Resources** | **0.156** |
| **2D: Financial Systems** | **0.1** |
| **2E: Communication Systems** | **0.2** |
| **2F: Safety, Health and Environmental Quality** | **0.15** |
| **2G: Community Involvement and Awareness** | **0.042** |
| **TOTAL WEIGHTED SCORE** | **0.947** |

| **TABLE 3.10**  **NON-TECHNICAL (MANAGEMENT) COMPLIANCE RATING:** | |
| --- | --- |
| **Total Weighted Score** | **Rating Description** |
| **0 – 50** | ***Class 3 Compliance*:**  Total non-compliance; serious and immediate intervention required (TAC) |
| **50 – 90** | ***Class 2 Compliance:***  Serious challenges requiring attention and improvement |
| **90 – 100** | ***Class 1 Compliance:***  Acceptable compliance |

**MUTALE REGIONAL WATER TREATMENT Plant**

**19 August 2009**

| **TABLE 4.1**  **NON-TECHNICAL (MANAGEMENT) COMPLIANCE SCORING** | |
| --- | --- |
| **Criterion** | **Weight** |
| **2. NON-TECHNICAL ASSESSMENT** | |
| **2A: Management Issues** | **0.1** |
| **2B: Management Practices** | **0.2** |
| **2C: Human Resources** | **0.2** |
| **2D: Financial Systems** | **0.1** |
| **2E: Communication Systems** | **0.2** |
| **2F: Safety, Health and Environmental Quality** | **0.15** |
| **2G: Community Involvement and Awareness** | **0.05** |
| **TOTAL** | **1.0** |

| **NON-TECHNICAL COMPLIANCE (MANAGEMENT) SCORING: Table 4.2** | | | | | |
| --- | --- | --- | --- | --- | --- |
| **Criterion** | | **Yes =1** | | **No = 0** | |
| **2. NON-TECHNICAL ASSESSMENT** | | | | | |
| **2A: Management Issues** | | | | | |
| **Assessment of strategic plans** | Does a strategic plan for the assessment of drinking water supply systems exist? | | **1** | |  |
|  | Are there preventive strategic plans for drinking water quality management? | | **1** | |  |
|  | Is there a strategic plan for recruitment of personnel per water treatment plant available? | | **1** | |  |
|  | Is there a strategic plan for operational monitoring and verification of drinking water system? | | **1** | |  |
|  | Is there a strategic plan for sharing and clarifying roles and responsibilities among all personnel involved in the production of safe drinking water quality? | | **1** | |  |
|  | Does a strategic plan for training and development of water plant personnel, and for community involvement and awareness exist? | | **1** | |  |
|  | Is there any funding strategic plan for implementation of drinking water quality management programmes? | | **1** | |  |
| **Assessment**  **of tact plans** | Does a tactical plan for handling emergencies including communication of such emergencies to communities exist? | | **1** | |  |
|  | Is the overall periodic overseeing of water care function performed? | | **1** | |  |
|  | Is there maintenance and asset management plans? | | **1** | |  |
| **Assessment of operational plans** | Is the attendance of process controllers (and in particular shift workers) at the treatment plant monitored? Is there a clock–in-system for the attendance? | | **1** | |  |
|  | Is the superintendent available to operators on a day-to-day basis? | | **1** | |  |
|  | Is there a substitute system plan put in place when one of the operators is ill or away attending a course? | | **1** | |  |
|  | Are the supervisors and process controllers aware of their job requirements? Are job descriptions available? | | **1** | |  |
| **Maximum possible score for *Management Issues*** | | | **14** | | |
| **Total score attained for *Management Issues*** | | | **14** | | |
| **Weight for *Management Issues*** | | | **0.1** | | |
| **Total weighted score for *Management Issues*** | | | **0.1** | | |

| **NON-TECHNICAL COMPLIANCE (MANAGEMENT) SCORING: Table 4.3** | | | |
| --- | --- | --- | --- |
| **Criterion** | | **Yes =1** | **No = 0** |
| **2. NON-TECHNICAL ASSESSMENT** | | | |
| **2B: Management Practices** | | | |
|  | Do the WSPs use existing local (Emanti/DWAF) or international management practices? Are they implementing them in their water treatment plants? | **1** |  |
|  | Are the WPSs able to understand the existing local or international practices and implementing them correctly? | **1** |  |
|  | When local or international practices are not available, are the WSPs able to draw their own criteria? | **1** |  |
| **Maximum possible score for *Management Practices*** | | **3** | |
| **Total score attained for *Management Practices*** | | **3** | |
| **Weight for *Management Practices*** | | **0.2** | |
| **Total weighted score for *Management Practices*** | | **0.2** | |

| **NON-TECHNICAL COMPLIANCE (MANAGEMENT) SCORING: Table 4.4** | | | | | |
| --- | --- | --- | --- | --- | --- |
| **Criterion** | | **Yes =1** | | **No = 0** | |
| **2. NON-TECHNICAL ASSESSMENT** | | | | | |
| **2C: Human Resources** | | | | | |
| **Personnel at the plant** | Is the number of personnel related to the size of the plant? | |  | | **0** |
|  | Are the needs of process controllers/supervisors and shift workers per plants adequate? | |  | | **0** |
|  | Are there specific guidelines/rules for shift workers and their deployment? | | **1** | |  |
|  | How many operators are there in total? How many shift workers? | | **1** | |  |
| **Qualifications of Personnel** | How many operators with qualifications in the DWAF categories? | | **1** | |  |
|  | Are personnel being correctly applied according to their qualifications? | | **1** | |  |
|  | Are there guidelines or formal rules for upgrading of qualifications? | | **1** | |  |
| **Training Needs** | Are there in service training programmes and policies? What is the in-service training policy of the WSA? | | **1** | |  |
|  | Do process controllers / supervisors / managers know what training opportunities and courses are available? | | **1** | |  |
|  | Are they sent on courses regularly? | |  | | **0** |
|  | Are there specific criteria or formal rules to be qualified for training? | |  | | **0** |
|  | Are there specific promotion requirement protocols in place? | | **1** | |  |
|  | Are there career advancement opportunities? | | **1** | |  |
| **Plant class** | Is the plant classified by DWAF? | |  | | **0** |
|  | Has it been done according to the new classification system? | |  | | **0** |
| **Working Conditions** | Are the responsibilities of process controllers, supervisors and plant managers in line with their salary? | |  | | **0** |
|  | What system is used for overtime management and payment? | | **1** | |  |
|  | What is the WSA policy on conflict management (internal and external)? | | **1** | |  |
|  | Is any medical assistance available for on the job injuries? | | **1** | |  |
|  | Can all workers participate in medical schemes? | | **1** | |  |
|  | Are the retirement/pension-aid benefits, the housing and travel allowance benefits available? | | **1** | |  |
|  | Are the leave benefits (vacation /study/ sick), and the bonus scheme available? | | **1** | |  |
|  | Are there any other incentives? | | **1** | |  |
|  | Are plant personnel satisfied with their working environment? | | **1** | |  |
|  | Are the relationships between supervisors and their subordinates good or poor? | | **1** | |  |
|  | Is there any performance indicators? | | **1** | |  |
| **Maximum possible score for *Human Resources*** | | | **26** | | |
| **Total score attained for *Human Resources*** | | | **19** | | |
| **Weight for *Human Resources*** | | | **0.2** | | |
| **Total weighted score for *Human Resources*** | | | **0.146** | | |

| **NON-TECHNICAL COMPLIANCE (MANAGEMENT) SCORING: Table 4.5** | | | |
| --- | --- | --- | --- |
| **Criterion** | | **Yes =1** | **No = 0** |
| **2. NON-TECHNICAL ASSESSMENT** | | | |
| **2D: Financial Systems** | | | |
| **Financial Systems Sharing in Place** | Which financial systems are in place? | **1** |  |
|  | Are there sufficient funds for operation, maintenance and overtime? | **1** |  |
|  | Do the size of the plants (small versus large) influence the allocation of funds? | **1** |  |
|  | Does the WSA’s budgeting system work in relation to emergency funds, scheduled upgrading/extensions, unscheduled rehabilitation/upgrading? | **1** |  |
| **Information Sharing in Place** | Are there communication channels and transparency between the financial section and the water care section? | **1** |  |
|  | Are regular meetings held? Who attends / chairs these meetings? | **1** |  |
|  | Are decisions taken at consensus / majority? | **1** |  |
|  | Are reports freely available? | **1** |  |
| **Procurement System** | Is the procurement system transparent? | **1** |  |
|  | Is the procurement system open to all? | **1** |  |
|  | Is the water sector involved in the adjudication of tenders? | **1** |  |
| **Maximum possible score for *Financial Systems*** | | **11** | |
| **Total score attained for *Financial Systems*** | | **11** | |
| **Weight for *Financial Systems*** | | **0.1** | |
| **Total weighted score for *Financial Systems*** | | **0.1** | |

| **NON-TECHNICAL COMPLIANCE (MANAGEMENT) SCORING: Table 4.6** | | | |
| --- | --- | --- | --- |
| **Criterion** | | **Yes =1** | **No = 0** |
| **2. NON-TECHNICAL ASSESSMENT** | | | |
| **2E: Communication Systems** | | | |
| **Internal** | Are there sufficient internal communication channels in place, including the needs and shortcomings? | **1** |  |
|  | Are these communication channels effective? | **1** |  |
|  | Is the communication between supervisors and process controllers good or poor? | **1** |  |
|  | Is there any interaction between the maintenance team and the rest of personnel in the water treatment plant? | **1** |  |
|  | Is the communication between management and lower level process controllers good or poor? | **1** |  |
| **External** | Are there sufficient communication channels with other Municipalities and/or Departments? | **1** |  |
|  | Are there sufficient communication channels with consumers? | **1** |  |
|  | Are these external communication channels effective, good or poor? | **1** |  |
| **Maximum possible score for *Communication Systems*** | | **8** | |
| **Total score attained for *Communication Systems*** | | **8** | |
| **Weight for *Communication Systems*** | | **0.2** | |
| **Total weighted score for *Communication Systems*** | | **0.2** | |

| **NON-TECHNICAL COMPLIANCE (MANAGEMENT) SCORING: Table 4.7** | | | |
| --- | --- | --- | --- |
| **Criterion** | | **Yes =1** | **No = 0** |
| **2. NON-TECHNICAL ASSESSMENT** | | | |
| **2F: Audit of safety, health and environmental quality** | | | |
| **Safety Matters** | Are there water and safety management procedures for normal operation, incidents and emergency situations? | **1** |  |
|  | Is there any safety plan at water treatment plants including the safety of process controllers on duties? | **1** |  |
|  | Are there emergency plans in place for chlorine leaks? | **1** |  |
|  | Are there hazardous chemicals and ablution facilities, appropriate fire extinguishers location? | **1** |  |
|  | Are safety meetings held regularly and who attends such meetings? | **1** |  |
| **Health Aspects** | Are there representatives for health and safety of consumers? If yes, are they effective? | **1** |  |
|  | Are there control measures that identify risks and ensure that health based- targets are met in terms of providing safe drinking water to all consumers? | **1** |  |
|  | Are there emergency measures in place for water quality health impact? | **1** |  |
| **Environmental Quality** | Are process controllers satisfied with their working environment? | **1** |  |
|  | Is the working environment appropriate for improving or accelerating the service delivery? | **1** |  |
|  | Is there any strategic plan to improve the quality of the working environment? | **1** |  |
| **Maximum possible score for *SHEQ*** | | **11** | |
| **Total score attained for *SHEQ*** | | **11** | |
| **Weight for *SHEQ*** | | **0.15** | |
| **Total weighted score for *SHEQ*** | | **0.15** | |

| **NON-TECHNICAL COMPLIANCE (MANAGEMENT) SCORING: Table 4.8** | | | |
| --- | --- | --- | --- |
| **Criterion** | | **Yes =1** | **No = 0** |
| **2. NON-TECHNICAL ASSESSMENT** | | | |
| **2G: Community involvement and awareness** | | | |
| **Community Involvement** | Are there protocols involved communities in decision-making? |  | **0** |
|  | Is there an effective reporting system for communities to assist in more rapid response to any water quality incident? |  | **0** |
|  | Is there a Consumer Service to which non-compliance can be reported? | **1** |  |
| **Community Awareness** | Is there a mechanism system to receive and actively address community complaints in a timely fashion? | **1** |  |
|  | Are there procedures for promptly advising of any significant incidents within the drinking water supply including notification of the public health authority? | **1** |  |
|  | Is there any water quality information system available to consumers through annual reports and in the internet? | **1** |  |
| **Maximum possible score for *Community Involvement*** | | **6** | |
| **Total score attained for *Community Involvement*** | | **4** | |
| **Weight for *Community Involvement*** | | **0.05** | |
| **Total weighted score for *Community Involvement*** | | **0.033** | |

| **TABLE 4.90**  **NON-TECHNICAL (MANAGEMENT) COMPLIANCE SCORING:**  **Total Weighted Scoring for Mutale Regional water treatment plant 92.9 %** | |
| --- | --- |
| **Compliance Criterion** | **Weighted Score** |
| **2. NON-TECHNICAL ASSESSMENT** | |
| **2A: Management Issues** | **0.1** |
| **2B: Management Practices** | **0.2** |
| **2C: Human Resources** | **0.146** |
| **2D: Financial Systems** | **0.1** |
| **2E: Communication Systems** | **0.2** |
| **2F: Safety, Health and Environmental Quality** | **0.15** |
| **2G: Community Involvement and Awareness** | **0.033** |
| **TOTAL WEIGHTED SCORE** | **0.929** |

| **TABLE 4.10**  **NON-TECHNICAL (MANAGEMENT) COMPLIANCE RATING:** | |
| --- | --- |
| **Total Weighted Score** | **Rating Description** |
| **0 – 50** | ***Class 3 Compliance*:**  Total non-compliance; serious and immediate intervention required (TAC) |
| **50 – 90** | ***Class 2 Compliance:***  Serious challenges requiring attention and improvement |
| **90 – 100** | ***Class 1 Compliance:***  Acceptable compliance |

**TSHEDZA Package Plant**

**19 August 2009**

| **TABLE 5.1**  **NON-TECHNICAL (MANAGEMENT) COMPLIANCE SCORING** | |
| --- | --- |
| **Criterion** | **Weight** |
| **2. NON-TECHNICAL ASSESSMENT** | |
| **2A: Management Issues** | **0.1** |
| **2B: Management Practices** | **0.2** |
| **2C: Human Resources** | **0.2** |
| **2D: Financial Systems** | **0.1** |
| **2E: Communication Systems** | **0.2** |
| **2F: Safety, Health and Environmental Quality** | **0.15** |
| **2G: Community Involvement and Awareness** | **0.05** |
| **TOTAL** | **1.0** |

| **NON-TECHNICAL COMPLIANCE (MANAGEMENT) SCORING: Table 5.2** | | | | | |
| --- | --- | --- | --- | --- | --- |
| **Criterion** | | **Yes =1** | | **No = 0** | |
| **2. NON-TECHNICAL ASSESSMENT** | | | | | |
| **2A: Management Issues** | | | | | |
| **Assessment of strategic plans** | Does a strategic plan for the assessment of drinking water supply systems exist? | | **1** | |  |
|  | Are there preventive strategic plans for drinking water quality management? | | **1** | |  |
|  | Is there a strategic plan for recruitment of personnel per water treatment plant available? | | **1** | |  |
|  | Is there a strategic plan for operational monitoring and verification of drinking water system? | | **1** | |  |
|  | Is there a strategic plan for sharing and clarifying roles and responsibilities among all personnel involved in the production of safe drinking water quality? | | **1** | |  |
|  | Does a strategic plan for training and development of water plant personnel, and for community involvement and awareness exist? | | **1** | |  |
|  | Is there any funding strategic plan for implementation of drinking water quality management programmes? | | **1** | |  |
| **Assessment of tact plans** | Does a tactical plan for handling emergencies including communication of such emergencies to communities exist? | | **1** | |  |
|  | Is the overall periodic overseeing of water care function performed? | | **1** | |  |
|  | Is there maintenance and asset management plans? | |  | | **0** |
| **Assessment of operational plans** | Is the attendance of process controllers (and in particular shift workers) at the treatment plant monitored? Is there a clock–in-system for the attendance? | | **1** | |  |
|  | Is the superintendent available to operators on a day-to-day basis? | | **1** | |  |
|  | Is there a substitute system plan put in place when one of the operators is ill or away attending a course? | | **1** | |  |
|  | Are the supervisors and process controllers aware of their job requirements? Are job descriptions available? | | **1** | |  |
| **Maximum possible score for *Management Issues*** | | | **14** | | |
| **Total score attained for *Management Issues*** | | | **13** | | |
| **Weight for *Management Issues*** | | | **0.1** | | |
| **Total weighted score for *Management Issues*** | | |  | | |

| **NON-TECHNICAL COMPLIANCE (MANAGEMENT) SCORING: Table 5.3** | | | |
| --- | --- | --- | --- |
| **Criterion** | | **Yes =1** | **No = 0** |
| **2. NON-TECHNICAL ASSESSMENT** | | | |
| **2B: Management Practices** | | | |
|  | Do the WSPs use existing local (Emanti/DWAF) or international management practices? Are they implementing them in their water treatment plants? | **1** |  |
|  | Are the WPSs able to understand the existing local or international practices and implementing them correctly? | **1** |  |
|  | When local or international practices are not available, are the WSPs able to draw their own criteria? | **1** |  |
| **Maximum possible score for *Management Practices*** | | **3** | |
| **Total score attained for *Management Practices*** | | **3** | |
| **Weight for *Management Practices*** | | **0.2** | |
| **Total weighted score for *Management Practices*** | |  | |

| **NON-TECHNICAL COMPLIANCE (MANAGEMENT) SCORING: Table 5.4** | | | | | |
| --- | --- | --- | --- | --- | --- |
| **Criterion** | | **Yes =1** | | **No = 0** | |
| **2. NON-TECHNICAL ASSESSMENT** | | | | | |
| **2C: Human Resources** | | | | | |
| **Personnel at the plant** | Is the number of personnel related to the size of the plant? | |  | | **0** |
|  | Are the needs of process controllers/supervisors and shift workers per plants adequate? | |  | | **0** |
|  | Are there specific guidelines/rules for shift workers and their deployment? | |  | | **0** |
|  | How many operators are there in total? How many shift workers? | | **1** | |  |
| **Qualifications of Personnel** | How many operators with qualifications in the DWAF categories? | | **1** | |  |
|  | Are personnel being correctly applied according to their qualifications? | | **1** | |  |
|  | Are there guidelines or formal rules for upgrading of qualifications? | | **1** | |  |
| **Training Needs** | Are there in service training programmes and policies? What is the in-service training policy of the WSA? | | **1** | |  |
|  | Do process controllers / supervisors / managers know what training opportunities and courses are available? | | **1** | |  |
|  | Are they sent on courses regularly? | | **1** | |  |
|  | Are there specific criteria or formal rules to be qualified for training? | | **1** | |  |
|  | Are there specific promotion requirement protocols in place? | | **1** | |  |
|  | Are there career advancement opportunities? | | **1** | |  |
| **Plant class** | Is the plant classified by DWAF? | |  | | **0** |
|  | Has it been done according to the new classification system? | |  | | **0** |
| **Working Conditions** | Are the responsibilities of process controllers, supervisors and plant managers in line with their salary? | |  | | **0** |
|  | What system is used for overtime management and payment? | | **1** | |  |
|  | What is the WSA policy on conflict management (internal and external)? | | **1** | |  |
|  | Is any medical assistance available for on the job injuries? | | **1** | |  |
|  | Can all workers participate in medical schemes? | | **1** | |  |
|  | Are the retirement/pension-aid benefits, the housing and travel allowance benefits available? | | **1** | |  |
|  | Are the leave benefits (vacation /study/ sick), and the bonus scheme available? | | **1** | |  |
|  | Are there any other incentives? | | **1** | |  |
|  | Are plant personnel satisfied with their working environment? | | **1** | |  |
|  | Are the relationships between supervisors and their subordinates good or poor? | | **1** | |  |
|  | Is there any performance indicators? | | **1** | |  |
| **Maximum possible score for *Human Resources*** | | | **27** | | |
| **Total score attained for *Human Resources*** | | | **21** | | |
| **Weight for *Human Resources*** | | | **0.2** | | |
| **Total weighted score for *Human Resources*** | | |  | | |

| **NON-TECHNICAL COMPLIANCE (MANAGEMENT) SCORING: Table 5.5** | | | |
| --- | --- | --- | --- |
| **Criterion** | | **Yes =1** | **No = 0** |
| **2. NON-TECHNICAL ASSESSMENT** | | | |
| **2D: Financial Systems** | | | |
| **Financial Systems Sharing in Place** | Which financial systems are in place? | **1** |  |
|  | Are there sufficient funds for operation, maintenance and overtime? | **1** |  |
|  | Do the size of the plants (small versus large) influence the allocation of funds? | **1** |  |
|  | Does the WSA’s budgeting system work in relation to emergency funds, scheduled upgrading/extensions, unscheduled rehabilitation/upgrading? | **1** |  |
| **Information Sharing in Place** | Are there communication channels and transparency between the financial section and the water care section? | **1** |  |
|  | Are regular meetings held? Who attends / chairs these meetings? | **1** |  |
|  | Are decisions taken at consensus / majority? | **1** |  |
|  | Are reports freely available? | **1** |  |
| **Procurement System** | Is the procurement system transparent? | **1** |  |
|  | Is the procurement system open to all? | **1** |  |
|  | Is the water sector involved in the adjudication of tenders? | **1** |  |
| **Maximum possible score for *Financial Systems*** | | **11** | |
| **Total score attained for *Financial Systems*** | | **11** | |
| **Weight for *Financial Systems*** | | **0.1** | |
| **Total weighted score for *Financial Systems*** | |  | |

| **NON-TECHNICAL COMPLIANCE (MANAGEMENT) SCORING: Table 5.6** | | | |
| --- | --- | --- | --- |
| **Criterion** | | **Yes =1** | **No = 0** |
| **2. NON-TECHNICAL ASSESSMENT** | | | |
| **2E: Communication Systems** | | | |
| **Internal** | Are there sufficient internal communication channels in place, including the needs and shortcomings? | **1** |  |
|  | Are these communication channels effective? | **1** |  |
|  | Is the communication between supervisors and process controllers good or poor? | **1** |  |
|  | Is there any interaction between the maintenance team and the rest of personnel in the water treatment plant? | **1** |  |
|  | Is the communication between management and lower level process controllers good or poor? | **1** |  |
| **External** | Are there sufficient communication channels with other Municipalities and/or Departments? | **1** |  |
|  | Are there sufficient communication channels with consumers? | **1** |  |
|  | Are these external communication channels effective, good or poor? | **1** |  |
| **Maximum possible score for *Communication Systems*** | | **8** | |
| **Total score attained for *Communication Systems*** | | **8** | |
| **Weight for *Communication Systems*** | | **0.2** | |
| **Total weighted score for *Communication Systems*** | |  | |

| **NON-TECHNICAL COMPLIANCE (MANAGEMENT) SCORING: Table 5.7** | | | |
| --- | --- | --- | --- |
| **Criterion** | | **Yes =1** | **No = 0** |
| **2. NON-TECHNICAL ASSESSMENT** | | | |
| **2F: Audit of safety, health and environmental quality** | | | |
| **Safety Matters** | Are there water and safety management procedures for normal operation, incidents and emergency situations? | **1** |  |
|  | Is there any safety plan at water treatment plants including the safety of process controllers on duties? | **1** |  |
|  | Are there emergency plans in place for chlorine leaks? | **1** |  |
|  | Are there hazardous chemicals and ablution facilities, appropriate fire extinguishers location? | **1** |  |
|  | Are safety meetings held regularly and who attends such meetings? | **1** |  |
| **Health Aspects** | Are there representatives for health and safety of consumers? If yes, are they effective? | **1** |  |
|  | Are there control measures that identify risks and ensure that health based- targets are met in terms of providing safe drinking water to all consumers? | **1** |  |
|  | Are there emergency measures in place for water quality health impact? | **1** |  |
| **Environmental Quality** | Are process controllers satisfied with their working environment? | **1** |  |
|  | Is the working environment appropriate for improving or accelerating the service delivery? | **1** |  |
|  | Is there any strategic plan to improve the quality of the working environment? | **1** |  |
| **Maximum possible score for *SHEQ*** | | **11** | |
| **Total score attained for *SHEQ*** | | **11** | |
| **Weight for *SHEQ*** | | **0.15** | |
| **Total weighted score for *SHEQ*** | |  | |

| **NON-TECHNICAL COMPLIANCE (MANAGEMENT) SCORING: Table 5.8** | | | |
| --- | --- | --- | --- |
| **Criterion** | | **Yes =1** | **No = 0** |
| **2. NON-TECHNICAL ASSESSMENT** | | | |
| **2G: Community involvement and awareness** | | | |
| **Community Involvement** | Are there protocols involved communities in decision-making? |  | **0** |
|  | Is there an effective reporting system for communities to assist in more rapid response to any water quality incident? |  | **0** |
|  | Is there a Consumer Service to which non-compliance can be reported? | **1** |  |
| **Community Awareness** | Is there a mechanism system to receive and actively address community complaints in a timely fashion? | **1** |  |
|  | Are there procedures for promptly advising of any significant incidents within the drinking water supply including notification of the public health authority? | **1** |  |
|  | Is there any water quality information system available to consumers through annual reports and in the internet? | **1** |  |
| **Maximum possible score for *Community Involvement*** | | **6** | |
| **Total score attained for *Community Involvement*** | | **4** | |
| **Weight for *Community Involvement*** | | **0.05** | |
| **Total weighted score for *Community Involvement*** | |  | |

| **TABLE 5.9**  **NON-TECHNICAL (MANAGEMENT) COMPLIANCE SCORING:**  **Total Weighted Scoring for Tshedza Package plant 93.1 %** | |
| --- | --- |
| **Compliance Criterion** | **Weighted Score** |
| **2. NON-TECHNICAL ASSESSMENT** | |
| **2A: Management Issues** | **0.0928** |
| **2B: Management Practices** | **0.2** |
| **2C: Human Resources** | **0.155** |
| **2D: Financial Systems** | **0.1** |
| **2E: Communication Systems** | **0.2** |
| **2F: Safety, Health and Environmental Quality** | **0.15** |
| **2G: Community Involvement and Awareness** | **0.033** |
| **TOTAL WEIGHTED SCORE** | **0.931** |

| **TABLE 5.10**  **NON-TECHNICAL (MANAGEMENT) COMPLIANCE RATING:** | |
| --- | --- |
| **Total Weighted Score** | **Rating Description** |
| **0 – 50** | ***Class 3 Compliance*:**  **Total non-compliance; serious and immediate intervention required (TAC)** |
| **50 – 90** | ***Class 2 Compliance:***  **Serious challenges requiring attention and improvement** |
| **90 – 100** | ***Class 1 Compliance:***  **Acceptable compliance** |

**Dzingahe Package Plant**

**19 August 2009**

| **TABLE 6.1**  **NON-TECHNICAL (MANAGEMENT) COMPLIANCE SCORING** | |
| --- | --- |
| **Criterion** | **Weight** |
| **2. NON-TECHNICAL ASSESSMENT** | |
| **2A: Management Issues** | **0.1** |
| **2B: Management Practices** | **0.2** |
| **2C: Human Resources** | **0.2** |
| **2D: Financial Systems** | **0.1** |
| **2E: Communication Systems** | **0.2** |
| **2F: Safety, Health and Environmental Quality** | **0.15** |
| **2G: Community Involvement and Awareness** | **0.05** |
| **TOTAL** | **1.0** |

| **NON-TECHNICAL COMPLIANCE (MANAGEMENT) SCORING: Table 6.2** | | | | | |
| --- | --- | --- | --- | --- | --- |
| **Criterion** | | **Yes =1** | | **No = 0** | |
| **2. NON-TECHNICAL ASSESSMENT** | | | | | |
| **2A: Management Issues** | | | | | |
| **Assessment of strategic plans** | Does a strategic plan for the assessment of drinking water supply systems exist? | | **1** | |  |
|  | Are there preventive strategic plans for drinking water quality management? | | **1** | |  |
|  | Is there a strategic plan for recruitment of personnel per water treatment plant available? | | **1** | |  |
|  | Is there a strategic plan for operational monitoring and verification of drinking water system? | | **1** | |  |
|  | Is there a strategic plan for sharing and clarifying roles and responsibilities among all personnel involved in the production of safe drinking water quality? | | **1** | |  |
|  | Does a strategic plan for training and development of water plant personnel, and for community involvement and awareness exist? | | **1** | |  |
|  | Is there any funding strategic plan for implementation of drinking water quality management programmes? | | **1** | |  |
| **Assm of tact plans** | Does a tactical plan for handling emergencies including communication of such emergencies to communities exist? | | **1** | |  |
|  | Is the overall periodic overseeing of water care function performed? | | **1** | |  |
|  | Is there maintenance and asset management plans? | |  | | **0** |
| **Assessment of operational plans** | Is the attendance of process controllers (and in particular shift workers) at the treatment plant monitored? Is there a clock–in-system for the attendance? | | **1** | |  |
|  | Is the superintendent available to operators on a day-to-day basis? | | **1** | |  |
|  | Is there a substitute system plan put in place when one of the operators is ill or away attending a course? | | **1** | |  |
|  | Are the supervisors and process controllers aware of their job requirements? Are job descriptions available? | | **1** | |  |
| **Maximum possible score for *Management Issues*** | | | **14** | | |
| **Total score attained for *Management Issues*** | | | **13** | | |
| **Weight for *Management Issues*** | | | **0.1** | | |
| **Total weighted score for *Management Issues*** | | | **0.0929** | | |

| **NON-TECHNICAL COMPLIANCE (MANAGEMENT) SCORING: Table 6.3** | | | |
| --- | --- | --- | --- |
| **Criterion** | | **Yes =1** | **No = 0** |
| **2. NON-TECHNICAL ASSESSMENT** | | | |
| **2B: Management Practices** | | | |
|  | Do the WSPs use existing local (Emanti/DWAF) or international management practices? Are they implementing them in their water treatment plants? | **1** |  |
|  | Are the WPSs able to understand the existing local or international practices and implementing them correctly? | **1** |  |
|  | When local or international practices are not available, are the WSPs able to draw their own criteria? | **1** |  |
| **Maximum possible score for *Management Practices*** | | **3** | |
| **Total score attained for *Management Practices*** | | **3** | |
| **Weight for *Management Practices*** | | **0.2** | |
| **Total weighted score for *Management Practices*** | | **0.2** | |

| **NON-TECHNICAL COMPLIANCE (MANAGEMENT) SCORING: Table 6.4** | | | | | |
| --- | --- | --- | --- | --- | --- |
| **Criterion** | | **Yes =1** | | **No = 0** | |
| **2. NON-TECHNICAL ASSESSMENT** | | | | | |
| **2C: Human Resources** | | | | | |
| **Personnel at the plant** | Is the number of personnel related to the size of the plant? | |  | | **0** |
|  | Are the needs of process controllers/supervisors and shift workers per plants adequate? | |  | | **0** |
|  | Are there specific guidelines/rules for shift workers and their deployment? | | **1** | |  |
|  | How many operators are there in total? How many shift workers? | | **1** | |  |
| **Qualifications of Personnel** | How many operators with qualifications in the DWAF categories? | | **1** | |  |
|  | Are personnel being correctly applied according to their qualifications? | | **1** | |  |
|  | Are there guidelines or formal rules for upgrading of qualifications? | | **1** | |  |
| **Training Needs** | Are there in service training programmes and policies? What is the in-service training policy of the WSA? | | **1** | |  |
|  | Do process controllers / supervisors / managers know what training opportunities and courses are available? | | **1** | |  |
|  | Are they sent on courses regularly? | | **1** | |  |
|  | Are there specific criteria or formal rules to be qualified for training? | | **1** | |  |
|  | Are there specific promotion requirement protocols in place? | | **1** | |  |
|  | Are there career advancement opportunities? | | **1** | |  |
| **Plant class** | Is the plant classified by DWAF? | |  | | **0** |
|  | Has it been done according to the new classification system? | |  | | **0** |
| **Working Conditions** | Are the responsibilities of process controllers, supervisors and plant managers in line with their salary? | |  | | **0** |
|  | What system is used for overtime management and payment? | | **1** | |  |
|  | What is the WSA policy on conflict management (internal and external)? | | **1** | |  |
|  | Is any medical assistance available for on the job injuries? | | **1** | |  |
|  | Can all workers participate in medical schemes? | | **1** | |  |
|  | Are the retirement/pension-aid benefits, the housing and travel allowance benefits available? | | **1** | |  |
|  | Are the leave benefits (vacation /study/ sick), and the bonus scheme available? | | **1** | |  |
|  | Are there any other incentives? | | **1** | |  |
|  | Are plant personnel satisfied with their working environment? | | **1** | |  |
|  | Are the relationships between supervisors and their subordinates good or poor? | | **1** | |  |
|  | Is there any performance indicators? | | **1** | |  |
| **Maximum possible score for *Human Resources*** | | | **27** | | |
| **Total score attained for *Human Resources*** | | | **22** | | |
| **Weight for *Human Resources*** | | | **0.2** | | |
| **Total weighted score for *Human Resources*** | | | **0.163** | | |

| **NON-TECHNICAL COMPLIANCE (MANAGEMENT) SCORING: Table 6.5** | | | |
| --- | --- | --- | --- |
| **Criterion** | | **Yes =1** | **No = 0** |
| **2. NON-TECHNICAL ASSESSMENT** | | | |
| **2D: Financial Systems** | | | |
| **Financial Systems Sharing in Place** | Which financial systems are in place? | **1** |  |
|  | Are there sufficient funds for operation, maintenance and overtime? |  | **0** |
|  | Do the size of the plants (small versus large) influence the allocation of funds? | **1** |  |
|  | Does the WSA’s budgeting system work in relation to emergency funds, scheduled upgrading/extensions, unscheduled rehabilitation/upgrading? | **1** |  |
| **Information Sharing in Place** | Are there communication channels and transparency between the financial section and the water care section? | **1** |  |
|  | Are regular meetings held? Who attends / chairs these meetings? | **1** |  |
|  | Are decisions taken at consensus / majority? | **1** |  |
|  | Are reports freely available? | **1** |  |
| **Procurement System** | Is the procurement system transparent? | **1** |  |
|  | Is the procurement system open to all? | **1** |  |
|  | Is the water sector involved in the adjudication of tenders? | **1** |  |
| **Maximum possible score for *Financial Systems*** | | **11** | |
| **Total score attained for *Financial Systems*** | | **10** | |
| **Weight for *Financial Systems*** | | **0.1** | |
| **Total weighted score for *Financial Systems*** | | **0.091** | |

| **NON-TECHNICAL COMPLIANCE (MANAGEMENT) SCORING: Table 6.6** | | | |
| --- | --- | --- | --- |
| **Criterion** | | **Yes =1** | **No = 0** |
| **2. NON-TECHNICAL ASSESSMENT** | | | |
| **2E: Communication Systems** | | | |
| **Internal** | Are there sufficient internal communication channels in place, including the needs and shortcomings? | **1** |  |
|  | Are these communication channels effective? | **1** |  |
|  | Is the communication between supervisors and process controllers good or poor? | **1** |  |
|  | Is there any interaction between the maintenance team and the rest of personnel in the water treatment plant? | **1** |  |
|  | Is the communication between management and lower level process controllers good or poor? | **1** |  |
| **External** | Are there sufficient communication channels with other Municipalities and/or Departments? | **1** |  |
|  | Are there sufficient communication channels with consumers? | **1** |  |
|  | Are these external communication channels effective, good or poor? | **1** |  |
| **Maximum possible score for *Communication Systems*** | | **8** | |
| **Total score attained for *Communication Systems*** | | **8** | |
| **Weight for *Communication Systems*** | | **0.2** | |
| **Total weighted score for *Communication Systems*** | | **0.2** | |

| **NON-TECHNICAL COMPLIANCE (MANAGEMENT) SCORING: Table 6.7** | | | |
| --- | --- | --- | --- |
| **Criterion** | | **Yes =1** | **No = 0** |
| **2. NON-TECHNICAL ASSESSMENT** | | | |
| **2F: Audit of safety, health and environmental quality** | | | |
| **Safety Matters** | Are there water and safety management procedures for normal operation, incidents and emergency situations? | **1** |  |
|  | Is there any safety plan at water treatment plants including the safety of process controllers on duties? |  | **0** |
|  | Are there emergency plans in place for chlorine leaks? | **1** |  |
|  | Are there hazardous chemicals and ablution facilities, appropriate fire extinguishers location? | **1** |  |
|  | Are safety meetings held regularly and who attends such meetings? | **1** |  |
| **Health Aspects** | Are there representatives for health and safety of consumers? If yes, are they effective? |  | **0** |
|  | Are there control measures that identify risks and ensure that health based- targets are met in terms of providing safe drinking water to all consumers? | **1** |  |
|  | Are there emergency measures in place for water quality health impact? | **1** |  |
| **Environmental Quality** | Are process controllers satisfied with their working environment? | **1** |  |
|  | Is the working environment appropriate for improving or accelerating the service delivery? | **1** |  |
|  | Is there any strategic plan to improve the quality of the working environment? | **1** |  |
| **Maximum possible score for *SHEQ*** | | **11** | |
| **Total score attained for *SHEQ*** | | **9** | |
| **Weight for *SHEQ*** | | **0.15** | |
| **Total weighted score for *SHEQ*** | | **0.123** | |

| **NON-TECHNICAL COMPLIANCE (MANAGEMENT) SCORING: Table 6.8** | | | |
| --- | --- | --- | --- |
| **Criterion** | | **Yes =1** | **No = 0** |
| **2. NON-TECHNICAL ASSESSMENT** | | | |
| **2G: Community involvement and awareness** | | | |
| **Community Involvement** | Are there protocols involved communities in decision-making? |  | **0** |
|  | Is there an effective reporting system for communities to assist in more rapid response to any water quality incident? |  | **0** |
|  | Is there a Consumer Service to which non-compliance can be reported? |  | **0** |
| **Community Awareness** | Is there a mechanism system to receive and actively address community complaints in a timely fashion? | **1** |  |
|  | Are there procedures for promptly advising of any significant incidents within the drinking water supply including notification of the public health authority? | **1** |  |
|  | Is there any water quality information system available to consumers through annual reports and in the internet? | **1** |  |
| **Maximum possible score for *Community Involvement*** | | **6** | |
| **Total score attained for *Community Involvement*** | | **3** | |
| **Weight for *Community Involvement*** | | **0.05** | |
| **Total weighted score for *Community Involvement*** | | **0.25** | |

| **TABLE 6.9**  **NON-TECHNICAL (MANAGEMENT) COMPLIANCE SCORING:**  **Total Weighted Scoring for Dzingahe Package plant 89.5 %** | |
| --- | --- |
| **Compliance Criterion** | **Weighted Score** |
| **2. NON-TECHNICAL ASSESSMENT** | |
| **2A: Management Issues** | **0.093** |
| **2B: Management Practices** | **0.2** |
| **2C: Human Resources** | **0.163** |
| **2D: Financial Systems** | **0.091** |
| **2E: Communication Systems** | **0.2** |
| **2F: Safety, Health and Environmental Quality** | **0.123** |
| **2G: Community Involvement and Awareness** | **0.025** |
| **TOTAL WEIGHTED SCORE** | **0.895** |

| **TABLE 6.10**  **NON-TECHNICAL (MANAGEMENT) COMPLIANCE RATING:** | |
| --- | --- |
| **Total Weighted Score** | **Rating Description** |
| **0 – 50** | ***Class 3 Compliance*:**  Total non-compliance; serious and immediate intervention required (TAC) |
| **50 – 90** | ***Class 2 Compliance:***  Serious challenges requiring attention and improvement |
| **90 – 100** | ***Class 1 Compliance:***  Acceptable compliance |

**MALAMULELE TREATMENT WATER PLANT**

**10 May 2011**

| **TABLE 7.1**  **NON-TECHNICAL (MANAGEMENT) COMPLIANCE SCORING** | |
| --- | --- |
| **Criterion** | **Weight** |
| **2. NON-TECHNICAL ASSESSMENT** | |
| **2A: Management Issues** | **0.1** |
| **2B: Management Practices** | **0.2** |
| **2C: Human Resources** | **0.2** |
| **2D: Financial Systems** | **0.1** |
| **2E: Communication Systems** | **0.2** |
| **2F: Safety, Health and Environmental Quality** | **0.15** |
| **2G: Community Involvement and Awareness** | **0.05** |
| **TOTAL** | **1.0** |

| **NON-TECHNICAL COMPLIANCE (MANAGEMENT) SCORING: Table 7.2** | | | | | |
| --- | --- | --- | --- | --- | --- |
| **Criterion** | | **Yes =1** | | **No = 0** | |
| **2. NON-TECHNICAL ASSESSMENT** | | | | | |
| **2A: Management Issues** | | | | | |
| **Assessment of strategic plans** | Does a strategic plan for the assessment of drinking water supply systems exist? | | **1** | |  |
|  | Are there preventive strategic plans for drinking water quality management? | | **1** | |  |
|  | Is there a strategic plan for recruitment of personnel per water treatment plant available? | | **1** | |  |
|  | Is there a strategic plan for operational monitoring and verification of drinking water system? | | **1** | |  |
|  | Is there a strategic plan for sharing and clarifying roles and responsibilities among all personnel involved in the production of safe drinking water quality? | | **1** | |  |
|  | Does a strategic plan for training and development of water plant personnel, and for community involvement and awareness exist? | | **1** | |  |
|  | Is there any funding strategic plan for implementation of drinking water quality management programmes? | | **1** | |  |
| **Assessment of tact plans** | Does a tactical plan for handling emergencies including communication of such emergencies to communities exist? | | **1** | |  |
|  | Is the overall periodic overseeing of water care function performed? | | **1** | |  |
|  | Is there maintenance and asset management plans? | | **1** | |  |
| **Assessment of operational plans** | Is the attendance of process controllers (and in particular shift workers) at the treatment plant monitored? Is there a clock–in-system for the attendance? | | **1** | |  |
|  | Is the superintendent available to operators on a day-to-day basis? | | **1** | |  |
|  | Is there a substitute system plan put in place when one of the operators is ill or away attending a course? | | **1** | |  |
|  | Are the supervisors and process controllers aware of their job requirements? Are job descriptions available? | | **1** | |  |
| **Maximum possible score for *Management Issues*** | | | **14** | | |
| **Total score attained for *Management Issues*** | | | **14** | | |
| **Weight for *Management Issues*** | | | **0.1** | | |
| **Total weighted score for *Management Issues*** | | | **0.1** | | |

| **NON-TECHNICAL COMPLIANCE (MANAGEMENT) SCORING: Table 7.3** | | | |
| --- | --- | --- | --- |
| **Criterion** | | **Yes =1** | **No = 0** |
| **2. NON-TECHNICAL ASSESSMENT** | | | |
| **2B: Management Practices** | | | |
|  | Do the WSPs use existing local (Emanti/DWAF) or international management practices? Are they implementing them in their water treatment plants? | **1** |  |
|  | Are the WPSs able to understand the existing local or international practices and implementing them correctly? | **1** |  |
|  | When local or international practices are not available, are the WSPs able to draw their own criteria? | **1** |  |
| **Maximum possible score for *Management Practices*** | | **3** | |
| **Total score attained for *Management Practices*** | | **3** | |
| **Weight for *Management Practices*** | | **0.2** | |
| **Total weighted score for *Management Practices*** | | **0.2** | |

| **NON-TECHNICAL COMPLIANCE (MANAGEMENT) SCORING: Table 7.4** | | | | | |
| --- | --- | --- | --- | --- | --- |
| **Criterion** | | **Yes =1** | | **No = 0** | |
| **2. NON-TECHNICAL ASSESSMENT** | | | | | |
| **2C: Human Resources** | | | | | |
| **Personnel at the plant** | Is the number of personnel related to the size of the plant? | |  | | **0** |
|  | Are the needs of process controllers/supervisors and shift workers per plants adequate? | |  | | **0** |
|  | Are there specific guidelines/rules for shift workers and their deployment? | | **1** | |  |
|  | How many operators are there in total? How many shift workers? | | **1** | |  |
| **Qualifications of Personnel** | How many operators with qualifications in the DWAF categories? | | **1** | |  |
|  | Are personnel being correctly applied according to their qualifications? | | **1** | |  |
|  | Are there guidelines or formal rules for upgrading of qualifications? | | **1** | |  |
| **Training Needs** | Are there in service training programmes and policies? What is the in-service training policy of the WSA? | | **1** | |  |
|  | Do process controllers / supervisors / managers know what training opportunities and courses are available? | | **1** | |  |
|  | Are they sent on courses regularly? | | **1** | |  |
|  | Are there specific criteria or formal rules to be qualified for training? | | **1** | |  |
|  | Are there specific promotion requirement protocols in place? | | **1** | |  |
|  | Are there career advancement opportunities? | | **1** | |  |
| **Plant class** | Is the plant classified by DWAF? | | **1** | |  |
|  | Has it been done according to the new classification system? | | **1** | |  |
| **Working Conditions** | Are the responsibilities of process controllers, supervisors and plant managers in line with their salary? | |  | | **0** |
|  | What system is used for overtime management and payment? | | **1** | |  |
|  | What is the WSA policy on conflict management (internal and external)? | | **1** | |  |
|  | Is any medical assistance available for on the job injuries? | | **1** | |  |
|  | Can all workers participate in medical schemes? | | **1** | |  |
|  | Are the retirement/pension-aid benefits, the housing and travel allowance benefits available? | | **1** | |  |
|  | Are the leave benefits (vacation /study/ sick), and the bonus scheme available? | | **1** | |  |
|  | Are there any other incentives? | | **1** | |  |
|  | Are plant personnel satisfied with their working environment? | | **1** | |  |
|  | Are the relationships between supervisors and their subordinates good or poor? | | **1** | |  |
|  | Is there any performance indicators? | | **1** | |  |
| **Maximum possible score for *Human Resources*** | | | **27** | | |
| **Total score attained for *Human Resources*** | | | **24** | | |
| **Weight for *Human Resources*** | | | **0.2** | | |
| **Total weighted score for *Human Resources*** | | | **0.178** | | |

| **NON-TECHNICAL COMPLIANCE (MANAGEMENT) SCORING: Table 7.5** | | | |
| --- | --- | --- | --- |
| **Criterion** | | **Yes =1** | **No = 0** |
| **2. NON-TECHNICAL ASSESSMENT** | | | |
| **2D: Financial Systems** | | | |
| **Financial Systems Sharing in Place** | Which financial systems are in place? | **1** |  |
|  | Are there sufficient funds for operation, maintenance and overtime? | **1** |  |
|  | Do the size of the plants (small versus large) influence the allocation of funds? | **1** |  |
|  | Does the WSA’s budgeting system work in relation to emergency funds, scheduled upgrading/extensions, unscheduled rehabilitation/upgrading? | **1** |  |
| **Information Sharing in Place** | Are there communication channels and transparency between the financial section and the water care section? | **1** |  |
|  | Are regular meetings held? Who attends / chairs these meetings? | **1** |  |
|  | Are decisions taken at consensus / majority? | **1** |  |
|  | Are reports freely available? | **1** |  |
| **Procurement System** | Is the procurement system transparent? | **1** |  |
|  | Is the procurement system open to all? | **1** |  |
|  | Is the water sector involved in the adjudication of tenders? | **1** |  |
| **Maximum possible score for *Financial Systems*** | | **11** | |
| **Total score attained for *Financial Systems*** | | **11** | |
| **Weight for *Financial Systems*** | | **0.1** | |
| **Total weighted score for *Financial Systems*** | | **0.1** | |

| **NON-TECHNICAL COMPLIANCE (MANAGEMENT) SCORING: Table 7.6** | | | |
| --- | --- | --- | --- |
| **Criterion** | | **Yes =1** | **No = 0** |
| **2. NON-TECHNICAL ASSESSMENT** | | | |
| **2E: Communication Systems** | | | |
| **Internal** | Are there sufficient internal communication channels in place, including the needs and shortcomings? | **1** |  |
|  | Are these communication channels effective? | **1** |  |
|  | Is the communication between supervisors and process controllers good or poor? | **1** |  |
|  | Is there any interaction between the maintenance team and the rest of personnel in the water treatment plant? | **1** |  |
|  | Is the communication between management and lower level process controllers good or poor? | **1** |  |
| **External** | Are there sufficient communication channels with other Municipalities and/or Departments? | **1** |  |
|  | Are there sufficient communication channels with consumers? | **1** |  |
|  | Are these external communication channels effective, good or poor? | **1** |  |
| **Maximum possible score for *Communication Systems*** | | **8** | |
| **Total score attained for *Communication Systems*** | | **8** | |
| **Weight for *Communication Systems*** | | **0.2** | |
| **Total weighted score for *Communication Systems*** | | **0.2** | |

| **NON-TECHNICAL COMPLIANCE (MANAGEMENT) SCORING: Table 7.7** | | | |
| --- | --- | --- | --- |
| **Criterion** | | **Yes =1** | **No = 0** |
| **2. NON-TECHNICAL ASSESSMENT** | | | |
| **2F: Audit of safety, health and environmental quality** | | | |
| **Safety Matters** | Are there water and safety management procedures for normal operation, incidents and emergency situations? | **1** |  |
|  | Is there any safety plan at water treatment plants including the safety of process controllers on duties? | **1** |  |
|  | Are there emergency plans in place for chlorine leaks? | **1** |  |
|  | Are there hazardous chemicals and ablution facilities, appropriate fire extinguishers location? | **1** |  |
|  | Are safety meetings held regularly and who attends such meetings? | **1** |  |
| **Health Aspects** | Are there representatives for health and safety of consumers? If yes, are they effective? | **1** |  |
|  | Are there control measures that identify risks and ensure that health based- targets are met in terms of providing safe drinking water to all consumers? | **1** |  |
|  | Are there emergency measures in place for water quality health impact? | **1** |  |
| **Environmental Quality** | Are process controllers satisfied with their working environment? | **1** |  |
|  | Is the working environment appropriate for improving or accelerating the service delivery? | **1** |  |
|  | Is there any strategic plan to improve the quality of the working environment? | **1** |  |
| **Maximum possible score for *SHEQ*** | | **11** | |
| **Total score attained for *SHEQ*** | | **11** | |
| **Weight for *SHEQ*** | | **0.15** | |
| **Total weighted score for *SHEQ*** | | **0.15** | |

| **NON-TECHNICAL COMPLIANCE (MANAGEMENT) SCORING: Table 7.8** | | | |
| --- | --- | --- | --- |
| **Criterion** | | **Yes =1** | **No = 0** |
| **2. NON-TECHNICAL ASSESSMENT** | | | |
| **2G: Community involvement and awareness** | | | |
| **Community Involvement** | Are there protocols involved communities in decision-making? |  | **0** |
|  | Is there an effective reporting system for communities to assist in more rapid response to any water quality incident? | **1** |  |
|  | Is there a Consumer Service to which non-compliance can be reported? | **1** |  |
| **Community Awareness** | Is there a mechanism system to receive and actively address community complaints in a timely fashion? | **1** |  |
|  | Are there procedures for promptly advising of any significant incidents within the drinking water supply including notification of the public health authority? | **1** |  |
|  | Is there any water quality information system available to consumers through annual reports and in the internet? | **1** |  |
| **Maximum possible score for *Community Involvement*** | | **6** | |
| **Total score attained for *Community Involvement*** | | **5** | |
| **Weight for *Community Involvement*** | | **0.05** | |
| **Total weighted score for *Community Involvement*** | | **0.042** | |

| **TABLE 7.9**  **NON-TECHNICAL (MANAGEMENT) COMPLIANCE SCORING:**  **Total Weighted Scoring for Malamulele treatment water plant 97.0 %** | |
| --- | --- |
| **Compliance Criterion** | **Weighted Score** |
| **2. NON-TECHNICAL ASSESSMENT** | |
| **2A: Management Issues** | **0.1** |
| **2B: Management Practices** | **0.2** |
| **2C: Human Resources** | **0.178** |
| **2D: Financial Systems** | **0.1** |
| **2E: Communication Systems** | **0.2** |
| **2F: Safety, Health and Environmental Quality** | **0.15** |
| **2G: Community Involvement and Awareness** | **0.042** |
| **TOTAL WEIGHTED SCORE** | **0.97** |

| **TABLE 7.10**  **NON-TECHNICAL (MANAGEMENT) COMPLIANCE RATING:** | |
| --- | --- |
| **Total Weighted Score** | **Rating Description** |
| **0 – 50** | ***Class 3 Compliance*:**  Total non-compliance; serious and immediate intervention required (TAC) |
| **50 – 90** | ***Class 2 Compliance:***  Serious challenges requiring attention and improvement |
| **90 – 100** | ***Class 1 Compliance:***  Acceptable compliance |

**Vondo treatment Plant**

**13 May 2011**

| **TABLE 8.1**  **NON-TECHNICAL (MANAGEMENT) COMPLIANCE SCORING** | |
| --- | --- |
| **Criterion** | **Weight** |
| **2. NON-TECHNICAL ASSESSMENT** | |
| **2A: Management Issues** | **0.1** |
| **2B: Management Practices** | **0.2** |
| **2C: Human Resources** | **0.2** |
| **2D: Financial Systems** | **0.1** |
| **2E: Communication Systems** | **0.2** |
| **2F: Safety, Health and Environmental Quality** | **0.15** |
| **2G: Community Involvement and Awareness** | **0.05** |
| **TOTAL** | **1.0** |

| **NON-TECHNICAL COMPLIANCE (MANAGEMENT) SCORING: Table 8.2** | | | | | |
| --- | --- | --- | --- | --- | --- |
| **Criterion** | | **Yes =1** | | **No = 0** | |
| **2. NON-TECHNICAL ASSESSMENT** | | | | | |
| **2A: Management Issues** | | | | | |
| **Assessment of strategic plans** | Does a strategic plan for the assessment of drinking water supply systems exist? | | **1** | |  |
|  | Are there preventive strategic plans for drinking water quality management? | | **1** | |  |
|  | Is there a strategic plan for recruitment of personnel per water treatment plant available? | | **1** | |  |
|  | Is there a strategic plan for operational monitoring and verification of drinking water system? | | **1** | |  |
|  | Is there a strategic plan for sharing and clarifying roles and responsibilities among all personnel involved in the production of safe drinking water quality? | | **1** | |  |
|  | Does a strategic plan for training and development of water plant personnel, and for community involvement and awareness exist? | | **1** | |  |
|  | Is there any funding strategic plan for implementation of drinking water quality management programmes? | | **1** | |  |
| **Assessment of tact plans** | Does a tactical plan for handling emergencies including communication of such emergencies to communities exist? | | **1** | |  |
|  | Is the overall periodic overseeing of water care function performed? | | **1** | |  |
|  | Is there maintenance and asset management plans? | | **1** | |  |
| **Assessment of operational plans** | Is the attendance of process controllers (and in particular shift workers) at the treatment plant monitored? Is there a clock–in-system for the attendance? | | **1** | |  |
|  | Is the superintendent available to operators on a day-to-day basis? | | **1** | |  |
|  | Is there a substitute system plan put in place when one of the operators is ill or away attending a course? | | **1** | |  |
|  | Are the supervisors and process controllers aware of their job requirements? Are job descriptions available? | | **1** | |  |
| **Maximum possible score for *Management Issues*** | | | **14** | | |
| **Total score attained for *Management Issues*** | | | **14** | | |
| **Weight for *Management Issues*** | | | **0.1** | | |
| **Total weighted score for *Management Issues*** | | | **0.1** | | |

| **NON-TECHNICAL COMPLIANCE (MANAGEMENT) SCORING: Table 8.3** | | | |
| --- | --- | --- | --- |
| **Criterion** | | **Yes =1** | **No = 0** |
| **2. NON-TECHNICAL ASSESSMENT** | | | |
| **2B: Management Practices** | | | |
|  | Do the WSPs use existing local (Emanti/DWAF) or international management practices? Are they implementing them in their water treatment plants? | **1** |  |
|  | Are the WPSs able to understand the existing local or international practices and implementing them correctly? | **1** |  |
|  | When local or international practices are not available, are the WSPs able to draw their own criteria? | **1** |  |
| **Maximum possible score for *Management Practices*** | | **3** | |
| **Total score attained for *Management Practices*** | | **3** | |
| **Weight for *Management Practices*** | | **0.2** | |
| **Total weighted score for *Management Practices*** | | **0.2** | |

| **NON-TECHNICAL COMPLIANCE (MANAGEMENT) SCORING: Table 8.4** | | | | | |
| --- | --- | --- | --- | --- | --- |
| **Criterion** | | **Yes =1** | | **No = 0** | |
| **2. NON-TECHNICAL ASSESSMENT** | | | | | |
| **2C: Human Resources** | | | | | |
| **Personnel at the plant** | Is the number of personnel related to the size of the plant? | |  | | **0** |
|  | Are the needs of process controllers/supervisors and shift workers per plants adequate? | |  | | **0** |
|  | Are there specific guidelines/rules for shift workers and their deployment? | | **1** | |  |
|  | How many operators are there in total? How many shift workers? | | **1** | |  |
| **Qualifications of Personnel** | How many operators with qualifications in the DWAF categories? | | **1** | |  |
|  | Are personnel being correctly applied according to their qualifications? | | **1** | |  |
|  | Are there guidelines or formal rules for upgrading of qualifications? | | **1** | |  |
| **Training Needs** | Are there in service training programmes and policies? What is the in-service training policy of the WSA? | | **1** | |  |
|  | Do process controllers / supervisors / managers know what training opportunities and courses are available? | | **1** | |  |
|  | Are they sent on courses regularly? | | **1** | |  |
|  | Are there specific criteria or formal rules to be qualified for training? | | **1** | |  |
|  | Are there specific promotion requirement protocols in place? | | **1** | |  |
|  | Are there career advancement opportunities? | | **1** | |  |
| **Plant class** | Is the plant classified by DWAF? | | **1** | |  |
|  | Has it been done according to the new classification system? | | **1** | |  |
| **Working Conditions** | Are the responsibilities of process controllers, supervisors and plant managers in line with their salary? | | **1** | |  |
|  | What system is used for overtime management and payment? | | **1** | |  |
|  | What is the WSA policy on conflict management (internal and external)? | | **1** | |  |
|  | Is any medical assistance available for on the job injuries? | | **1** | |  |
|  | Can all workers participate in medical schemes? | | **1** | |  |
|  | Are the retirement/pension-aid benefits, the housing and travel allowance benefits available? | | **1** | |  |
|  | Are the leave benefits (vacation /study/ sick), and the bonus scheme available? | | **1** | |  |
|  | Are there any other incentives? | | **1** | |  |
|  | Are plant personnel satisfied with their working environment? | | **1** | |  |
|  | Are the relationships between supervisors and their subordinates good or poor? | | **1** | |  |
|  | Is there any performance indicators? | | **1** | |  |
| **Maximum possible score for *Human Resources*** | | | **27** | | |
| **Total score attained for *Human Resources*** | | | **25** | | |
| **Weight for *Human Resources*** | | | **0.2** | | |
| **Total weighted score for *Human Resources*** | | | **0.185** | | |

| **NON-TECHNICAL COMPLIANCE (MANAGEMENT) SCORING: Table 8.5** | | | |
| --- | --- | --- | --- |
| **Criterion** | | **Yes =1** | **No = 0** |
| **2. NON-TECHNICAL ASSESSMENT** | | | |
| **2D: Financial Systems** | | | |
| **Financial Systems Sharing in Place** | Which financial systems are in place? | **1** |  |
|  | Are there sufficient funds for operation, maintenance and overtime? | **1** |  |
|  | Do the size of the plants (small versus large) influence the allocation of funds? | **1** |  |
|  | Does the WSA’s budgeting system work in relation to emergency funds, scheduled upgrading/extensions, unscheduled rehabilitation/upgrading? | **1** |  |
| **Information Sharing in Place** | Are there communication channels and transparency between the financial section and the water care section? | **1** |  |
|  | Are regular meetings held? Who attends / chairs these meetings? | **1** |  |
|  | Are decisions taken at consensus / majority? | **1** |  |
|  | Are reports freely available? | **1** |  |
| **Procurement System** | Is the procurement system transparent? | **1** |  |
|  | Is the procurement system open to all? | **1** |  |
|  | Is the water sector involved in the adjudication of tenders? | **1** |  |
| **Maximum possible score for *Financial Systems*** | | **11** | |
| **Total score attained for *Financial Systems*** | | **11** | |
| **Weight for *Financial Systems*** | | **0.1** | |
| **Total weighted score for *Financial Systems*** | | **0.1** | |

| **NON-TECHNICAL COMPLIANCE (MANAGEMENT) SCORING: Table 8.6** | | | |
| --- | --- | --- | --- |
| **Criterion** | | **Yes =1** | **No = 0** |
| **2. NON-TECHNICAL ASSESSMENT** | | | |
| **2E: Communication Systems** | | | |
| **Internal** | Are there sufficient internal communication channels in place, including the needs and shortcomings? | **1** |  |
|  | Are these communication channels effective? | **1** |  |
|  | Is the communication between supervisors and process controllers good or poor? | **1** |  |
|  | Is there any interaction between the maintenance team and the rest of personnel in the water treatment plant? | **1** |  |
|  | Is the communication between management and lower level process controllers good or poor? | **1** |  |
| **External** | Are there sufficient communication channels with other Municipalities and/or Departments? | **1** |  |
|  | Are there sufficient communication channels with consumers? | **1** |  |
|  | Are these external communication channels effective, good or poor? | **1** |  |
| **Maximum possible score for *Communication Systems*** | | **8** | |
| **Total score attained for *Communication Systems*** | | **8** | |
| **Weight for *Communication Systems*** | | **0.2** | |
| **Total weighted score for *Communication Systems*** | | **0.2** | |

| **NON-TECHNICAL COMPLIANCE (MANAGEMENT) SCORING: Table 8.7** | | | |
| --- | --- | --- | --- |
| **Criterion** | | **Yes =1** | **No = 0** |
| **2. NON-TECHNICAL ASSESSMENT** | | | |
| **2F: Audit of safety, health and environmental quality** | | | |
| **Safety Matters** | Are there water and safety management procedures for normal operation, incidents and emergency situations? | **1** |  |
|  | Is there any safety plan at water treatment plants including the safety of process controllers on duties? | **1** |  |
|  | Are there emergency plans in place for chlorine leaks? | **1** |  |
|  | Are there hazardous chemicals and ablution facilities, appropriate fire extinguishers location? | **1** |  |
|  | Are safety meetings held regularly and who attends such meetings? | **1** |  |
| **Health Aspects** | Are there representatives for health and safety of consumers? If yes, are they effective? | **1** |  |
|  | Are there control measures that identify risks and ensure that health based- targets are met in terms of providing safe drinking water to all consumers? | **1** |  |
|  | Are there emergency measures in place for water quality health impact? | **1** |  |
| **Environmental Quality** | Are process controllers satisfied with their working environment? | **1** |  |
|  | Is the working environment appropriate for improving or accelerating the service delivery? | **1** |  |
|  | Is there any strategic plan to improve the quality of the working environment? |  | **0** |
| **Maximum possible score for *SHEQ*** | | **11** | |
| **Total score attained for *SHEQ*** | | **10** | |
| **Weight for *SHEQ*** | | **0.15** | |
| **Total weighted score for *SHEQ*** | | **0.136** | |

| **NON-TECHNICAL COMPLIANCE (MANAGEMENT) SCORING: Table 8.8** | | | |
| --- | --- | --- | --- |
| **Criterion** | | **Yes =1** | **No = 0** |
| **2. NON-TECHNICAL ASSESSMENT** | | | |
| **2G: Community involvement and awareness** | | | |
| **Community Involvement** | Are there protocols involved communities in decision-making? | **1** |  |
|  | Is there an effective reporting system for communities to assist in more rapid response to any water quality incident? | **1** |  |
|  | Is there a Consumer Service to which non-compliance can be reported? | **1** |  |
| **Community Awareness** | Is there a mechanism system to receive and actively address community complaints in a timely fashion? | **1** |  |
|  | Are there procedures for promptly advising of any significant incidents within the drinking water supply including notification of the public health authority? | **1** |  |
|  | Is there any water quality information system available to consumers through annual reports and in the internet? | **1** |  |
| **Maximum possible score for *Community Involvement*** | | **6** | |
| **Total score attained for *Community Involvement*** | | **6** | |
| **Weight for *Community Involvement*** | | **0.05** | |
| **Total weighted score for *Community Involvement*** | | **0.05** | |

| **TABLE 8.9**  **NON-TECHNICAL (MANAGEMENT) COMPLIANCE SCORING:**  **Total Weighted Scoring for Vondo treatment plant 97.1 %** | |
| --- | --- |
| **Compliance Criterion** | **Weighted Score** |
| **2. NON-TECHNICAL ASSESSMENT** | |
| **2A: Management Issues** | **0.1** |
| **2B: Management Practices** | **0.2** |
| **2C: Human Resources** | **0.185** |
| **2D: Financial Systems** | **0.1** |
| **2E: Communication Systems** | **0.2** |
| **2F: Safety, Health and Environmental Quality** | **0.136** |
| **2G: Community Involvement and Awareness** | **0.05** |
| **TOTAL WEIGHTED SCORE** | **97.1** |

| **TABLE 8.10**  **NON-TECHNICAL (MANAGEMENT) COMPLIANCE RATING:** | |
| --- | --- |
| **Total Weighted Score** | **Rating Description** |
| **0 – 50** | ***Class 3 Compliance*:**  Total non-compliance; serious and immediate intervention required (TAC) |
| **50 – 90** | ***Class 2 Compliance:***  Serious challenges requiring attention and improvement |
| **90 – 100** | ***Class 1 Compliance:***  Acceptable compliance |

**mutshedzi Plant**

**12 May 2011**

| **TABLE 9.1**  **NON-TECHNICAL (MANAGEMENT) COMPLIANCE SCORING** | |
| --- | --- |
| **Criterion** | **Weight** |
| **2. NON-TECHNICAL ASSESSMENT** | |
| **2A: Management Issues** | **0.1** |
| **2B: Management Practices** | **0.2** |
| **2C: Human Resources** | **0.2** |
| **2D: Financial Systems** | **0.1** |
| **2E: Communication Systems** | **0.2** |
| **2F: Safety, Health and Environmental Quality** | **0.15** |
| **2G: Community Involvement and Awareness** | **0.05** |
| **TOTAL** | **1.0** |

| **NON-TECHNICAL COMPLIANCE (MANAGEMENT) SCORING: Table 9.2** | | | | | |
| --- | --- | --- | --- | --- | --- |
| **Criterion** | | **Yes =1** | | **No = 0** | |
| **2. NON-TECHNICAL ASSESSMENT** | | | | | |
| **2A: Management Issues** | | | | | |
| **Assessment of strategic plans** | Does a strategic plan for the assessment of drinking water supply systems exist? | | **1** | |  |
|  | Are there preventive strategic plans for drinking water quality management? | | **1** | |  |
|  | Is there a strategic plan for recruitment of personnel per water treatment plant available? | | **1** | |  |
|  | Is there a strategic plan for operational monitoring and verification of drinking water system? | | **1** | |  |
|  | Is there a strategic plan for sharing and clarifying roles and responsibilities among all personnel involved in the production of safe drinking water quality? | | **1** | |  |
|  | Does a strategic plan for training and development of water plant personnel, and for community involvement and awareness exist? | | **1** | |  |
|  | Is there any funding strategic plan for implementation of drinking water quality management programmes? | | **1** | |  |
| **Assm of tact plans** | Does a tactical plan for handling emergencies including communication of such emergencies to communities exist? | | **1** | |  |
|  | Is the overall periodic overseeing of water care function performed? | | **1** | |  |
|  | Is there maintenance and asset management plans? | | **1** | |  |
| **Assessment of operational plans** | Is the attendance of process controllers (and in particular shift workers) at the treatment plant monitored? Is there a clock–in-system for the attendance? | | **1** | |  |
|  | Is the superintendent available to operators on a day-to-day basis? | | **1** | |  |
|  | Is there a substitute system plan put in place when one of the operators is ill or away attending a course? | | **1** | |  |
|  | Are the supervisors and process controllers aware of their job requirements? Are job descriptions available? | | **1** | |  |
| **Maximum possible score for *Management Issues*** | | | **14** | | |
| **Total score attained for *Management Issues*** | | | **14** | | |
| **Weight for *Management Issues*** | | | **0.1** | | |
| **Total weighted score for *Management Issues*** | | | **0.1** | | |

| **NON-TECHNICAL COMPLIANCE (MANAGEMENT) SCORING: Table 9.3** | | | |
| --- | --- | --- | --- |
| **Criterion** | | **Yes =1** | **No = 0** |
| **2. NON-TECHNICAL ASSESSMENT** | | | |
| **2B: Management Practices** | | | |
|  | Do the WSPs use existing local (Emanti/DWAF) or international management practices? Are they implementing them in their water treatment plants? | **1** |  |
|  | Are the WPSs able to understand the existing local or international practices and implementing them correctly? | **1** |  |
|  | When local or international practices are not available, are the WSPs able to draw their own criteria? | **1** |  |
| **Maximum possible score for *Management Practices*** | | **3** | |
| **Total score attained for *Management Practices*** | | **3** | |
| **Weight for *Management Practices*** | | **0.2** | |
| **Total weighted score for *Management Practices*** | | **0.2** | |

| **NON-TECHNICAL COMPLIANCE (MANAGEMENT) SCORING: Table 9.4** | | | | | |
| --- | --- | --- | --- | --- | --- |
| **Criterion** | | **Yes =1** | | **No = 0** | |
| **2. NON-TECHNICAL ASSESSMENT** | | | | | |
| **2C: Human Resources** | | | | | |
| **Personnel at the plant** | Is the number of personnel related to the size of the plant? | |  | | **0** |
|  | Are the needs of process controllers/supervisors and shift workers per plants adequate? | |  | | **0** |
|  | Are there specific guidelines/rules for shift workers and their deployment? | | **1** | |  |
|  | How many operators are there in total? How many shift workers? | | **1** | |  |
| **Qualifications of Personnel** | How many operators with qualifications in the DWAF categories? | | **1** | |  |
|  | Are personnel being correctly applied according to their qualifications? | | **1** | |  |
|  | Are there guidelines or formal rules for upgrading of qualifications? | | **1** | |  |
| **Training Needs** | Are there in service training programmes and policies? What is the in-service training policy of the WSA? | | **1** | |  |
|  | Do process controllers / supervisors / managers know what training opportunities and courses are available? | | **1** | |  |
|  | Are they sent on courses regularly? | | **1** | |  |
|  | Are there specific criteria or formal rules to be qualified for training? | | **1** | |  |
|  | Are there specific promotion requirement protocols in place? | | **1** | |  |
|  | Are there career advancement opportunities? | | **1** | |  |
| **Plant class** | Is the plant classified by DWAF? | | **1** | |  |
|  | Has it been done according to the new classification system? | | **1** | |  |
| **Working Conditions** | Are the responsibilities of process controllers, supervisors and plant managers in line with their salary? | | **1** | |  |
|  | What system is used for overtime management and payment? | | **1** | |  |
|  | What is the WSA policy on conflict management (internal and external)? | | **1** | |  |
|  | Is any medical assistance available for on the job injuries? | | **1** | |  |
|  | Can all workers participate in medical schemes? | | **1** | |  |
|  | Are the retirement/pension-aid benefits, the housing and travel allowance benefits available? | | **1** | |  |
|  | Are the leave benefits (vacation /study/ sick), and the bonus scheme available? | | **1** | |  |
|  | Are there any other incentives? | | **1** | |  |
|  | Are plant personnel satisfied with their working environment? | | **1** | |  |
|  | Are the relationships between supervisors and their subordinates good or poor? | | **1** | |  |
|  | Is there any performance indicators? | | **1** | |  |
| **Maximum possible score for *Human Resources*** | | | **27** | | |
| **Total score attained for *Human Resources*** | | | **25** | | |
| **Weight for *Human Resources*** | | | **0.2** | | |
| **Total weighted score for *Human Resources*** | | | **0.185** | | |

| **NON-TECHNICAL COMPLIANCE (MANAGEMENT) SCORING: Table 9.5** | | | |
| --- | --- | --- | --- |
| **Criterion** | | **Yes =1** | **No = 0** |
| **2. NON-TECHNICAL ASSESSMENT** | | | |
| **2D: Financial Systems** | | | |
| **Financial Systems Sharing in Place** | Which financial systems are in place? | **1** |  |
|  | Are there sufficient funds for operation, maintenance and overtime? | **1** |  |
|  | Do the size of the plants (small versus large) influence the allocation of funds? | **1** |  |
|  | Does the WSA’s budgeting system work in relation to emergency funds, scheduled upgrading/extensions, unscheduled rehabilitation/upgrading? | **1** |  |
| **Information Sharing in Place** | Are there communication channels and transparency between the financial section and the water care section? | **1** |  |
|  | Are regular meetings held? Who attends / chairs these meetings? | **1** |  |
|  | Are decisions taken at consensus / majority? | **1** |  |
|  | Are reports freely available? | **1** |  |
| **Procurement System** | Is the procurement system transparent? | **1** |  |
|  | Is the procurement system open to all? | **1** |  |
|  | Is the water sector involved in the adjudication of tenders? | **1** |  |
| **Maximum possible score for *Financial Systems*** | | **11** | |
| **Total score attained for *Financial Systems*** | | **11** | |
| **Weight for *Financial Systems*** | | **0.1** | |
| **Total weighted score for *Financial Systems*** | | **0.1** | |

| **NON-TECHNICAL COMPLIANCE (MANAGEMENT) SCORING: Table 9.6** | | | |
| --- | --- | --- | --- |
| **Criterion** | | **Yes =1** | **No = 0** |
| **2. NON-TECHNICAL ASSESSMENT** | | | |
| **2E: Communication Systems** | | | |
| **Internal** | Are there sufficient internal communication channels in place, including the needs and shortcomings? | **1** |  |
|  | Are these communication channels effective? | **1** |  |
|  | Is the communication between supervisors and process controllers good or poor? | **1** |  |
|  | Is there any interaction between the maintenance team and the rest of personnel in the water treatment plant? | **1** |  |
|  | Is the communication between management and lower level process controllers good or poor? | **1** |  |
| **External** | Are there sufficient communication channels with other Municipalities and/or Departments? | **1** |  |
|  | Are there sufficient communication channels with consumers? | **1** |  |
|  | Are these external communication channels effective, good or poor? | **1** |  |
| **Maximum possible score for *Communication Systems*** | | **8** | |
| **Total score attained for *Communication Systems*** | | **8** | |
| **Weight for *Communication Systems*** | | **0.2** | |
| **Total weighted score for *Communication Systems*** | | **0.2** | |

| **NON-TECHNICAL COMPLIANCE (MANAGEMENT) SCORING: Table 9.7** | | | |
| --- | --- | --- | --- |
| **Criterion** | | **Yes =1** | **No = 0** |
| **2. NON-TECHNICAL ASSESSMENT** | | | |
| **2F: Audit of safety, health and environmental quality** | | | |
| **Safety Matters** | Are there water and safety management procedures for normal operation, incidents and emergency situations? | **1** |  |
|  | Is there any safety plan at water treatment plants including the safety of process controllers on duties? | **1** |  |
|  | Are there emergency plans in place for chlorine leaks? | **1** |  |
|  | Are there hazardous chemicals and ablution facilities, appropriate fire extinguishers location? | **1** |  |
|  | Are safety meetings held regularly and who attends such meetings? | **1** |  |
| **Health Aspects** | Are there representatives for health and safety of consumers? If yes, are they effective? | **1** |  |
|  | Are there control measures that identify risks and ensure that health based- targets are met in terms of providing safe drinking water to all consumers? | **1** |  |
|  | Are there emergency measures in place for water quality health impact? | **1** |  |
| **Environmental Quality** | Are process controllers satisfied with their working environment? | **1** |  |
|  | Is the working environment appropriate for improving or accelerating the service delivery? | **1** |  |
|  | Is there any strategic plan to improve the quality of the working environment? | **1** |  |
| **Maximum possible score for *SHEQ*** | | **11** | |
| **Total score attained for *SHEQ*** | | **11** | |
| **Weight for *SHEQ*** | | **0.15** | |
| **Total weighted score for *SHEQ*** | | **0.15** | |

| **NON-TECHNICAL COMPLIANCE (MANAGEMENT) SCORING: Table 9.8** | | | |
| --- | --- | --- | --- |
| **Criterion** | | **Yes =1** | **No = 0** |
| **2. NON-TECHNICAL ASSESSMENT** | | | |
| **2G: Community involvement and awareness** | | | |
| **Community Involvement** | Are there protocols involved communities in decision-making? |  | **0** |
|  | Is there an effective reporting system for communities to assist in more rapid response to any water quality incident? | **1** |  |
|  | Is there a Consumer Service to which non-compliance can be reported? | **1** |  |
| **Community Awareness** | Is there a mechanism system to receive and actively address community complaints in a timely fashion? | **1** |  |
|  | Are there procedures for promptly advising of any significant incidents within the drinking water supply including notification of the public health authority? | **1** |  |
|  | Is there any water quality information system available to consumers through annual reports and in the internet? | **1** |  |
| **Maximum possible score for *Community Involvement*** | | **6** | |
| **Total score attained for *Community Involvement*** | | **5** | |
| **Weight for *Community Involvement*** | | **0.05** | |
| **Total weighted score for *Community Involvement*** | | **0.042** | |

| **TABLE 9.9**  **NON-TECHNICAL (MANAGEMENT) COMPLIANCE SCORING:**  **Total Weighted Scoring for Mutshedzi package plant 97.7 %** | |
| --- | --- |
| **Compliance Criterion** | **Weighted Score** |
| **2. NON-TECHNICAL ASSESSMENT** | |
| **2A: Management Issues** | **0.1** |
| **2B: Management Practices** | **0.2** |
| **2C: Human Resources** | **0.185** |
| **2D: Financial Systems** | **0.1** |
| **2E: Communication Systems** | **0.2** |
| **2F: Safety, Health and Environmental Quality** | **0.15** |
| **2G: Community Involvement and Awareness** | **0.042** |
| **TOTAL WEIGHTED SCORE** | **97.7** |

| **TABLE 9.10**  **NON-TECHNICAL (MANAGEMENT) COMPLIANCE RATING:** | |
| --- | --- |
| **Total Weighted Score** | **Rating Description** |
| **0 – 50** | ***Class 3 Compliance*:**  Total non-compliance; serious and immediate intervention required (TAC) |
| **50 – 90** | ***Class 2 Compliance:***  Serious challenges requiring attention and improvement |
| **90 – 100** | ***Class 1 Compliance:***  Acceptable compliance |

**MUTALE REGIONAL WATER TREATMENT Plant**

**11 May 2011**

| **TABLE 10.1**  **NON-TECHNICAL (MANAGEMENT) COMPLIANCE SCORING** | |
| --- | --- |
| **Criterion** | **Weight** |
| **2. NON-TECHNICAL ASSESSMENT** | |
| **2A: Management Issues** | **0.1** |
| **2B: Management Practices** | **0.2** |
| **2C: Human Resources** | **0.2** |
| **2D: Financial Systems** | **0.1** |
| **2E: Communication Systems** | **0.2** |
| **2F: Safety, Health and Environmental Quality** | **0.15** |
| **2G: Community Involvement and Awareness** | **0.05** |
| **TOTAL** | **1.0** |

| **NON-TECHNICAL COMPLIANCE (MANAGEMENT) SCORING: Table 10.2** | | | | | |
| --- | --- | --- | --- | --- | --- |
| **Criterion** | | **Yes =1** | | **No = 0** | |
| **2. NON-TECHNICAL ASSESSMENT** | | | | | |
| **2A: Management Issues** | | | | | |
| **Assessment of strategic plans** | Does a strategic plan for the assessment of drinking water supply systems exist? | | **1** | |  |
|  | Are there preventive strategic plans for drinking water quality management? | | **1** | |  |
|  | Is there a strategic plan for recruitment of personnel per water treatment plant available? | | **1** | |  |
|  | Is there a strategic plan for operational monitoring and verification of drinking water system? | | **1** | |  |
|  | Is there a strategic plan for sharing and clarifying roles and responsibilities among all personnel involved in the production of safe drinking water quality? | | **1** | |  |
|  | Does a strategic plan for training and development of water plant personnel, and for community involvement and awareness exist? | | **1** | |  |
|  | Is there any funding strategic plan for implementation of drinking water quality management programmes? | | **1** | |  |
| **Assm of tact plans** | Does a tactical plan for handling emergencies including communication of such emergencies to communities exist? | | **1** | |  |
|  | Is the overall periodic overseeing of water care function performed? | | **1** | |  |
|  | Is there maintenance and asset management plans? | | **1** | |  |
| **Assessment of operational plans** | Is the attendance of process controllers (and in particular shift workers) at the treatment plant monitored? Is there a clock–in-system for the attendance? | | **1** | |  |
|  | Is the superintendent available to operators on a day-to-day basis? | | **1** | |  |
|  | Is there a substitute system plan put in place when one of the operators is ill or away attending a course? | | **1** | |  |
|  | Are the supervisors and process controllers aware of their job requirements? Are job descriptions available? | | **1** | |  |
| **Maximum possible score for *Management Issues*** | | | **14** | | |
| **Total score attained for *Management Issues*** | | | **14** | | |
| **Weight for *Management Issues*** | | | **0.1** | | |
| **Total weighted score for *Management Issues*** | | | **0.1** | | |

| **NON-TECHNICAL COMPLIANCE (MANAGEMENT) SCORING: Table 10.3** | | | |
| --- | --- | --- | --- |
| **Criterion** | | **Yes =1** | **No = 0** |
| **2. NON-TECHNICAL ASSESSMENT** | | | |
| **2B: Management Practices** | | | |
|  | Do the WSPs use existing local (Emanti/DWAF) or international management practices? Are they implementing them in their water treatment plants? | **1** |  |
|  | Are the WPSs able to understand the existing local or international practices and implementing them correctly? | **1** |  |
|  | When local or international practices are not available, are the WSPs able to draw their own criteria? | **1** |  |
| **Maximum possible score for *Management Practices*** | | **3** | |
| **Total score attained for *Management Practices*** | | **3** | |
| **Weight for *Management Practices*** | | **0.2** | |
| **Total weighted score for *Management Practices*** | | **0.2** | |

| **NON-TECHNICAL COMPLIANCE (MANAGEMENT) SCORING: Table 10.4** | | | | | |
| --- | --- | --- | --- | --- | --- |
| **Criterion** | | **Yes =1** | | **No = 0** | |
| **2. NON-TECHNICAL ASSESSMENT** | | | | | |
| **2C: Human Resources** | | | | | |
| **Personnel at the plant** | Is the number of personnel related to the size of the plant? | |  | | **0** |
|  | Are the needs of process controllers/supervisors and shift workers per plants adequate? | |  | | **0** |
|  | Are there specific guidelines/rules for shift workers and their deployment? | | **1** | |  |
|  | How many operators are there in total? How many shift workers? | | **1** | |  |
| **Qualifications of Personnel** | How many operators with qualifications in the DWAF categories? | | **1** | |  |
|  | Are personnel being correctly applied according to their qualifications? | | **1** | |  |
|  | Are there guidelines or formal rules for upgrading of qualifications? | | **1** | |  |
| **Training Needs** | Are there in service training programmes and policies? What is the in-service training policy of the WSA? | | **1** | |  |
|  | Do process controllers / supervisors / managers know what training opportunities and courses are available? | | **1** | |  |
|  | Are they sent on courses regularly? | | **1** | |  |
|  | Are there specific criteria or formal rules to be qualified for training? | | **1** | |  |
|  | Are there specific promotion requirement protocols in place? | | **1** | |  |
|  | Are there career advancement opportunities? | | **1** | |  |
| **Plant class** | Is the plant classified by DWAF? | | **1** | |  |
|  | Has it been done according to the new classification system? | | **1** | |  |
| **Working Conditions** | Are the responsibilities of process controllers, supervisors and plant managers in line with their salary? | | **1** | |  |
|  | What system is used for overtime management and payment? | | **1** | |  |
|  | What is the WSA policy on conflict management (internal and external)? | | **1** | |  |
|  | Is any medical assistance available for on the job injuries? | | **1** | |  |
|  | Can all workers participate in medical schemes? | | **1** | |  |
|  | Are the retirement/pension-aid benefits, the housing and travel allowance benefits available? | | **1** | |  |
|  | Are the leave benefits (vacation /study/ sick), and the bonus scheme available? | | **1** | |  |
|  | Are there any other incentives? | | **1** | |  |
|  | Are plant personnel satisfied with their working environment? | | **1** | |  |
|  | Are the relationships between supervisors and their subordinates good or poor? | | **1** | |  |
|  | Is there any performance indicators? | | **1** | |  |
| **Maximum possible score for *Human Resources*** | | | **27** | | |
| **Total score attained for *Human Resources*** | | | **25** | | |
| **Weight for *Human Resources*** | | | **0.2** | | |
| **Total weighted score for *Human Resources*** | | | **0.185** | | |

| **NON-TECHNICAL COMPLIANCE (MANAGEMENT) SCORING: Table 10.5** | | | |
| --- | --- | --- | --- |
| **Criterion** | | **Yes =1** | **No = 0** |
| **2. NON-TECHNICAL ASSESSMENT** | | | |
| **2D: Financial Systems** | | | |
| **Financial Systems Sharing in Place** | Which financial systems are in place? | **1** |  |
|  | Are there sufficient funds for operation, maintenance and overtime? | **1** |  |
|  | Do the size of the plants (small versus large) influence the allocation of funds? | **1** |  |
|  | Does the WSA’s budgeting system work in relation to emergency funds, scheduled upgrading/extensions, unscheduled rehabilitation/upgrading? | **1** |  |
| **Information Sharing in Place** | Are there communication channels and transparency between the financial section and the water care section? | **1** |  |
|  | Are regular meetings held? Who attends / chairs these meetings? | **1** |  |
|  | Are decisions taken at consensus / majority? | **1** |  |
|  | Are reports freely available? | **1** |  |
| **Procurement System** | Is the procurement system transparent? | **1** |  |
|  | Is the procurement system open to all? | **1** |  |
|  | Is the water sector involved in the adjudication of tenders? | **1** |  |
| **Maximum possible score for *Financial Systems*** | | **11** | |
| **Total score attained for *Financial Systems*** | | **11** | |
| **Weight for *Financial Systems*** | | **0.1** | |
| **Total weighted score for *Financial Systems*** | | **0.1** | |

| **NON-TECHNICAL COMPLIANCE (MANAGEMENT) SCORING: Table 10.6** | | | |
| --- | --- | --- | --- |
| **Criterion** | | **Yes =1** | **No = 0** |
| **2. NON-TECHNICAL ASSESSMENT** | | | |
| **2E: Communication Systems** | | | |
| **Internal** | Are there sufficient internal communication channels in place, including the needs and shortcomings? | **1** |  |
|  | Are these communication channels effective? | **1** |  |
|  | Is the communication between supervisors and process controllers good or poor? | **1** |  |
|  | Is there any interaction between the maintenance team and the rest of personnel in the water treatment plant? | **1** |  |
|  | Is the communication between management and lower level process controllers good or poor? | **1** |  |
| **External** | Are there sufficient communication channels with other Municipalities and/or Departments? | **1** |  |
|  | Are there sufficient communication channels with consumers? | **1** |  |
|  | Are these external communication channels effective, good or poor? | **1** |  |
| **Maximum possible score for *Communication Systems*** | | **8** | |
| **Total score attained for *Communication Systems*** | | **8** | |
| **Weight for *Communication Systems*** | | **0.2** | |
| **Total weighted score for *Communication Systems*** | | **0.2** | |

| **NON-TECHNICAL COMPLIANCE (MANAGEMENT) SCORING: Table 10.7** | | | |
| --- | --- | --- | --- |
| **Criterion** | | **Yes =1** | **No = 0** |
| **2. NON-TECHNICAL ASSESSMENT** | | | |
| **2F: Audit of safety, health and environmental quality** | | | |
| **Safety Matters** | Are there water and safety management procedures for normal operation, incidents and emergency situations? | **1** |  |
|  | Is there any safety plan at water treatment plants including the safety of process controllers on duties? | **1** |  |
|  | Are there emergency plans in place for chlorine leaks? | **1** |  |
|  | Are there hazardous chemicals and ablution facilities, appropriate fire extinguishers location? | **1** |  |
|  | Are safety meetings held regularly and who attends such meetings? | **1** |  |
| **Health Aspects** | Are there representatives for health and safety of consumers? If yes, are they effective? | **1** |  |
|  | Are there control measures that identify risks and ensure that health based- targets are met in terms of providing safe drinking water to all consumers? | **1** |  |
|  | Are there emergency measures in place for water quality health impact? | **1** |  |
| **Environmental Quality** | Are process controllers satisfied with their working environment? | **1** |  |
|  | Is the working environment appropriate for improving or accelerating the service delivery? | **1** |  |
|  | Is there any strategic plan to improve the quality of the working environment? | **1** |  |
| **Maximum possible score for *SHEQ*** | | **11** | |
| **Total score attained for *SHEQ*** | | **11** | |
| **Weight for *SHEQ*** | | **0.15** | |
| **Total weighted score for *SHEQ*** | | **0.15** | |

| **NON-TECHNICAL COMPLIANCE (MANAGEMENT) SCORING: Table 10.8** | | | |
| --- | --- | --- | --- |
| **Criterion** | | **Yes =1** | **No = 0** |
| **2. NON-TECHNICAL ASSESSMENT** | | | |
| **2G: Community involvement and awareness** | | | |
| **Community Involvement** | Are there protocols involved communities in decision-making? |  | **0** |
|  | Is there an effective reporting system for communities to assist in more rapid response to any water quality incident? | **1** |  |
|  | Is there a Consumer Service to which non-compliance can be reported? | **1** |  |
| **Community Awareness** | Is there a mechanism system to receive and actively address community complaints in a timely fashion? | **1** |  |
|  | Are there procedures for promptly advising of any significant incidents within the drinking water supply including notification of the public health authority? | **1** |  |
|  | Is there any water quality information system available to consumers through annual reports and in the internet? | **1** |  |
| **Maximum possible score for *Community Involvement*** | | **6** | |
| **Total score attained for *Community Involvement*** | | **5** | |
| **Weight for *Community Involvement*** | | **0.05** | |
| **Total weighted score for *Community Involvement*** | | **0.042** | |

| **TABLE 10.9**  **NON-TECHNICAL (MANAGEMENT) COMPLIANCE SCORING:**  **Total Weighted Scoring for Mutale Regional Water Plant 97.7 %** | |
| --- | --- |
| **Compliance Criterion** | **Weighted Score** |
| **2. NON-TECHNICAL ASSESSMENT** | |
| **2A: Management Issues** | **0.1** |
| **2B: Management Practices** | **0.2** |
| **2C: Human Resources** | **0.185** |
| **2D: Financial Systems** | **0.1** |
| **2E: Communication Systems** | **0.2** |
| **2F: Safety, Health and Environmental Quality** | **0.15** |
| **2G: Community Involvement and Awareness** | **0.042** |
| **TOTAL WEIGHTED SCORE** | **97.7** |

| **TABLE 10.10**  **NON-TECHNICAL (MANAGEMENT) COMPLIANCE RATING:** | |
| --- | --- |
| **Total Weighted Score** | **Rating Description** |
| **0 – 50** | ***Class 3 Compliance*:**  Total non-compliance; serious and immediate intervention required (TAC) |
| **50 – 90** | ***Class 2 Compliance:***  Serious challenges requiring attention and improvement |
| **90 – 100** | ***Class 1 Compliance:***  Acceptable compliance |

**TSHEDZA Package Plant**

**12 May 2011**

| **TABLE 11.1**  **NON-TECHNICAL (MANAGEMENT) COMPLIANCE SCORING** | |
| --- | --- |
| **Criterion** | **Weight** |
| **2. NON-TECHNICAL ASSESSMENT** | |
| **2A: Management Issues** | **0.1** |
| **2B: Management Practices** | **0.2** |
| **2C: Human Resources** | **0.2** |
| **2D: Financial Systems** | **0.1** |
| **2E: Communication Systems** | **0.2** |
| **2F: Safety, Health and Environmental Quality** | **0.15** |
| **2G: Community Involvement and Awareness** | **0.05** |
| **TOTAL** | **1.0** |

| **NON-TECHNICAL COMPLIANCE (MANAGEMENT) SCORING: Table 11.2** | | | | | |
| --- | --- | --- | --- | --- | --- |
| **Criterion** | | **Yes =1** | | **No = 0** | |
| **2. NON-TECHNICAL ASSESSMENT** | | | | | |
| **2A: Management Issues** | | | | | |
| **Assessment of strategic plans** | Does a strategic plan for the assessment of drinking water supply systems exist? | | **1** | |  |
|  | Are there preventive strategic plans for drinking water quality management? | | **1** | |  |
|  | Is there a strategic plan for recruitment of personnel per water treatment plant available? | | **1** | |  |
|  | Is there a strategic plan for operational monitoring and verification of drinking water system? | | **1** | |  |
|  | Is there a strategic plan for sharing and clarifying roles and responsibilities among all personnel involved in the production of safe drinking water quality? | | **1** | |  |
|  | Does a strategic plan for training and development of water plant personnel, and for community involvement and awareness exist? | | **1** | |  |
|  | Is there any funding strategic plan for implementation of drinking water quality management programmes? | | **1** | |  |
| **Assm of tact plans** | Does a tactical plan for handling emergencies including communication of such emergencies to communities exist? | | **1** | |  |
|  | Is the overall periodic overseeing of water care function performed? | | **1** | |  |
|  | Is there maintenance and asset management plans? | |  | | **0** |
| **Assessment of operational plans** | Is the attendance of process controllers (and in particular shift workers) at the treatment plant monitored? Is there a clock–in-system for the attendance? | | **1** | |  |
|  | Is the superintendent available to operators on a day-to-day basis? | | **1** | |  |
|  | Is there a substitute system plan put in place when one of the operators is ill or away attending a course? | | **1** | |  |
|  | Are the supervisors and process controllers aware of their job requirements? Are job descriptions available? | | **1** | |  |
| **Maximum possible score for *Management Issues*** | | | **14** | | |
| **Total score attained for *Management Issues*** | | | **13** | | |
| **Weight for *Management Issues*** | | | **0.1** | | |
| **Total weighted score for *Management Issues*** | | | **0.093** | | |

| **NON-TECHNICAL COMPLIANCE (MANAGEMENT) SCORING: Table 11.3** | | | |
| --- | --- | --- | --- |
| **Criterion** | | **Yes =1** | **No = 0** |
| **2. NON-TECHNICAL ASSESSMENT** | | | |
| **2B: Management Practices** | | | |
|  | Do the WSPs use existing local (Emanti/DWAF) or international management practices? Are they implementing them in their water treatment plants? | **1** |  |
|  | Are the WPSs able to understand the existing local or international practices and implementing them correctly? | **1** |  |
|  | When local or international practices are not available, are the WSPs able to draw their own criteria? | **1** |  |
| **Maximum possible score for *Management Practices*** | | **3** | |
| **Total score attained for *Management Practices*** | | **3** | |
| **Weight for *Management Practices*** | | **0.2** | |
| **Total weighted score for *Management Practices*** | | **0.2** | |

| **NON-TECHNICAL COMPLIANCE (MANAGEMENT) SCORING: Table 11.4** | | | | | |
| --- | --- | --- | --- | --- | --- |
| **Criterion** | | **Yes =1** | | **No = 0** | |
| **2. NON-TECHNICAL ASSESSMENT** | | | | | |
| **2C: Human Resources** | | | | | |
| **Personnel at the plant** | Is the number of personnel related to the size of the plant? | |  | | **0** |
|  | Are the needs of process controllers/supervisors and shift workers per plants adequate? | |  | | **0** |
|  | Are there specific guidelines/rules for shift workers and their deployment? | | **1** | |  |
|  | How many operators are there in total? How many shift workers? | | **1** | |  |
| **Qualifications of Personnel** | How many operators with qualifications in the DWAF categories? | | **1** | |  |
|  | Are personnel being correctly applied according to their qualifications? | | **1** | |  |
|  | Are there guidelines or formal rules for upgrading of qualifications? | | **1** | |  |
| **Training Needs** | Are there in service training programmes and policies? What is the in-service training policy of the WSA? | | **1** | |  |
|  | Do process controllers / supervisors / managers know what training opportunities and courses are available? | | **1** | |  |
|  | Are they sent on courses regularly? | | **1** | |  |
|  | Are there specific criteria or formal rules to be qualified for training? | | **1** | |  |
|  | Are there specific promotion requirement protocols in place? | | **1** | |  |
|  | Are there career advancement opportunities? | | **1** | |  |
| **Plant class** | Is the plant classified by DWAF? | | **1** | |  |
|  | Has it been done according to the new classification system? | | **1** | |  |
| **Working Conditions** | Are the responsibilities of process controllers, supervisors and plant managers in line with their salary? | | **1** | |  |
|  | What system is used for overtime management and payment? | | **1** | |  |
|  | What is the WSA policy on conflict management (internal and external)? | | **1** | |  |
|  | Is any medical assistance available for on the job injuries? | | **1** | |  |
|  | Can all workers participate in medical schemes? | | **1** | |  |
|  | Are the retirement/pension-aid benefits, the housing and travel allowance benefits available? | | **1** | |  |
|  | Are the leave benefits (vacation /study/ sick), and the bonus scheme available? | | **1** | |  |
|  | Are there any other incentives? | | **1** | |  |
|  | Are plant personnel satisfied with their working environment? | | **1** | |  |
|  | Are the relationships between supervisors and their subordinates good or poor? | | **1** | |  |
|  | Is there any performance indicators? | | **1** | |  |
| **Maximum possible score for *Human Resources*** | | | **27** | | |
| **Total score attained for *Human Resources*** | | | **25** | | |
| **Weight for *Human Resources*** | | | **0.2** | | |
| **Total weighted score for *Human Resources*** | | | **0.185** | | |

| **NON-TECHNICAL COMPLIANCE (MANAGEMENT) SCORING: Table 11.5** | | | |
| --- | --- | --- | --- |
| **Criterion** | | **Yes =1** | **No = 0** |
| **2. NON-TECHNICAL ASSESSMENT** | | | |
| **2D: Financial Systems** | | | |
| **Financial Systems Sharing in Place** | Which financial systems are in place? | **1** |  |
|  | Are there sufficient funds for operation, maintenance and overtime? | **1** |  |
|  | Do the size of the plants (small versus large) influence the allocation of funds? | **1** |  |
|  | Does the WSA’s budgeting system work in relation to emergency funds, scheduled upgrading/extensions, unscheduled rehabilitation/upgrading? | **1** |  |
| **Information Sharing in Place** | Are there communication channels and transparency between the financial section and the water care section? | **1** |  |
|  | Are regular meetings held? Who attends / chairs these meetings? | **1** |  |
|  | Are decisions taken at consensus / majority? | **1** |  |
|  | Are reports freely available? | **1** |  |
| **Procurement System** | Is the procurement system transparent? | **1** |  |
|  | Is the procurement system open to all? | **1** |  |
|  | Is the water sector involved in the adjudication of tenders? | **1** |  |
| **Maximum possible score for *Financial Systems*** | | **11** | |
| **Total score attained for *Financial Systems*** | | **11** | |
| **Weight for *Financial Systems*** | | **0.1** | |
| **Total weighted score for *Financial Systems*** | | **0.1** | |

| **NON-TECHNICAL COMPLIANCE (MANAGEMENT) SCORING: Table 11.6** | | | |
| --- | --- | --- | --- |
| **Criterion** | | **Yes =1** | **No = 0** |
| **2. NON-TECHNICAL ASSESSMENT** | | | |
| **2E: Communication Systems** | | | |
| **Internal** | Are there sufficient internal communication channels in place, including the needs and shortcomings? | **1** |  |
|  | Are these communication channels effective? | **1** |  |
|  | Is the communication between supervisors and process controllers good or poor? | **1** |  |
|  | Is there any interaction between the maintenance team and the rest of personnel in the water treatment plant? | **1** |  |
|  | Is the communication between management and lower level process controllers good or poor? | **1** |  |
| **External** | Are there sufficient communication channels with other Municipalities and/or Departments? | **1** |  |
|  | Are there sufficient communication channels with consumers? | **1** |  |
|  | Are these external communication channels effective, good or poor? | **1** |  |
| **Maximum possible score for *Communication Systems*** | | **8** | |
| **Total score attained for *Communication Systems*** | | **8** | |
| **Weight for *Communication Systems*** | | **0.2** | |
| **Total weighted score for *Communication Systems*** | | **0.2** | |

| **NON-TECHNICAL COMPLIANCE (MANAGEMENT) SCORING: Table 11.7** | | | |
| --- | --- | --- | --- |
| **Criterion** | | **Yes =1** | **No = 0** |
| **2. NON-TECHNICAL ASSESSMENT** | | | |
| **2F: Audit of safety, health and environmental quality** | | | |
| **Safety Matters** | Are there water and safety management procedures for normal operation, incidents and emergency situations? | **1** |  |
|  | Is there any safety plan at water treatment plants including the safety of process controllers on duties? | **1** |  |
|  | Are there emergency plans in place for chlorine leaks? | **1** |  |
|  | Are there hazardous chemicals and ablution facilities, appropriate fire extinguishers location? | **1** |  |
|  | Are safety meetings held regularly and who attends such meetings? | **1** |  |
| **Health Aspects** | Are there representatives for health and safety of consumers? If yes, are they effective? | **1** |  |
|  | Are there control measures that identify risks and ensure that health based- targets are met in terms of providing safe drinking water to all consumers? | **1** |  |
|  | Are there emergency measures in place for water quality health impact? | **1** |  |
| **Environmental Quality** | Are process controllers satisfied with their working environment? | **1** |  |
|  | Is the working environment appropriate for improving or accelerating the service delivery? | **1** |  |
|  | Is there any strategic plan to improve the quality of the working environment? | **1** |  |
| **Maximum possible score for *SHEQ*** | | **11** | |
| **Total score attained for *SHEQ*** | | **11** | |
| **Weight for *SHEQ*** | | **0.15** | |
| **Total weighted score for *SHEQ*** | | **0.15** | |

| **NON-TECHNICAL COMPLIANCE (MANAGEMENT) SCORING: Table 11.8** | | | |
| --- | --- | --- | --- |
| **Criterion** | | **Yes =1** | **No = 0** |
| **2. NON-TECHNICAL ASSESSMENT** | | | |
| **2G: Community involvement and awareness** | | | |
| **Community Involvement** | Are there protocols involved communities in decision-making? |  | **0** |
|  | Is there an effective reporting system for communities to assist in more rapid response to any water quality incident? |  | **0** |
|  | Is there a Consumer Service to which non-compliance can be reported? | **1** |  |
| **Community Awareness** | Is there a mechanism system to receive and actively address community complaints in a timely fashion? | **1** |  |
|  | Are there procedures for promptly advising of any significant incidents within the drinking water supply including notification of the public health authority? | **1** |  |
|  | Is there any water quality information system available to consumers through annual reports and in the internet? | **1** |  |
| **Maximum possible score for *Community Involvement*** | | **6** | |
| **Total score attained for *Community Involvement*** | | **4** | |
| **Weight for *Community Involvement*** | | **0.05** | |
| **Total weighted score for *Community Involvement*** | | **0.033** | |

| **TABLE 11.9**  **NON-TECHNICAL (MANAGEMENT) COMPLIANCE SCORING:**  **Total Weighted Scoring for Tshedza Package plant 96.1 %** | |
| --- | --- |
| **Compliance Criterion** | **Weighted Score** |
| **2. NON-TECHNICAL ASSESSMENT** | |
| **2A: Management Issues** | **0.093** |
| **2B: Management Practices** | **0.2** |
| **2C: Human Resources** | **0.185** |
| **2D: Financial Systems** | **0.1** |
| **2E: Communication Systems** | **0.2** |
| **2F: Safety, Health and Environmental Quality** | **0.15** |
| **2G: Community Involvement and Awareness** | **0.033** |
| **TOTAL WEIGHTED SCORE** | **96.1** |

| **TABLE 11.10**  **NON-TECHNICAL (MANAGEMENT) COMPLIANCE RATING:** | |
| --- | --- |
| **Total Weighted Score** | **Rating Description** |
| **0 – 50** | ***Class 3 Compliance*:**  Total non-compliance; serious and immediate intervention required (TAC) |
| **50 – 90** | ***Class 2 Compliance:***  Serious challenges requiring attention and improvement |
| **90 – 100** | ***Class 1 Compliance:***  Acceptable compliance |

**Dzingahe Package Plant**

**11 May 2011**

| **TABLE 12.1**  **NON-TECHNICAL (MANAGEMENT) COMPLIANCE SCORING** | |
| --- | --- |
| **Criterion** | **Weight** |
| **2. NON-TECHNICAL ASSESSMENT** | |
| **2A: Management Issues** | **0.1** |
| **2B: Management Practices** | **0.2** |
| **2C: Human Resources** | **0.2** |
| **2D: Financial Systems** | **0.1** |
| **2E: Communication Systems** | **0.2** |
| **2F: Safety, Health and Environmental Quality** | **0.15** |
| **2G: Community Involvement and Awareness** | **0.05** |
| **TOTAL** | **1.0** |

| **NON-TECHNICAL COMPLIANCE (MANAGEMENT) SCORING: Table 12.2** | | | | | |
| --- | --- | --- | --- | --- | --- |
| **Criterion** | | **Yes =1** | | **No = 0** | |
| **2. NON-TECHNICAL ASSESSMENT** | | | | | |
| **2A: Management Issues** | | | | | |
| **Assessment of strategic plans** | Does a strategic plan for the assessment of drinking water supply systems exist? | | **1** | |  |
|  | Are there preventive strategic plans for drinking water quality management? | | **1** | |  |
|  | Is there a strategic plan for recruitment of personnel per water treatment plant available? | | **1** | |  |
|  | Is there a strategic plan for operational monitoring and verification of drinking water system? | | **1** | |  |
|  | Is there a strategic plan for sharing and clarifying roles and responsibilities among all personnel involved in the production of safe drinking water quality? | | **1** | |  |
|  | Does a strategic plan for training and development of water plant personnel, and for community involvement and awareness exist? | | **1** | |  |
|  | Is there any funding strategic plan for implementation of drinking water quality management programmes? | | **1** | |  |
| **Assm of tact plans** | Does a tactical plan for handling emergencies including communication of such emergencies to communities exist? | | **1** | |  |
|  | Is the overall periodic overseeing of water care function performed? | | **1** | |  |
|  | Is there maintenance and asset management plans? | | **1** | |  |
| **Assessment of operational plans** | Is the attendance of process controllers (and in particular shift workers) at the treatment plant monitored? Is there a clock–in-system for the attendance? | | **1** | |  |
|  | Is the superintendent available to operators on a day-to-day basis? | | **1** | |  |
|  | Is there a substitute system plan put in place when one of the operators is ill or away attending a course? | | **1** | |  |
|  | Are the supervisors and process controllers aware of their job requirements? Are job descriptions available? | | **1** | |  |
| **Maximum possible score for *Management Issues*** | | | **14** | | |
| **Total score attained for *Management Issues*** | | | **14** | | |
| **Weight for *Management Issues*** | | | **0.1** | | |
| **Total weighted score for *Management Issues*** | | | **0.1** | | |

| **NON-TECHNICAL COMPLIANCE (MANAGEMENT) SCORING: Table 12.3** | | | |
| --- | --- | --- | --- |
| **Criterion** | | **Yes =1** | **No = 0** |
| **2. NON-TECHNICAL ASSESSMENT** | | | |
| **2B: Management Practices** | | | |
|  | Do the WSPs use existing local (Emanti/DWAF) or international management practices? Are they implementing them in their water treatment plants? | **1** |  |
|  | Are the WPSs able to understand the existing local or international practices and implementing them correctly? | **1** |  |
|  | When local or international practices are not available, are the WSPs able to draw their own criteria? | **1** |  |
| **Maximum possible score for *Management Practices*** | | **3** | |
| **Total score attained for *Management Practices*** | | **3** | |
| **Weight for *Management Practices*** | | **0.2** | |
| **Total weighted score for *Management Practices*** | | **0.2** | |

| **NON-TECHNICAL COMPLIANCE (MANAGEMENT) SCORING: Table 12.4** | | | | | |
| --- | --- | --- | --- | --- | --- |
| **Criterion** | | **Yes =1** | | **No = 0** | |
| **2. NON-TECHNICAL ASSESSMENT** | | | | | |
| **2C: Human Resources** | | | | | |
| **Personnel at the plant** | Is the number of personnel related to the size of the plant? | |  | | **0** |
|  | Are the needs of process controllers/supervisors and shift workers per plants adequate? | |  | | **0** |
|  | Are there specific guidelines/rules for shift workers and their deployment? | | **1** | |  |
|  | How many operators are there in total? How many shift workers? | | **1** | |  |
| **Qualifications of Personnel** | How many operators with qualifications in the DWAF categories? | | **1** | |  |
|  | Are personnel being correctly applied according to their qualifications? | | **1** | |  |
|  | Are there guidelines or formal rules for upgrading of qualifications? | | **1** | |  |
| **Training Needs** | Are there in service training programmes and policies? What is the in-service training policy of the WSA? | | **1** | |  |
|  | Do process controllers / supervisors / managers know what training opportunities and courses are available? | | **1** | |  |
|  | Are they sent on courses regularly? | | **1** | |  |
|  | Are there specific criteria or formal rules to be qualified for training? | | **1** | |  |
|  | Are there specific promotion requirement protocols in place? | | **1** | |  |
|  | Are there career advancement opportunities? | | **1** | |  |
| **Plant class** | Is the plant classified by DWAF? | | **1** | |  |
|  | Has it been done according to the new classification system? | | **1** | |  |
| **Working Conditions** | Are the responsibilities of process controllers, supervisors and plant managers in line with their salary? | |  | | **0** |
|  | What system is used for overtime management and payment? | | **1** | |  |
|  | What is the WSA policy on conflict management (internal and external)? | | **1** | |  |
|  | Is any medical assistance available for on the job injuries? | | **1** | |  |
|  | Can all workers participate in medical schemes? | | **1** | |  |
|  | Are the retirement/pension-aid benefits, the housing and travel allowance benefits available? | | **1** | |  |
|  | Are the leave benefits (vacation /study/ sick), and the bonus scheme available? | | **1** | |  |
|  | Are there any other incentives? | | **1** | |  |
|  | Are plant personnel satisfied with their working environment? | | **1** | |  |
|  | Are the relationships between supervisors and their subordinates good or poor? | | **1** | |  |
|  | Is there any performance indicators? | | **1** | |  |
| **Maximum possible score for *Human Resources*** | | | **27** | | |
| **Total score attained for *Human Resources*** | | | **24** | | |
| **Weight for *Human Resources*** | | | **0.2** | | |
| **Total weighted score for *Human Resources*** | | | **0.178** | | |

| **NON-TECHNICAL COMPLIANCE (MANAGEMENT) SCORING: Table 12.5** | | | |
| --- | --- | --- | --- |
| **Criterion** | | **Yes =1** | **No = 0** |
| **2. NON-TECHNICAL ASSESSMENT** | | | |
| **2D: Financial Systems** | | | |
| **Financial Systems Sharing in Place** | Which financial systems are in place? | **1** |  |
|  | Are there sufficient funds for operation, maintenance and overtime? | **1** |  |
|  | Do the size of the plants (small versus large) influence the allocation of funds? | **1** |  |
|  | Does the WSA’s budgeting system work in relation to emergency funds, scheduled upgrading/extensions, unscheduled rehabilitation/upgrading? | **1** |  |
| **Information Sharing in Place** | Are there communication channels and transparency between the financial section and the water care section? | **1** |  |
|  | Are regular meetings held? Who attends / chairs these meetings? | **1** |  |
|  | Are decisions taken at consensus / majority? | **1** |  |
|  | Are reports freely available? | **1** |  |
| **Procurement System** | Is the procurement system transparent? | **1** |  |
|  | Is the procurement system open to all? | **1** |  |
|  | Is the water sector involved in the adjudication of tenders? | **1** |  |
| **Maximum possible score for *Financial Systems*** | | **11** | |
| **Total score attained for *Financial Systems*** | | **11** | |
| **Weight for *Financial Systems*** | | **0.1** | |
| **Total weighted score for *Financial Systems*** | | **0.1** | |

| **NON-TECHNICAL COMPLIANCE (MANAGEMENT) SCORING: Table 12.6** | | | |
| --- | --- | --- | --- |
| **Criterion** | | **Yes =1** | **No = 0** |
| **2. NON-TECHNICAL ASSESSMENT** | | | |
| **2E: Communication Systems** | | | |
| **Internal** | Are there sufficient internal communication channels in place, including the needs and shortcomings? | **1** |  |
|  | Are these communication channels effective? | **1** |  |
|  | Is the communication between supervisors and process controllers good or poor? | **1** |  |
|  | Is there any interaction between the maintenance team and the rest of personnel in the water treatment plant? | **1** |  |
|  | Is the communication between management and lower level process controllers good or poor? | **1** |  |
| **External** | Are there sufficient communication channels with other Municipalities and/or Departments? | **1** |  |
|  | Are there sufficient communication channels with consumers? | **1** |  |
|  | Are these external communication channels effective, good or poor? | **1** |  |
| **Maximum possible score for *Communication Systems*** | | **8** | |
| **Total score attained for *Communication Systems*** | | **8** | |
| **Weight for *Communication Systems*** | | **0.2** | |
| **Total weighted score for *Communication Systems*** | | **0.2** | |

| **NON-TECHNICAL COMPLIANCE (MANAGEMENT) SCORING: Table 12.7** | | | |
| --- | --- | --- | --- |
| **Criterion** | | **Yes =1** | **No = 0** |
| **2. NON-TECHNICAL ASSESSMENT** | | | |
| **2F: Audit of safety, health and environmental quality** | | | |
| **Safety Matters** | Are there water and safety management procedures for normal operation, incidents and emergency situations? | **1** |  |
|  | Is there any safety plan at water treatment plants including the safety of process controllers on duties? | **1** |  |
|  | Are there emergency plans in place for chlorine leaks? | **1** |  |
|  | Are there hazardous chemicals and ablution facilities, appropriate fire extinguishers location? | **1** |  |
|  | Are safety meetings held regularly and who attends such meetings? | **1** |  |
| **Health Aspects** | Are there representatives for health and safety of consumers? If yes, are they effective? | **1** |  |
|  | Are there control measures that identify risks and ensure that health based- targets are met in terms of providing safe drinking water to all consumers? | **1** |  |
|  | Are there emergency measures in place for water quality health impact? | **1** |  |
| **Environmental Quality** | Are process controllers satisfied with their working environment? | **1** |  |
|  | Is the working environment appropriate for improving or accelerating the service delivery? | **1** |  |
|  | Is there any strategic plan to improve the quality of the working environment? | **1** |  |
| **Maximum possible score for *SHEQ*** | | **11** | |
| **Total score attained for *SHEQ*** | | **11** | |
| **Weight for *SHEQ*** | | **0.15** | |
| **Total weighted score for *SHEQ*** | | **0.15** | |

| **NON-TECHNICAL COMPLIANCE (MANAGEMENT) SCORING: Table 12.8** | | | |
| --- | --- | --- | --- |
| **Criterion** | | **Yes =1** | **No = 0** |
| **2. NON-TECHNICAL ASSESSMENT** | | | |
| **2G: Community involvement and awareness** | | | |
| **Community Involvement** | Are there protocols involved communities in decision-making? |  | **0** |
|  | Is there an effective reporting system for communities to assist in more rapid response to any water quality incident? | **1** |  |
|  | Is there a Consumer Service to which non-compliance can be reported? | **1** |  |
| **Community Awareness** | Is there a mechanism system to receive and actively address community complaints in a timely fashion? | **1** |  |
|  | Are there procedures for promptly advising of any significant incidents within the drinking water supply including notification of the public health authority? | **1** |  |
|  | Is there any water quality information system available to consumers through annual reports and in the internet? | **1** |  |
| **Maximum possible score for *Community Involvement*** | | **6** | |
| **Total score attained for *Community Involvement*** | | **5** | |
| **Weight for *Community Involvement*** | | **0.05** | |
| **Total weighted score for *Community Involvement*** | | **0.042** | |

| **TABLE 12.9**  **NON-TECHNICAL (MANAGEMENT) COMPLIANCE SCORING:**  **Total Weighted Scoring for Dzingahe Package plant 97.0 %** | |
| --- | --- |
| **Compliance Criterion** | **Weighted Score** |
| **2. NON-TECHNICAL ASSESSMENT** | |
| **2A: Management Issues** | **0.1** |
| **2B: Management Practices** | **0.2** |
| **2C: Human Resources** | **0.178** |
| **2D: Financial Systems** | **0.1** |
| **2E: Communication Systems** | **0.2** |
| **2F: Safety, Health and Environmental Quality** | **0.15** |
| **2G: Community Involvement and Awareness** | **0.042** |
| **TOTAL WEIGHTED SCORE** | **0.97** |

| **TABLE 12.10**  **NON-TECHNICAL (MANAGEMENT) COMPLIANCE RATING:** | |
| --- | --- |
| **Total Weighted Score** | **Rating Description** |
| **0 – 50** | ***Class 3 Compliance*:**  Total non-compliance; serious and immediate intervention required (TAC) |
| **50 – 90** | ***Class 2 Compliance:***  Serious challenges requiring attention and improvement |
| **90 – 100** | ***Class 1 Compliance:***  Acceptable compliance |

**Table S2.** Technical compliance rating of the treatment plants during the first between August 2008 and June 2009 and the second assessment between November and December 2010.

| **Problem area** | **Description** | **Direct causes** | **Underlying causes** | **Implications/ Consequences** | **Score (1-5)** | **Corrective measures** | **Date implemented** |
| --- | --- | --- | --- | --- | --- | --- | --- |
| 1A. Design aspect | Basic knowledge of water treatment principles such as the determination of the flow rate of water, calculation of coagulant and chorine dosages | Lack of basic knowledge of water treatment principles such as the determination of the flow rate of water, calculation of coagulant and chorine dosages | Lack of laboratory instruments  Inability to dose the correct quantity of chemicals | Threat to consumer’s life | 3 | Proper allocations of funds for operations and maintenance | First assessment |
| 1D. Plant Monitoring Practices | Determination of the chlorine demand of the water and measurement of free chlorine residual concentrations in the final water, as well as measurement of various determinants | Inability to determine the chlorine demand of the water and measurement of free chlorine residual concentrations in the final water, as well as measurement | Lack of resources such as laboratory instruments  Insufficient budget allocations | Process controllers may supply water that is not safe for domestic consumption | 3 | Proper allocations of funds for operations and maintenance |  |
| 1F. Risk Management Practices | Comprehensive risk-based risk register | Lack of risk register | Lack of relevant skills to develop the risk register | Unsafe working environment for process controllers | 4 | Development of risk register |  |

**Table S3.** Ranking of the Problem Areas in Priority Order after the first assessment.

|  |  | **1A. Design aspect** | **1D. Plant Monitoring Practices** | **1F. Risk Management Practices** |
| --- | --- | --- | --- | --- |
| 1 | Insignificant Health Consequence |  |  |  |
| 2 | Minor Health Consequence |  |  |  |
| 3 | Moderate Health Consequence | 3 | 3 |  |
| 4 | Major Health Consequence |  |  | 4 |
| 5 | Catastrophic Health Consequence |  |  |  |
